# In Vitro and In Vivo Enhancement of Temozolomide Effect in Human Glioblastoma by Non-Invasive Application of Cold Atmospheric Plasma

**DOI:** 10.3390/cancers13174485

**Published:** 2021-09-06

**Authors:** Vikas Soni, Manish Adhikari, Hayk Simonyan, Li Lin, Jonathan H. Sherman, Colin N. Young, Michael Keidar

**Affiliations:** 1 Department of Mechanical and Aerospace Engineering, MPNL, The George Washington University, Washington, DC 20052, USA; vik1808@gwmail.gwu.edu (V.S.); manishadhikari85@gmail.com (M.A.); lilin@gwmail.gwu.edu (L.L.); jsherman0620@gmail.com (J.H.S.); 2 Department of Pharmacology and Physiology, School of Medicine and Health Sciences, The George Washington University, Washington, DC 20052, USA; hayksimonyan@gwu.edu

**Keywords:** cold atmospheric plasma, glioblastoma, reactive oxygen and nitrogen species, electromagnetic waves, bone, temozolomide, cancer therapy, xenografts, sensitization, plasma medicine, RNA-sequencing, gene functional enrichment analysis

## Abstract

**Simple Summary:**

Glioblastoma (GBM) is an aggressive type of brain cancer, with which only 25% of patients survive for more than one year. Treatment of GBM has remained a challenge due to its resistance to chemotherapy. Here, we aimed to assess the potential for a combination therapy of cold atmospheric plasma (CAP) and Temozolomide (TMZ) to treat GBM. We confirmed the effect of co-treatment on different GBM cell lines in vitro and determined the enhancement of the effect of TMZ and the potential sensitization of GBM to CAP + TMZ in murine models in vivo. We found that co-treatment with CAP + TMZ inhibited GBM significantly compared to single treatment with CAP or TMZ alone. We provided further evidence related to the bone penetration of reactive oxygen and nitrogen species, as well as electromagnetic waves generated by CAP. RNA sequencing further indicated an effect of CAP + TMZ on cell cycle pathways. Collectively, these findings point to potential non-invasive translational approaches to target GBM in the future.

**Abstract:**

Glioblastoma (GBM) is one of the most aggressive forms of adult brain cancers and is highly resistant to treatment, with a median survival of 12–18 months after diagnosis. The poor survival is due to its infiltrative pattern of invasion into the normal brain parenchyma, the diffuse nature of its growth, and its ability to quickly grow, spread, and relapse. Temozolomide is a well-known FDA-approved alkylating chemotherapy agent used for the treatment of high-grade malignant gliomas, and it has been shown to improve overall survival. However, in most cases, the tumor relapses. In recent years, CAP has been used as an emerging technology for cancer therapy. The purpose of this study was to implement a combination therapy of CAP and TMZ to enhance the effect of TMZ and apparently sensitize GBMs. In vitro evaluations in TMZ-sensitive and resistant GBM cell lines established a CAP chemotherapy enhancement and potential sensitization effect across various ranges of CAP jet application. This was further supported with in vivo findings demonstrating that a single CAP jet applied non-invasively through the skull potentially sensitizes GBM to subsequent treatment with TMZ. Gene functional enrichment analysis further demonstrated that co-treatment with CAP and TMZ resulted in a downregulation of cell cycle pathway genes. These observations indicate that CAP can be potentially useful in sensitizing GBM to chemotherapy and for the treatment of glioblastoma as a non-invasive translational therapy.

## 1. Introduction

Glioblastoma multiforme (GBM) is a highly complex brain tumor and patient prognosis is often poor. In recent years, advancements in technology have not significantly improved the overall patient survival rate. GBM’s recurrence is unpredictable; its control, spread, and metastasis are often uncertain and dependent on individual cases of patients [1,2]. Moreover, it has a high tendency to relapse. Thus, GBM can be very difficult to treat, and treatments such as surgery, chemotherapy, radiation, and immunotherapy may slow the tumor progression, but may not cure it. Hence, the requirement of a novel modality regime is necessary.

Plasma medicine is an emerging interdisciplinary field that combines physics, biomedical, and clinical applications. Cold atmospheric plasma (CAP) is a partly ionized gas formed at atmospheric pressure, with quasi-neutral charges composed of positive and negative charged ions, charged radicals, neutral atoms, and ultraviolet (UV) photons [3]. CAP has gained a lot of interest due to its extraordinary ability to influence biological processes, and an advantage of CAP is that it operates at room temperature. Through the electric breakdown of gas such as helium (He) or argon (Ar) between electrodes driven by a specific voltage, plasma is generated [4,5]. In general, CAP contains a reactive combination of electrons, ions, excited atoms, molecules, reactive species (e.g., OH, O, H_2_O_2_, O_3_, NO, NO_2_, etc.), ultraviolet (UV) photons, and electromagnetic (EM) waves, collectively termed reactive agents (RAs). Since plasma generates a wide range of RAs, it has been used in many fields including medical applications, dentistry, the biomedical sterilization of tools and dental instruments, wound healing, dermatology, and clinical oncology [6,7,8]. In the context of oncology, some reports have found that exposing cancerous cells to CAP leads to the generation of free radicals and RAs that are known to be toxic to a cancer cell and can induce the apoptotic cell death of GBM cells [9,10,11].

Temozolomide is used as the gold standard for the treatment of high-grade GBM. Here, we reasoned that combining CAP with the commonly used chemotherapy drug temozolomide (TMZ) would enhance the treatment of GBM [12,13]. TMZ can extend the overall patient survival rate. However, most cases eventually develop resistance to TMZ due to the O6-methylguanine-DNA methyltransferase (MGMT) promoter, leading to repair and resistance [14,15,16]. Thus, a small population of cells can contribute to resistance, and recurrence of the tumor may be observed. TMZ resistance in GBM is modulated by DNA repair caused by the MGMT gene. Epigenetic silencing of the MGMT promoter by methylation compromises MGMT-based DNA repair, leading to longer survival rates after TMZ administration, whereas if the MGMT promoter is unmethylated, it is associated with TMZ resistance in some, but not all, GBM tumors. Hence, it is important to sensitize cells to TMZ using novel treatments to overcome the issue of resistance [14,15,16,17,18,19,20]. Therefore, using a series of in vitro and in vivo experiments in GBM models, we introduced co-treatment with CAP and TMZ through the bone as a new approach and non-invasive strategy to potentially sensitize GBM to chemotherapy [21,22,23]. Furthermore, we also demonstrated the utility of a novel, non-invasive He-based CAP jet to target GBM through the skull [9].

## 2. Results

### 2.1. Characterization of CAP Jet by Optical Emission Spectroscopy (OES)

**Figure 1:**
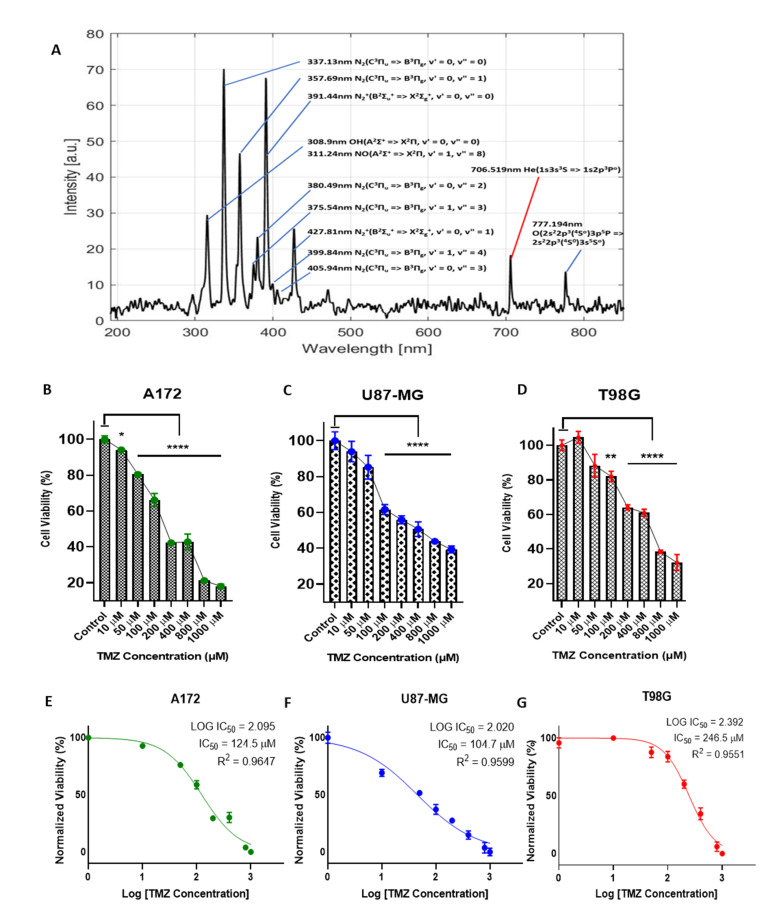
(**A**) Optical Emission Spectroscopy of the CAP jet using SpectraWiz^®^ showing most peaks of nitrogen C3Πu => B3Πg ranging from the spectral region of about 300–490 nm. Relative cell viability (%) for the GBM tumor cell line A172 (**B**), U87-MG (**C**), and T98G (**D**) 72 h following a single TMZ treatment. The log IC50 after 5-day TMZ treatment of A172 (**E**), U87-MG (**F**), and T98G (**G**) cell lines. Cell viability measures were collected 96 h after TMZ treatment. All data represent the mean ± SE, and all experiments were performed in quadruplets. Data were analyzed using Student’s *t*-test * *p* < 0.05; ** *p* < 0.01; and **** *p* < 0.0001, ns—not significant vs. untreated control. n = 16.

The CAP device setup and circuit diagram are illustrated in the graphical abstract. The CAP jet was characterized using a SpectraWiz^®^ spectroscopy device. Figure 1A illustrates the representative spectra of the CAP jet used. The OES showed a mixture of peaks at various wavelengths at 297 nm oxygen (O_2_), 308.9 nm hydroxyl radicals (OH), 311.24 nm nitric oxide (NO), 337.13 nm nitrogen (N_2_), 357.69 nm N_2_, 375.54 nm N_2_, 380.49 nm N_2_, 391.44 nm N_2_+, 399.84 nm N_2_, 405.94 nm N_2_, 427.81 nm N_2_+, 706.519 nm helium (He) (1s3s3S => 1s2p3Po), and 777.194 nm atomic oxygen (O). The identification of the spectra was performed by analyzing the peaks as previously described [24,25,26]. Most peaks of N_2_ corresponded to the transition state between the electronic states C3Πu => B3Πg, ranging from the spectral region of about 300–490 nm. These could be designated as the reactive nitrogen species (RNS) range, whereas peaks at 297 nm and 777 nm were designated as reactive oxygen species (ROS), and peaks from 250 nm to about 490 nm were known as reactive oxygen and nitrogen species (RONS). The peak identified at 308.9 nm could be defined as the OH species transitioning from A2Σ+ => X2Π state. The He peaks usually arise between 600 nm and 750 nm and were identified at 706.5 nm with a transition state of 1s3s3S => 1s2p3Po. The N_2_ peaks dominated the spectra due to the He supply and ambient air, indicating a high amount of ROS/RNS due to its lower ionization energy than He.

### 2.2. The Effects of TMZ on Sensitive and Resistant Cell Lines

A172, U87-MG (TMZ-sensitive), and T98G (TMZ-resistant) [14,15,16,17,18,19,20] GBM cells were grown to 60–80% confluency, and 5000 cells per well were seeded in a 96-well flat-bottom plate. Cells were treated with serial dilutions of TMZ from a range of 0–1000 µM. To investigate any potential differences of cell death kinetics between a sensitive and a resistant cell line, an MTT assay was performed to demonstrate the IC50 of TMZ. Figure 1B–D show the IC50 of the A172, U87-MG, and T98G cell lines after 72 h of treatment, whereas Figure 1E–G represent the drug log concentration versus the viability (%) after 5 days of treatment. Determination of the IC50 in different cell lines gave values ranging from 200 to 400 μM for 72 h of treatment and a higher IC50 value indicated the resistance of T98G cells when compared to the A172 and U87-MG cell lines. Increasing the time of TMZ treatment to 5 days decreased the IC50 significantly in cell lines that were TMZ sensitive and a lower IC50 was observed in the resistant cell line as well, indicating that the time of treatment is an essential factor in determining the drug toxicity and cell viability. The log IC50, after the 5-day treatment of TMZ in the sensitive cell line A172, was found to be 2.095; (R^2^ = 0.96) = ~125 μM, for U87-MG was found to be 1.782; (R^2^ = 0.96) = ~105 μM, and the log IC50 for T98G was found to be 2.392; (R^2^ = 0.95) = ~247 μM.

### 2.3. Combination Effect of CAP Jet in Association with TMZ on GBM In Vitro

**Figure 2:**
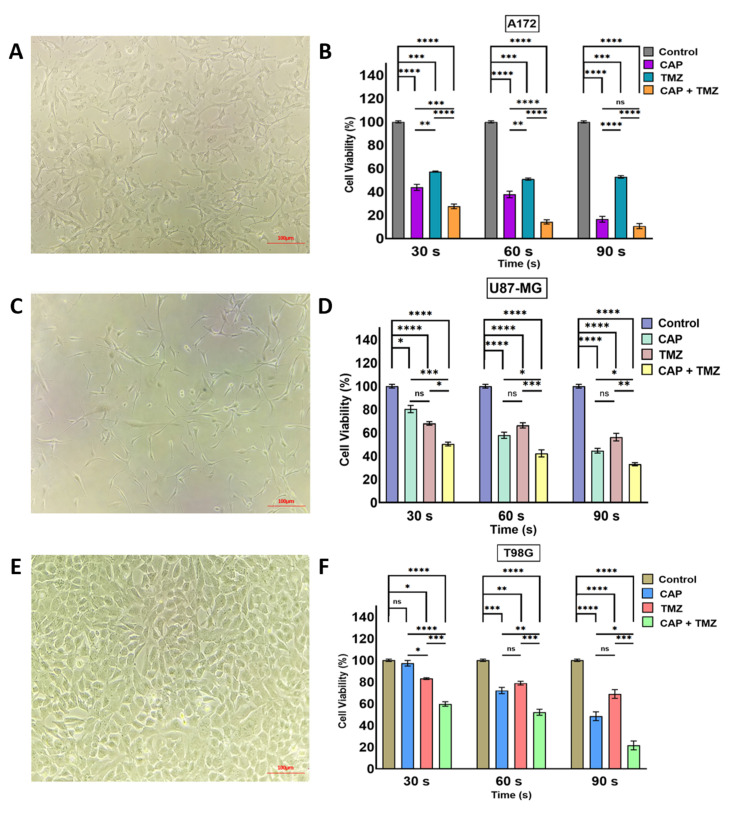
(**A**) Bright field image of A172 cells 40–50% confluent at 10× magnification before seeding; (**B**) relative growth viability for A172 cells after 72 h; (**C**) bright field image of U87-MG cells 40–50% confluent at 10× magnification; (**D**) relative cell viability for U87-MG cells after 72 h; (**E**) bright field image of T98G cells at 80–90% confluency; (**F**) relative cell viability of T98G cells after 72 h. All of the cell lines were treated with CAP, TMZ, and the co-treatment of CAP + TMZ for 30, 60 and 90 s. The ratios of the treatment groups were normalized for each cell line to their relative untreated controls (0 s). A two-way ANOVA was performed to identify the statistical significance compared to the control and was followed by Dunnett’s multiple comparisons post hoc test. * *p* < 0.05; ** *p* < 0.01; *** *p* < 0.001; and **** *p* < 0.0001, ns—not significant vs. untreated control. n = 16.

We next investigated the responsiveness of three GBM cell lines (two TMZ sensitive and one resistant; A172, U87-MG, and T98G, respectively) [14,15,16,17,18,19,20] to direct CAP jet treatment at different time exposures of 30, 60, and 90 s (frequency of 12.5 kHz and 10 V and He flow rate of 3–4 LPM) under TMZ (400 μM) exposure alone and a combination treatment of CAP + TMZ [13,22,23]. The four treatment conditions for each time point were: control (untreated), CAP jet treated, TMZ treated, and co-treatment with CAP + TMZ. The motivation to use 400 μM TMZ was to compare at least one in vitro experiment and dose to in vivo studies as a single CAP treatment and TMZ dose approximately equivalent to an in vivo dose (based on BSA). As shown in Figure 2, the cell viability after 72 h incubation with TMZ inhibited cell growth by ~16–24% for A172, 18–23% for U87-MG, and 17–25% for T98G cells. CAP alone also reduced cell viability for all treatment times, with 90 s demonstrating the largest effect, which is consistent with previous reports [27]. Interestingly, combination treatment with CAP + TMZ further inhibited the viability of all cell lines by ~10–30% relative to CAP or TMZ treatment alone. For example, as shown in Figure 2B,D,F, the combination of CAP + TMZ inhibited the viability by approximately 60% (30 s), 86% (60 s) and 89% (90 s) for A172 cells; 51% (30 s), 58% (60 s) and 67% (90 s) for U87-MG cells; and 60% (30 s), 48% (60 s) and 78% (90 s) for T98G cells relative to the untreated control.

### 2.4. Sensitization of GBM Cells to TMZ with the Co-Treatment of CAP + TMZ In Vitro

**Figure 3:**
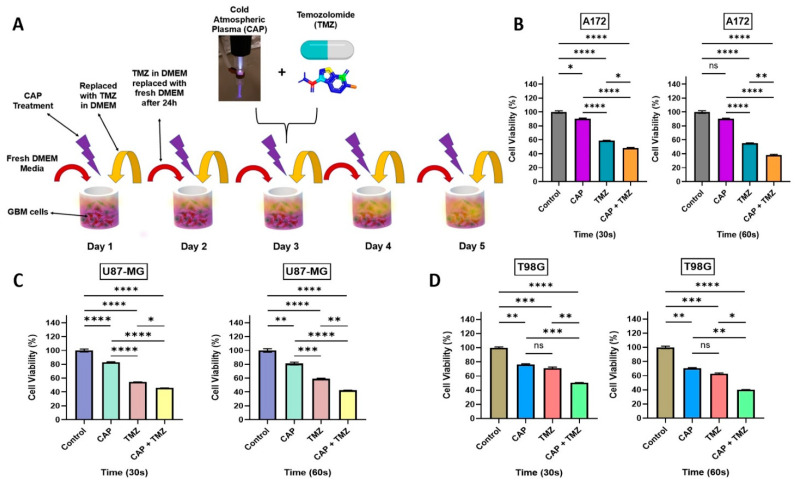
(**A**) Schematic representation of the sensitization process; (**B**) the impact of a 5-day treatment on A172 cells after 72 h, relative viability (%) versus time, 30 s on the left and 60 s on the right; (**C**) relative cell viability (%) of TMZ-sensitive GBM cells U87-MG to the cytotoxicity of TMZ, treatment for 30 s (left) and 60 s (right); (**D**) relative cell viability (%) for a TMZ-resistant cell line T98G after 72 h following a 5-day CAP and TMZ treatment. All the cell lines were treated with CAP, TMZ, and the co-treatment of CAP + TMZ for 30 and 60 s, the ratios of treatment groups, were normalized for each cell line to their relative untreated control (0 s) treatment in DMEM. All data represent the mean ± SE. A one-way ANOVA was performed to identify the statistical significance compared to control and was followed by Tukey’s multiple comparisons post hoc test. * *p* < 0.05; ** *p* < 0.01; *** *p* < 0.001; and **** *p* < 0.0001, ns—not significant vs. untreated control. n = 10.

The findings presented in Figure 2 indicate that the cytotoxicity in TMZ-sensitive and resistant cells can be enhanced by co-treatment with CAP [13]. Building upon this, we next determined a longer-term potential sensitization effect over 5-day treatment. Each day, the cells were treated with CAP (30 or 60 s) and the CAP-treated media was immediately aspirated and replaced with fresh DMEM. TMZ (105, 125, and 247 μM) was added to TMZ and CAP + TMZ groups based on the IC50 values of TMZ for each cell line established in Section 2.2 [28,29] (105, 125, and 247 μM for the A172, U87-MG, and T98G, respectively). On day 2, TMZ was removed and replaced with fresh DMEM before treatment with CAP. After the CAP treatment, the media was removed and TMZ was added; this process was repeated for 5 days. Figure 3A shows a schematic illustration of the sensitization procedure. Daily 5-day CAP treatment resulted in a significant reduction in cell viability across the GBM cell lines, demonstrating that CAP treatment still has an effect even when the CAP-treated media is removed, which contains plasma species. As anticipated, TMZ treatment alone resulted in a ~50–60% reduction in cell viability relative to controls. However, pre-treatment with the CAP jet for 30 and 60 s followed by TMZ treatment enhanced the TMZ-induced cytotoxicity, indicating that the CAP jet sensitizes GBM cells to TMZ [13,22,23,30]. Interestingly, this potential sensitization effect was evident not only in TMZ-sensitive, but also TMZ-resistant (T98G) cell lines.

### 2.5. Measurement of OES and Electromagnetic Emission from the CAP Jet Using Bone as a Barrier

**Figure 4:**
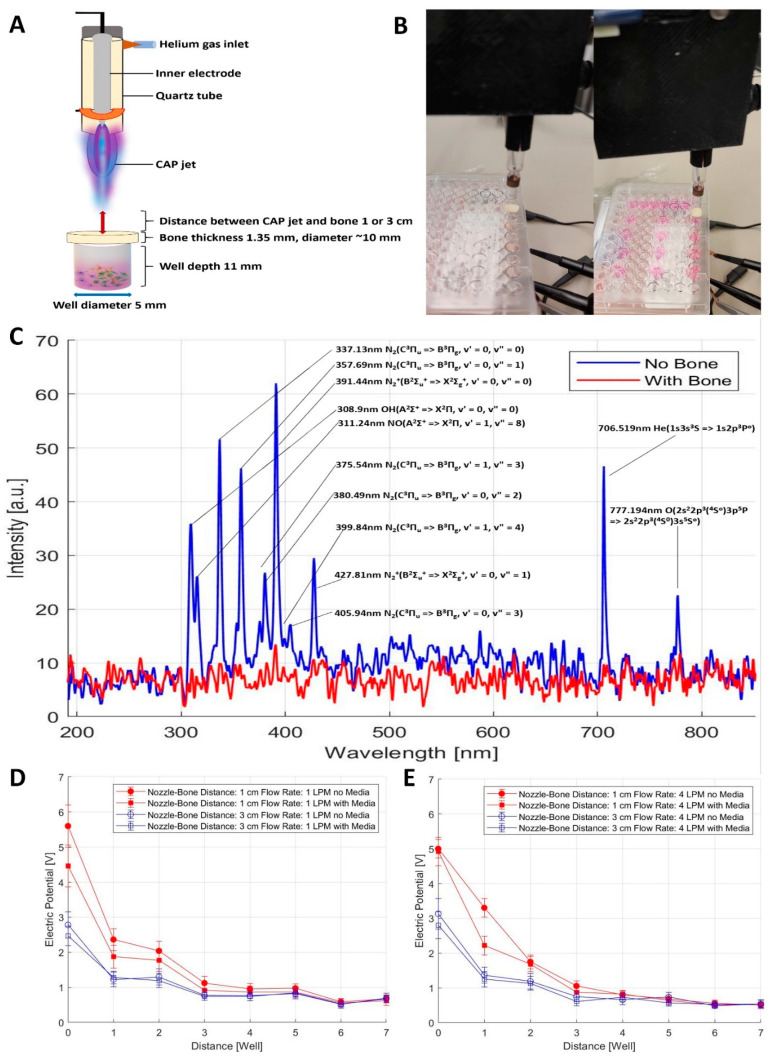
(**A**) Schematic illustration of the experimental setup for the measurement of OES and EM emission through human fibula bone. (**B**) Images of treatment and measurement without media (left) and with the media (right). (**C**) OES measurements of the CAP jet with (red) and without (blue) the bone. EM wave emission measurements at 1 LPM (**D**) and 4 LPM (**E**). The distance between the plume and bone were 1 cm (labeled as red) and 3 cm (blue). The well labeled as ‘0’ indicates the well that was targeted with CAP through the bone. n = 10.

We next investigated the penetration of CAP and EM waves through bone. We used a human fibula bone as a barrier by placing it on top of a 96-well plate and treating the cells with a CAP jet. The objective to this was to demonstrate whether CAP can penetrate through bone and whether this novel method can be applied to murine models as a non-invasive therapy. A schematic representation of the experimental setup is shown in Figure 4A,B. The OES of the system was measured to analyze the representative spectra of the CAP jet used. The OES of the treatment well without the bone showed a mixture of peaks at various wavelengths (Figure 4C) at 297 nm oxygen (O_2_), 308.9 nm hydroxyl radicals (OH), 311.24 nm nitric oxide (NO), 337.13 nm nitrogen (N_2_), 357.69 nm N_2_, 375.54 nm N_2_, 380.49 nm N_2_, 391.44 nm N_2_+, 399.84 nm N_2_, 405.94 nm N_2_, 427.81 nm N_2_+, 706.519 nm helium (He) (1s3s3S => 1s2p3Po), and 777.194 nm atomic oxygen (O) consistent with our results in Section 2.1 (Figure 1A). The identification of the spectra was performed by analyzing the peaks as previously described [24,25,26]. Interestingly, there were no peaks seen in the treatment sample when the bone was placed on top of the well. The measurements were carried out with the fiber optic probe placed at the bottom of the well and furthermore, on the side of the well, indicating that bone blocked most of the CAP generated species, shown in Figure 4C in red. Blue indicates prominent peaks of RONS measured without the bone.

We next investigated the electromagnetic emission of the CAP jet through the bone. A schematic representation of the experimental setup is shown in Figure 4A. A human bone fibula with a thickness of 1.35 mm was used. The distance of the plume from the bone was maintained at 1 or 3 cm with a flow rate of either 1 or 4 LPM. Figure 4B illustrates CAP jet EM emission measurements using a piece of bone with a 10 mm diameter to cover a single well. We obtained measurements with and without the media based on our previous findings, demonstrating that the EM waves caused more cytotoxicity to the cells without media [21]. To avoid any CAP exposure to the other wells, a gap of two wells was included between treatment samples. Electromagnetic emission includes three major parts. The first comes from the electric field of the streamer impacting the bone. The streamer head usually carries an electric potential close to the anode with a potential drop over a megaohm-resistance plasma track [31,32]. Such an impact occurs at the same frequency as the discharge frequency. The second one comes from the plasma oscillation at GHz. Considering that the mean electron density of the CAP jet is about 10^12^ cm^−3^ [33,34], the plasma frequency is usually between 10 GHz and 30 GHz. The frequency can be computed by, $\omega =\sqrt{\frac{n_{e}e^{2}}{m_{e}\varepsilon }}$, where *ω* is the plasma frequency, *n_e_* is the electron density, *e* is the unit charge, *m_e_* is the mass of an electron, and *ε* is the permittivity [35]. However, the power of such an emission is relatively low [21]. The third emission is UV–Vis, which can be quantified by OES. To measure the first emission in the radio frequency range, we attached an electrode beneath each well. These electrodes thus receive extra electric potential peaks when the streamer approaches the bone. The potential values are shown in Figure 4D,E. Considering the distance between bone and well bottom is about 1 cm, the average electric field in the well with direct contact to CAP is around 5 V/cm. Therefore, according to the inverse-square law, the electric field across the bone could be estimated to be around 500 V/cm for a ~1 mm thick bone. As expected, wells located further away from the treated well received a weaker electric field, as shown in Figure 4D,E. We also established that maximum emission was observed directly from the well treated with CAP through the bone, indicated as point 0 (treatment well 1) in the graph. We removed the media prior to the treatment with CAP and noticed that without media, treatments were more effective than with media, indicating that EM waves penetrate the bone and cause more cytotoxicity compared to media treatments [21]. Figure 4D,E show the distance of wells versus the electric potential (V), indicated as red for 1 cm and blue for 3 cm distance. The corresponding electric potential for 1 LPM at distances of 1 and 3 cm without media was found to be 5.6 V and 2.8 V, respectively, showing that distance plays a role in EM emission. Hence, considering these results, all the in vivo treatments were performed at the distance of 1 cm from the skull of mice, as we achieved the maximum emission in this condition. These results indicate that EM waves can penetrate bone.

### 2.6. Penetration of RAs and EM Waves Using Bone as a Barrier for In Vitro CAP Jet Treatment

**Figure 5:**
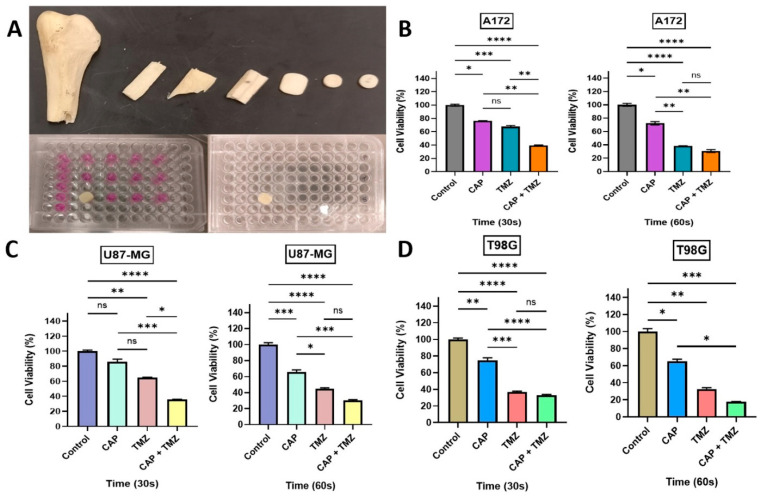
(**A**) Picture of a human bone fibula sectioned into smaller pieces to fit 96-well flat-bottom plates. Relative growth viability (%) of A172 (**B**) U87-MG (**C**), and T98G (**D**) cells 72 h after 30 or 60 s CAP treatment with or without TMZ co-treatment. All of the cell lines were treated without media, and a bone section was placed on top of the wells. The treatment groups for each cell line were normalized to their relative untreated controls (0 s). A one-way ANOVA was performed with Tukey’s multiple comparison post hoc test. * *p* < 0.05; ** *p* < 0.01; *** *p* < 0.001; and **** *p* < 0.0001, ns—not significant vs. untreated control. n = 16.

The findings presented in Figure 2, Figure 3 and Figure 4 indicate that the cytotoxicity in TMZ-sensitive and resistant cells can be enhanced by co-treatment with CAP and the CAP jet can penetrate through the bone. It is well known that CAP has the tendency to penetrate the skin [36,37,38,39], and in recent years, there has been literature suggesting the penetration of plasma species and EM waves through bone [40,41,42]. This is critical for the non-invasive treatment of brain cancers, where CAP delivery needs to penetrate the skull. To further investigate the penetration of CAP-derived plasma species and EM waves through bone, we used a human fibula bone as a barrier by placing it on top of a 96-well plate and treating the cells with a CAP jet. The media was removed individually for each well just prior to the treatment with CAP, and it was ensured that only the well that was being treated did not have media. The control sample corresponding to the treatment well also did not have the media during the length of treatment (30 and 60 s) to keep consistency with the samples. The rest of the wells had the media to avoid stress or dehydration to the cells. As shown in Figure 5A, the bone was cut into small pieces, shaped, and fined to match the width/thickness of mouse skull bone (~1.35 mm thickness). CAP treatment for 30 or 60 s, relative to untreated controls, showed noticeable growth inhibition in A172, U87-MG, and T98G cell lines (Figure 5B–D) indicating that CAP has the tendency to penetrate through bone. Note that the effect associated with CAP in this case is likely through EM waves as reactive species might not directly penetrate bone of such thickness [43,44,45]. Similarly, TMZ treatment alone resulted in a reduction in cell viability across all cell lines. However, co-treatment with CAP and TMZ enhanced the singular effect of each treatment.

### 2.7. Molecular Analysis of Key Genes in U87-MG Cells Using RNA-Seq

**Figure 6:**
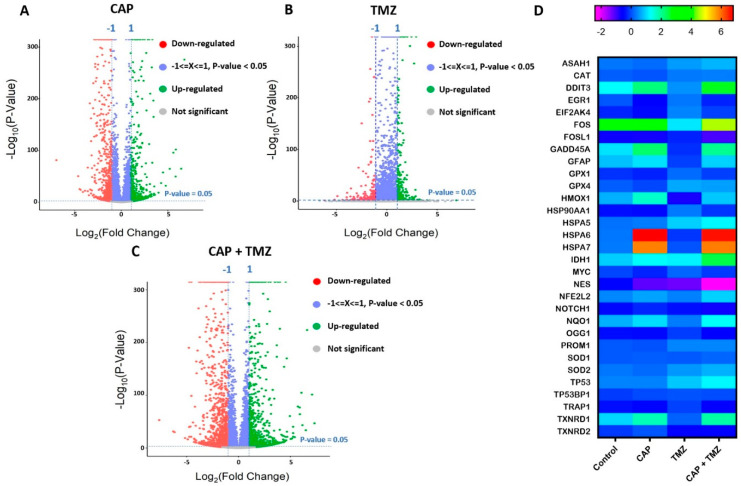
(**A**–**C**) Volcano plots of differentially expressed genes in U87-MG cells post-treatment with CAP, TMZ or co-treatment. (**D**) Heatmap profile of key genes in stress response and markers of GBM. Significance: log2 fold change value ≥ ±2.0 and *p*-value ≤ 0.05. n = 9.

We next investigated the effect of CAP and TMZ in U87-MG cells at the *mRNA* level by RNA-seq analysis. Figure 6A–C illustrates the differential gene expression of up and downregulated genes in response to CAP, TMZ, and CAP + TMZ treatments. Considering a significance level of log2fold change value ≥ ±2.0 and *p*-value ≤ 0.05, our results indicate that 171 genes were upregulated and, 134 genes were downregulated in the CAP alone treatment. In the case of TMZ, 80 genes were upregulated and, 97 genes were downregulated, whereas with the co-treatment, 462 genes were upregulated and 448 genes were downregulated [46,47,48,49]. A list of differentially expressed genes in each treatment condition is shown in Tables S1–S6 in the Supplemental Information. Figure 6A–C illustrates volcano plots of each treatment condition, where red indicates genes that are downregulated, green upregulated, and grey unchanged. We also considered the expression of unaltered genes with a *p*-value less than 0.05 and a non-significant log2fold change denoted by −1 < x < 1 (shown in blue). Based on this, it is apparent that more genes were altered with CAP + TMZ combination treatment as compared to either treatment alone. We next investigated the effect of EM waves and CAP species on the expression of various genes involved in the early response to stress by analyzing early response genes such as EGR1, c-FOS, and FOSL1, an oncogene (c-MYC), oxidative stress response (CAT, GPX1, GPX4, HMOX1, NQO1), and antioxidant-related genes (SOD1, SOD2, NRF2 (NFE2L2)), endoplasmic reticulum stress response genes (HSPs such as HSPA5,6,7, DDIT3) and of P53 and a DNA-damage-inducible protein marker, GADD45A (Figure 6D). We also looked at the *mRNA* expression of key GBM markers by checking genes such as Nestin (NES), PROM1 (CD133), IDH1, and GFAP. We noticed an alteration in the expression of every gene in at least one condition (CAP, TMZ, CAP + TMZ) relative to the untreated control (Figure 6D). The data further suggested a greater effect of CAP + TMZ on these selected pathways in human glioblastoma U87-MG cells relative to CAP or TMZ alone.

### 2.8. Gene Set Enrichment Analysis of Cell Cycle Signaling Pathways in U87-MG Cells after CAP + TMZ Treatment

**Figure 7:**
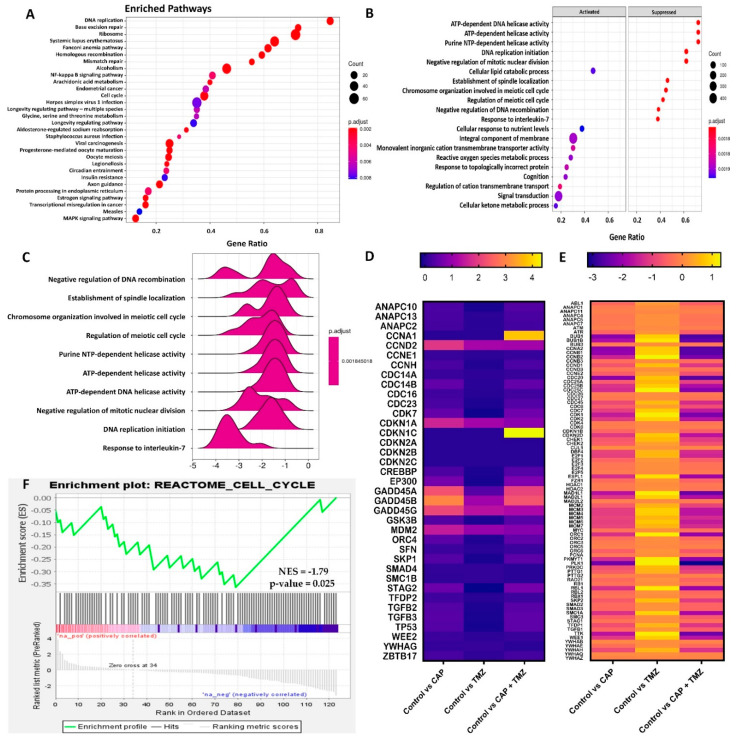

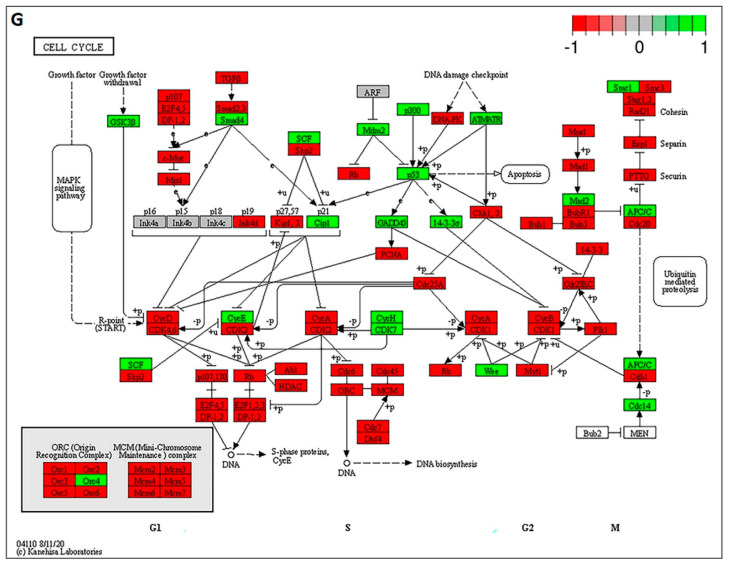
(**A**) Gene set enrichment analysis of pathways enriched in U87-MG cells after treatment with CAP + TMZ. (**B**,**C**) Dot and ridge plots of top 10 altered processes in most enriched pathways. (**D**) Heatmap comparison of upregulated genes (relative to control) of the cell cycle signaling pathway in U87-MG cells post-treatment with CAP, TMZ, or CAP + TMZ. (**E**) Heatmap comparison of downregulated genes of the cell cycle signaling pathway in U87-MG cells post-treatment. (**F**) Gene set enrichment analysis of the cell cycle pathway using the Reactome database resulted in a Normalized Enrichment Score (NES) = −1.79, Nominal *p*-value = 0.025. A negative NES indicated cell cycle gene sets are downregulated and negatively correlated. (**G**) KEGG pathway analysis of cell cycle genes regulated by CAP + TMZ in U87-MG cells.

We next performed gene set enrichment analysis of the top 30 altered pathways, shown in Figure 7A. Pathway enrichment analysis provides a systematic understanding of gene lists involved in a particular disease or treatment. We also looked at the top 10 altered processes from differentially expressed pathways and observed that most of the processes involved in DNA replication, cell growth, and proliferation were suppressed. The subsequent activation of stress response processes such as a response to ROS were noticed (Figure 7B,C). The most enriched pathways were DNA damage, DNA replication, MAPK signaling, and the cell cycle [50,51,52]. To maintain the scope, we focused subsequent analyses on cell-cycle-related pathways. Heatmap profiles of all the genes of cell cycle pathways were studied. Figure 7D shows the top 37 genes were significantly upregulated, whereas Figure 7E shows 87 genes were downregulated in the cell cycle pathway. Cell cycle genes were analyzed using the Gene Set Enrichment Analysis (GSEA) algorithm to evaluate the statistical significance of gene expression using the Reactome pathway database (Figure 7F) [53]. The expression of cell cycle pathway in U87-MG cells using the Reactome database resulted in a Normalized Enrichment Score (NES) = −1.79, a Nominal *p*-value = 0.025, and an FDR q-value = 0.044. A negative NES indicated that genes over-represented in the Reactome cell cycle gene set are downregulated and negatively correlated (blue bar) (Figure 7F).

KEGG pathway analysis was also applied to assess the number of genes up or downregulated in the three distinct phases of the cell cycle (G1, S, and G2/M) (Figure 7G). We searched for genes that were specifically regulated by co-treatment. Highly significant genes were mapped onto the cell cycle and annotated based on KEGG pathway analysis [54]. Based on CAP+TMZ treatment compared to the control, we found that most of the regulatory molecules that determine progression through the cell cycle, such as cyclins and cyclin-dependent kinases, were significantly downregulated post-treatment. This pointed to a significant downregulation of cell cycle processes at the restriction point in the G1 phase at the start of the cell cycle. We also observed a significant underexpression of key S phase genes, suggesting the inhibition of S phase proteins and DNA biosynthesis/replication. A significant over-expression of P53 was noticed when U87-MG cells were treated with CAP + TMZ [55]. We also noted an increase in the expression of the DNA damage markers ATM/ATR and GADD45A [56]. Both of these genes make proteins that help control the rate at which cells grow and divide and are also involved in DNA repair due to external stress. Most of the target genes of the G2/M phase were also downregulated with co-treatment, which corresponds to the reduction in cell viability assays (Figure 1, Figure 2, Figure 3 and Figure 5). Collectively, based on the genome-wide analysis of cell cycle pathways, we can conclude that CAP + TMZ treatment likely induces G2/M cell cycle arrest, potentially leading to apoptosis [12,57].

### 2.9. In Vivo Targeting and Chemotherapy Sensitization of Intracranial GBM Using CAP Jet

**Figure 8:**
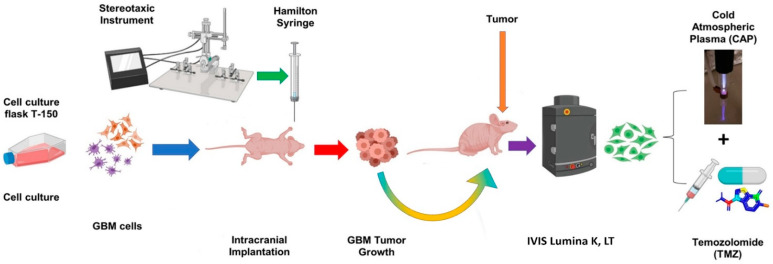
Schematic representation of in vivo CAP targeting plus TMZ treatment of intracranial GBM tumors in nude mice.

**Figure 9:**
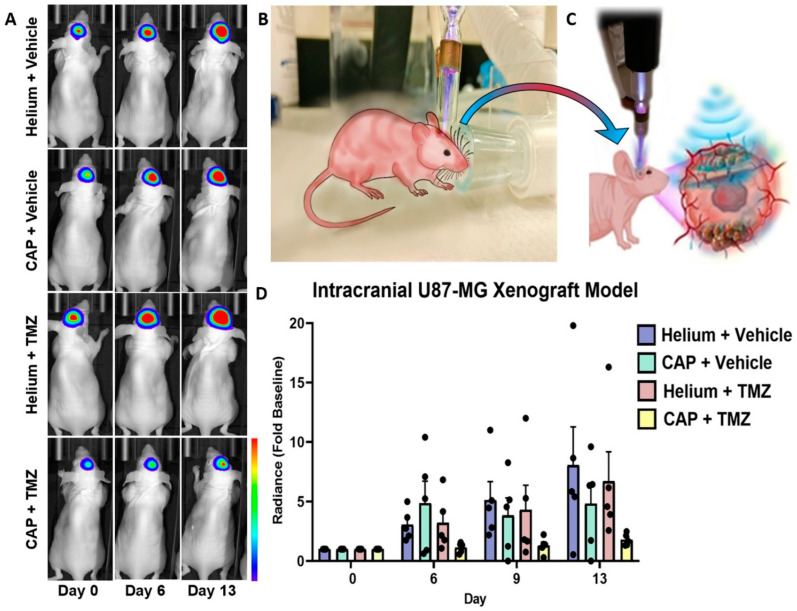
(**A**) Representative bioluminescence images at baseline (day 0) and 6 and 13 days following a single non-invasive CAP treatment with or without daily TMZ or vehicle administration. Helium was used as a CAP control. (**B**) Schematic representation of the non-invasive modality to treat GBM in vivo and (**C**) penetration of EM waves through the skin and skull to target GBM. (**D**) Quantitative summary of radiance (i.e., light emission) over the course of the study. n = 5 per group.

Based on our in vitro findings demonstrating that the CAP jet has the tendency to penetrate bone (Figure 4 and Figure 5), along with a sensitizing effect of CAP on chemotherapy (Figure 2, Figure 3 and Figure 5), we next investigated this in vivo (Figure 8 and Figure 9) [9,58,59,60]. One million U87MG-RedFluc cells were implanted intracranially and allowed to grow for one week. Subsequently, the CAP jet (or helium control) was directly applied to the skull for 1 min at 1 LPM, 12.5 kHz, and 10 V with a fixed distance of 1 cm, as shown in Figure 9B. A schematic of EM wave penetration through the skull to target GBM is illustrated in Figure 9C. This was immediately followed by i.p. administration of TMZ (6 mg/kg/day) or vehicle control 5 days a week for 2 weeks [61,62,63]. No animals died during the course of the experiment (2 weeks). The mice were sacrificed after 2 weeks. The temperature of the skin post-treatment was approximately 25–28 degrees with minor possible skin irritation that healed well, and mice recovered the next day. In vivo bioluminescence imaging revealed a marked and progressive tumor growth over 2 weeks in control animals (helium + vehicle). Helium + TMZ treatment alone did not prevent tumor progression (Figure 9A,D), consistent with the in vitro viability results that showed relative resistance to TMZ treatment. In contrast, relative to the control animals, a single non-invasive CAP treatment resulted in a noticeable inhibition of tumor growth by approximately 40%. Interestingly, a combination of CAP + TMZ virtually prevented GBM progression over the course of the study (Figure 9A,D). Specifically, the bioluminescence average radiance (i.e., light emission) showed an 8.0 ± 3.2-fold increase in tumor volume in the control animals (He–vehicle), 6.7 ± 2.5-fold increase in helium + TMZ, 4.8 ± 1.7-fold increase in CAP + vehicle, and 1.8 ± 0.2-fold increase with co-treatment at day 13. Together, these findings indicate that: (1) the CAP jet can penetrate the skin, bone and can be applied as a non-invasive treatment in vivo, and (2) a single CAP jet treatment enhances the effect of TMZ in the co-treatment and possibly sensitizes GBM tumors to subsequent TMZ administration.

## 3. Discussion

To the best of our knowledge, this is the first study to report the effects of chemotherapy-based potential sensitization via a non-invasive modality of treatment, and the penetration of CAP through the bone/skull of mice in GBM models. GBM is one of the central nervous system’s (CNS) most deadly and difficult-to-treat cancers because of its aggressive nature and location. Surgery is the first-line treatment for GBM patients, but tumor resection brings inherent risks, such as cognitive defects, which can result in reductions in overall survival [1,2,3]. Complete tumor removal by surgery is virtually impossible, and only 10% of individuals with GBM live five years post diagnosis [49]. The poor prognosis in patients with GBM is further due to its resistance to available treatments (e.g., TMZ) [64], which calls for novel methods and therapeutic targets to be identified [65,66,67,68,69,70].

CAP has been used in many applications, including the treatment of cancer, wound healing, dentistry, and sterilization [71,72,73,74,75]. Due to these biomedical applications, CAP has earned considerable credit as an emerging area in the field of science and technology. This has led to an increase in demand for new treatment strategies of diseases in the medical field by CAP, termed ‘plasma medicine’ [76,77]. In the past, many studies involved direct and indirect plasma methods and application via plasma-activated medium or plasma-activated water (or PBS/DMEM), which were shown to be beneficial for in vitro and in vivo treatment of different cancer cells [78,79,80,81]. The treatment of clinical cancers has indeed diversified with the use of CAP, which can potentially induce apoptosis in many types of cancers, including melanoma, pancreatic, ovarian, breast cancer, lung cancer, and GBM [82,83,84,85]. Nevertheless, a challenging part of treating GBM is that it is present in the brain, and any damage to the tissue during the surgery or treatment can cause an array of CNS issues. For example, major neurologic complications include shock, paralysis, tremors, postoperative hematomas, cerebral edema, and seizures [86,87,88]. Therefore, we aimed to create a novel non-invasive CAP jet system to treat GBM in vitro and in vivo and paired this with TMZ co-treatment [9]. The non-invasive nature of this approach presents an opportunity to significantly minimize the risks of damage to tissues surrounding the tumor. There have been multiple combination treatments used with TMZ in several clinical trials for high-grade GBM patients, including radiotherapy and immunotherapy, but these co-treatments are limited due to various resistance factors, including the MGMT gene promoter and the ability of a drug to cross the blood–brain barrier [89,90]. However, the current data demonstrate a potential sensitization effect and the preclinical effectiveness of CAP + TMZ combination in vitro and in vivo to target GBM. Indeed, a single non-invasive CAP treatment in vivo was enough to potentially sensitize intracranial tumors to subsequent TMZ-mediated treatment. Generally, cold atmospheric plasma generates a reactive mix of electrons, ions, excited atoms and molecules, reactive species (e.g., OH, O, H_2_O_2_, O_3_, NO, NO_2_, etc.), UV radiation, and EM waves called the reactive agents (RAs). An interesting aspect of this study was the determination of the delivery, transport, and penetration of these reactive agents through bone. To first characterize the CAP jet device, we found the main content of our jet to be in the range of RONS and reactive agents as mentioned above, and that the spectral region of about 300–490 nm formed peaks due to RNS and peaks at 297 nm; 777 nm indicated ROS. The helium peak was identified at 706.5 nm with a transition state of 1s3s3S => 1s2p3Po (Figure 1A) [24,25,26].

It has been shown that CAP jet can cause DNA damage and apoptosis in cells due to these reactive species and the current in vitro findings support this by demonstrating CAP-induced cell death in all GBM cell lines tested. Similarly, TMZ resulted in reductions in cell viability, although this was more prominent in the TMZ-sensitive A172 and U87-MG cells [91]. However, the combination treatment of CAP + TMZ significantly inhibited the viability of all cell lines (Figure 2, Figure 3 and Figure 5). Collectively, the current results indicate that chemotherapy-induced cytotoxicity can be enhanced with CAP co-treatment. Moreover, they add to existing evidence that has suggested a synergistic effect of TMZ and other agents such as radiotherapy [92]. Combined radiotherapy and TMZ treatment has been shown to be beneficial in newly diagnosed GBM patients. Additional work has also demonstrated a synergistic effect of TMZ in combination with other pharmacological agents [93,94]. However, as discussed above, the efficacy of these combination treatments has remained limited.

While most advances in CAP technology have focused on treatment by direct and indirect methods using a CAP jet and dielectric-barrier discharge (DBD) [80], one of the major challenges in treating GBM is the penetration of RAs. The ability of our novel CAP system to enhance the effect of TMZ is intriguing given that the possible sensitization effect caused by CAP treatment in vitro was evident even though the treated media was immediately removed from the samples. This suggests that the effect was not mediated by the direct ROS/RNS generated by the CAP jet, but instead points to the effect of EM waves and indirect RONS generated by EM waves [95,96]. Indeed, we and others have previously investigated the penetration of EM waves and the effects of tumor-treating fields in GBM even with a barrier [21,97,98]. We confirmed that EM waves can penetrate the bone by measuring the EM wave emission for each well, shown in Figure 4. Measurements with and without the media at varying flow rates and distances indicated that treatment without the media may be more effective. Hence, we considered the morphology of all the cells in Figure 2A,C,E, indicating that there was no external stress caused without media. Building upon this, we performed in vitro experiments and the current findings using human bone as a barrier, implying that non-invasive plasma treatment can penetrate through large bone thicknesses to inhibit cancer cell growth. This is further supported by work demonstrating that EM waves (independent of CAP) can generate ROS/RNS [95,99], and these molecules can act as direct inhibitors of cancer and sensitize GBM to TMZ [100,101]. We also measured direct OES and the penetration of RONS through the bone in a test sample with and without the bone (data not shown). We found no penetration of RONS from direct CAP treatment through the compact bone even after 180 s of treatment, whereas RONS spectra were visualized without bone treatment (Figure 4C). This indicated that GBM inhibition in vivo was a result of EM waves as the primary event in the penetration of the skull of mice leading to a secondary RONS generation shown in our in vitro experiments with the bone [102].

It has been reported that malignant gliomas have alterations in the P53 tumor suppressor gene [103,104], which plays a pivotal role in the cellular response to DNA-damaging agents such as RONS, EM waves, UV light, etc. Therefore, we investigated the role of CAP and EM waves in different genes, including P53, after treating U87-MG human glioma cells. We have already established that CAP generates RONS and various other reactive agents in this and previous studies [3,9,10,12,72,75]. We found out that co-treatment altered most genes in U87-MG cells. CAP + TMZ resulted in alterations (Figure 6D) in oxidative stress response genes (HMOX1, NQO1), antioxidant-related genes (SOD1, SOD2, NRF2), endoplasmic reticulum stress response genes (HSPs like HSPA5,6,7, DDIT3), early response genes (EGR1, c-FOS, and FOSL1) an oncogene (c-MYC), tumor suppressor genes (P53 and GADD45A), and key markers of GBM, i.e., Nestin, PROM1(CD133), IDH1, and GFAP. The upregulation of HMOX1 and NQO1 implies an increase in oxidative stress in the cells. On the contrary, antioxidant-related genes such as superoxide dismutases and NRF2 were also upregulated, indicating that the cells activated an antioxidant mechanism in response to RONS [105,106,107,108]. While these findings lay the framework for avenues of future investigation, overall, the differential gene expression analysis points to many genes that were significantly induced by CAP + TMZ. We also found that most enriched pathways (Figure 7A), after being treated with CAP + TMZ, were related to stress, DNA damage, cell cycle, MAPK signaling, etc. [50,51,52]. Out of these, the top 10 altered processes were mapped based on their significance and are illustrated as dot and ridge plots in Figure 7B,C [53]. After the co-treatment of U87-MG cells, we also found significant alterations in cell cycle signaling pathway genes (Figure 7D–F). KEGG pathway analysis resulted in the downregulation of key markers of the cell cycle (Figure 7G) [54]. As previously described in other studies, cyclin-dependent kinases play important roles in the control of cell division and modulate transcription in response to extra- and intracellular signals/stress [109,110,111]. They were significantly downregulated post-treatment with combination therapy, including the key markers of the G1, S, and G2/M phases. Overall, this indicates that CAP + TMZ likely leads to an increase in cell cycle arrest with a corresponding reduction in cells [12,57].

The location of GBM deep within the brain is further exacerbated by the infiltrative nature of the tumor, which together could limit the systemic delivery of treatments [112]. To overcome this, we developed a CAP jet that can be positioned on the head/skull to treat particular regions without exposing the brain (Figure 9B,C). In line with our in vitro findings with CAP application through bone, we found that non-invasive CAP jet application was successful in partially preventing brain tumor growth (Figure 9A–D). While more work is needed to enhance the efficacy of this approach, and also understand contributing mechanisms, importantly, a single CAP treatment through the skull was effective in sensitizing GBM to subsequent low-dose TMZ therapy. Indeed, TMZ administration by itself was not effective in preventing tumor growth, but when combined with CAP, U87-MG intracranial growth was virtually prevented. The next steps in understanding all these key factors would include varying the TMZ dosage to a lower dose used in this study, increasing plasma effects—because chemotherapy has some side effects—and studying the survival of animals. It would be interesting to also combine plasma with different combinations of drugs such as Doxorubicin, TMZ, Bevacizumab, etc., to introduce a treatment regime for patient-derived xenografts (PDX). We are already working on this approach for future investigations. Indeed, in this study, our results show a good response to the CAP + TMZ treatment just with a single non-invasive therapy by inhibiting the tumor by 78% in the co-treatment. Meanwhile, it is crucial to understand the molecular mechanisms involved in the penetration of RONS through the skin/skull of mice. This will be an interesting study at the molecular level.

Collectively, our findings indicate that CAP can significantly enhance the anti-tumor activity of TMZ in vitro as well as in vivo. Moreover, these results indicate that CAP can be used as a non-invasive mode of treatment in vivo. However, it is still crucial to understand the mechanism of the CAP-mediated transcranial delivery and penetration of RAs and EM waves through bone in vivo.

## 4. Materials and Methods

### 4.1. Experimental Setup

The CAP jet device was designed and manufactured at the Micro-propulsion and Nano-technology Laboratory of the George Washington University, as previously described [3,9,10,12,72,75]. For all experiments, we used the same CAP jet device. The CAP jet was operated at 12.5 kHz for the experiments. The flow rate of helium was maintained at 4 L/min (LPM) for in vitro studies, and we introduced a digital flow meter to control the flow rate for in vivo studies to 1 LPM. The distance between the CAP jet nozzle outlet and the treatment sample media was kept consistent at 3–5 cm. The helium plasma jet was generated via the dielectric barrier discharge. The discharge voltage of the CAP jet was operated at 10 V. A direct method of CAP jet treatment with the cells treated in a culture medium was performed and the CAP sensitization effect was quantified for different time points after the treatment. Graphical Abstract, A represents the circuit diagram and the experimental equipment configuration, and B represents the plasma jet schematic for (i) in vitro and (ii) in vivo intracranial treatment.

### 4.2. Optical Emission Spectroscopy (OES) Spectra Measurement

The OES of the CAP jet was investigated using a SpectraWiz^®^ spectroscopy device. The spectrometer and detection probe were purchased from Stellar Net Inc. (Tampa, FL, USA). The optical probe was placed at a distance of 1 cm in front of the plasma jet nozzle, maintaining a slight distance from the jet so that it did not come into direct contact with the jet. Data were collected with an integration time of 1 s. To elucidate the cocktail of species in the CAP jet, UV–visible–NIR, with a wavelength range of 200–850 nm was investigated. The detection of various RNS and ROS (nitrogen (N_2_), nitric oxide (–NO), nitrogen cation (N_2+_), atomic oxygen (O), and hydroxyl radicals (–OH)) species and reactive agents was performed in the dark [24,25,26].

### 4.3. Chemicals and Drugs

TMZ (Sigma Aldrich, 25 mg) was dissolved in dimethyl sulfoxide (DMSO) or phosphate-buffered saline (PBS) for in vitro and in vivo experiments, respectively. The concentration of DMSO was maintained at 0.25%. The IC50 of the drug was individually calculated for all cell lines, and in vitro treatments were performed by adjusting the TMZ in culture medium to a final concentration of 105, 125, 247, and 400 μM for TMZ in DMEM solution at the time of treatment. For in vivo experiments, TMZ was prepared on each day of injection at 6 mg/kg and then administered intraperitoneally (i.p.).

### 4.4. Cell Lines and Cell Culture

Human GBM cell lines U87-MG, T98G, and A172 were obtained from (American Type Culture Collection, ATCC). U87MG and A172 were cultured in Dulbecco’s Modified Eagle Medium (DMEM, Life Technologies, Washington, WA, USA) supplemented with 10% (*v*/*v*) fetal bovine serum (GE Healthcare, SH30396) and 1% (*v*/*v*) penicillin and streptomycin (Life Technologies). T98G cells were initially maintained in Essential medium containing 10% serum; the base medium for this cell line is ATCC-formulated Eagle’s Minimum Essential Medium, (Catalog No. 30-2003), but they can also be cultured well in DMEM. Cultures were maintained at 37 °C in a humidified incubator containing 5% (*v*/*v*) CO_2_.

For establishing the intracranial xenograft GBM tumor model in vivo, human glioblastoma cancer cells expressing a red-shifted firefly luciferase reporter gene (U87-MG-Red-FLuc, Perkin Elmer, Waltham, MA, USA) were cultured in DMEM and maintained in DMEM medium until the time of implantation [9,60].

### 4.5. Cell Viability Assay Using CAP Jet Direct Treatment In Vitro

All cells were grown to the same passage number (passage 7), and the cells were detached with 0.25% trypsin-EDTA (HyClone) for seeding. The cells were seeded and cultured in a 96-well polystyrene flat-bottom tissue culture-treated microplates (SKU: TC10-096, Stellar Scientific) at a density of 5 × 10^3^ cells per well in 100 μL of media. U87-MG and A172 cells were plated in 100 μL of complete DMEM culture medium, while T98G cells were plated in EMEM complete medium. The cells were seeded in quadruplets for each treatment condition. Cells were incubated for 24 h to ensure proper cell adherence and stability. On day 2, the media was aspirated from the wells, the cells were washed with PBS once to remove dead cells, and fresh media was added to the wells. The cells were treated by He CAP for 0, 30, 60, and 90 s. Post treatment, the cells were further incubated at 37 °C for 72 h. An MTT assay was performed to assess cell viability, as described below.

The cell viability of all glioblastoma cancer cell lines was measured with an MTT (3-(4,5-Dimethyl-2-thiazol)-2,5-Diphenyl-2H-tetrazolium Bromide) assay following standard protocols provided by the manufacturer (Sigma-Aldrich, M2128). A volume of 100 μL of MTT solution was added to each well followed by a 3 h incubation at 37 °C in a humidified incubator containing 5% (*v*/*v*) CO_2_. The MTT solution was removed after the incubation, and 100 μL of MTT solvent (0.4% (*v*/*v*) HCl in anhydrous isopropanol) was then added per well and mixed by pipetting gently. The reading was taken after 10 min or within half an hour. The plate was read by a Synergy H1 hybrid multi-mode microplate reader after gently shaking for 30 s in a linear mode, and the absorbance was recorded at 570 nm.

### 4.6. CAP Jet In Vitro Sensitization of GBM to TMZ

For sensitization experiments, we performed a 5-day treatment similar to our in vivo treatments mentioned below. Here, we seeded 5000 cells per well in a 96-well plate and treated the cells with CAP. We optimized the time points to 0, 30, and 60 s for this study, and after the CAP jet treatment, we removed the media containing reactive agents immediately and replaced it with fresh DMEM/EMEM media for the untreated control and CAP treatment or 105, 125, and 247 μM TMZ for the CAP + TMZ combination treatment based on the IC50 for each cell line. The plate was incubated for 24 h at 37 °C in a humidified incubator containing 5% (*v*/*v*) CO_2_. On day 2, the media and TMZ was discarded and replaced with fresh media and again treated with the CAP jet. After treatment, the media with reactive agents was removed and replaced with fresh media and TMZ. This process was repeated for 5 days. After the 5th day of the treatment, the cells were further incubated at 37 °C for 72 h. An MTT assay was performed to assess cell viability, as described above.

### 4.7. Bone as a Barrier for In Vitro CAP Jet Treatment

Human fibula bone was obtained from the Bone Room (Los Alamitos, CA, USA) and was cut into small pieces using a saw. The bone pieces were fined and shaped to fit the diameter and circumference of 96-well plates. The thickness and width of the bone were also kept in mind and were fined to match the skull of mice [113,114]. We used a bone width of 1.35 mm. The EM wave includes three frequency components. The one at the discharge frequency and plasma frequency depends on the electron density, while the intensities of UV–Vis emission depend on the densities of excited species, which are mainly generated by the electron density and electron temperature. EM wave emission was measured to analyze the penetration of EM waves through the bone by attaching an electrode beneath each well. These electrodes thus received extra electric potential peaks when the streamer approached the bone, shown in Figure 5. All cells were seeded and cultured in 96-well microplates (SKU: TC10-096, Stellar Scientific) at a density of 5 × 10^3^ cells per well in 100 μL of media. As previously mentioned, U87-MG and A172 cells were plated in 100 μL of complete DMEM culture medium, while T98G was plated in EMEM complete medium in quadruplets, and the plates were incubated for 24 h to ensure proper cell adherence and stability. On day 2, the media was aspirated from the plates, the cells were washed with PBS once to remove dead cells, and fresh media was added to the wells. The bone was sterilized with 70% ethanol and placed in UV for 20 min before treating the wells and was placed onto the wells to act as a barrier. The cells were treated without the media and bone on top as a barrier. The cells were treated by He CAP for 0, 30, and 60 s. Post treatment, the cells were further incubated at 37 °C for 72 h. An MTT assay was performed to assess cell viability as described above.

### 4.8. RNA-Seq Analysis and Differential Gene Expression of U87-MG Cells after Co-Treatment

U87-MG cells were grown in triplicate in a 6-well plate at a density of 3 × 10^5^. They were harvested, and RNA was extracted using the standard RNeasy mini prep kit (Qiagen). The RNA samples were checked for quality and quantity using a nanodrop 2000 (ThermoFisher Scientific, Waltham, MA, USA). The library for sequencing was prepared using the TruSeq^®^ Stranded Total RNA sample preparation kit as per the manufacturer’s guidelines and 500 ng of RNA was used for each treatment condition. RNA sequencing was performed with Illumina NextSeq-500 sequencing platform (Illumina) using paired-end runs. Galaxy server was used to preprocess the raw sequencing data, including trimming the adapter sequences, filtering artifacts, and discarding low-quality reads with a lower quality score using fastqc and trimmomatic. The sequences were merged together and assembled using stringtie. The reads were aligned to the human reference genome (hg38) using Hisat2 and differential gene expression testing was performed using DeSeq2 with the default options [115]. The genes were then annotated using the functional annotation tool. Genome-wide analysis of the target genes of CAP and TMZ were performed and a comparison of the heatmap profiles was carried out.

The Gene Set Enrichment Analysis (GSEA) v4.1.0 (Broad Institute, Cambridge, MA, USA) algorithm was used to evaluate the statistical significance of gene expression via the Reactome pathway database. Gene Set Enrichment Analysis is supported by the Broad Institute (https://www.gsea-msigdb.org/gsea/index.jsp, accessed on 21 July 2021) [53]. GSEA yielded the expression of genes and enriched pathways in U87-MG cells, out of which, the top 30 enriched signaling pathways, top 10 enriched processes, and cell cycle pathways were studied. The Kyoto Encyclopedia of Genes and Genomes pathway analysis of DEGs showed a negative regulation of key markers of the cell cycle [54].

### 4.9. In Vivo Treatment of Intracranial Glioblastoma Xenograft Model Using CAP Jet

All animal protocols were approved by the George Washington University’s Institutional Animal Care and Use Committee (IACUC). Eight-week-old (56 days) female athymic nude mice (Charles River, NU(NCr)-Foxn1nu) were anesthetized i.p. (Ketamine 100 mg/kg mixed with Xylazine 10 mg/kg) and administered surgical analgesic (Ketofen 100 mg/kg). Mice were then placed in a stereotaxic frame and the skull was leveled between the bregma and lambda. A small hole was drilled at the desired location and 1 × 10^6^ U87MG-RedFluc cells in 2 μL of DMEM were injected into the frontal lobe based on the following coordinates (relative to Bregma) using a Hamilton syringe: 2.2 mm ventral from the dorsal surface of the skull, 1.0 mm caudal, and 2.0 mm lateral. Mice were provided with 7 days of recovery and baseline bioluminescence imaging was then performed. Specifically, mice were anesthetized with isoflurane and the substrate luciferin (150 mg/kg) was administered i.p. Mice were then transferred to an imaging system (IVIS Lumina K, Perkin Elmer) and positioned in a nose cone to maintain the anesthesia state. In vivo bioluminescent imaging was performed 10 min post-substrate injection using a charge-coupled camera system cooled to −80 °C to achieve maximum sensitivity. The exposure time used was 2 s with medium binning 2, F/stop = 1, and EM gain off. Following baseline image collection, the He CAP jet was applied over the surface of the skin/skull for 1 min at a 1 LPM flow rate. The CAP jet was operated at 12.5 kHz frequency and 10 V discharge voltage. The distance between the plasma jet and skull/head during the treatment was kept constant at ~1 cm. TMZ (6 mg/kg) was then administered (i.p.) for the treatment groups [61,62,63]. Mice were administered TMZ 5 days a week for 2 weeks, and weekly imaging was also performed. Images were analyzed using built-in software by placing a region of interest over the head of each animal. The region of interest size was identical across animals and radiance values were normalized to baseline light emission.

### 4.10. Definition of Control, Vehicle

There are “0” and “control” treatments in Figure 1, Figure 2, Figure 3, Figure 4 and Figure 5, which represent that no TMZ or CAP was performed.

In Figure 9, “vehicle” represents the control for the drug TMZ. The vehicle consisted of DMSO + PBS in this study.

### 4.11. Statistical Analysis

For all in vitro assays, at least three experiments were performed with biological quadruplets. Data were plotted using Prism 8.4.3 (GraphPad Software, San Diego, CA, USA) and are presented as the mean ± standard error. Statistical analysis was performed using a paired Student’s t-test, as indicated, or one-way or two-way analysis of variances (ANOVA), as indicated. Follow-up tests were performed using Dunnett’s or Tukey’s multiple comparison post hoc test. The level of significance was denoted as follows: * *p* < 0.05; ** *p* < 0.01; and *** *p* < 0.001; **** *p* < 0.0001, ns—not significant.

## 5. Conclusions

In conclusion, in vitro and in vivo findings demonstrate that CAP jet application enhanced the cytotoxicity of TMZ in GBM cells. We also confirmed the effect of indirect RONS and the ability of EM waves to penetrate the skin and bone as a significant reduction in tumor size was noted with non-invasive CAP treatment in vivo and cell death in vitro following CAP treatment through bone. Furthermore, even with non-invasive treatment, CAP amplified the effect of TMZ, making the GBM cells more responsive to chemotherapy treatment. We also confirmed this by studying the effects of CAP + TMZ at the molecular level and RNA-seq data revealed differentially expressed genes involved in the cell cycle pathway to be underexpressed, causing cell cycle arrest. The effects of certain genes regulated by EM waves were noted. Overall, these findings point to a new non-invasive cancer treatment modality and indicate that CAP can be successfully used to potentially sensitize GBM to low doses of TMZ, thus providing a means to reduce chemotherapy cytotoxicity and non-invasively treat GBM.

## Data Availability

The data presented in this study that support the findings are available on reasonable request from the corresponding author.

## Supplementary Materials

The following are available online at https://www.mdpi.com/article/10.3390/cancers13174485/s1, Table S1: list of top 171 genes upregulated in CAP treatment, Table S2: list of top 134 genes downregulated in CAP treatment, Table S3: list of top 80 genes upregulated in TMZ treatment, Table S4: list of top 97 genes downregulated in TMZ treatment, Table S5: list of top 462 genes upregulated in CAP + TMZ treatment, and Table S6: list of top 448 genes downregulated in CAP + TMZ treatment.
