# Supplementary material for: In Vitro and In Vivo Enhancement of Temozolomide Effect in Human Glioblastoma by Non-Invasive Application of Cold Atmospheric Plasma
Source: Cancers (Basel). 2021 Sep 6;13(17):4485. doi: 10.3390/cancers13174485 (PMC8430547; doi:10.3390/cancers13174485)
Supplement: Supplementary file 1 [file cancers-13-04485-s001.zip › SI Table S1 - Top upregulated genes in CAP treatment.pdf]

Supplementary Table 1: List of top 171 up-regulated genes in CAP treatment

Sr. No	Gene Name	GeneID	Log2(FC)	P-adj	Description	Chromosome	Strand
[1]	HSPA6	ENSG00000173110.8	6.785705	3.75E-274	heat shock protein family A (Hsp70) member 6 [Source:HGNC Symbol;Acc:HGNC:5239]	chr1	+
[2]	SMG1P2	ENSG00000205534.6	6.438267	5.14E-64	SMG1 pseudogene 2 [Source:HGNC Symbol;Acc:HGNC:49859]	chr16	-
[3]	HSPA7	ENSG00000225217.1	5.865503	7.84E-101	heat shock protein family A (Hsp70) member 7 (pseudogene) [Source:HGNC Symbol;Acc:HGNC:5240]	chr1	+
[4]	AL671762.1	ENSG00000285565.1	5.843196	6.10E-49	novel transcript	chr6	+
[5]	HSPA8P8	ENSG00000229091.3	5.597482	6.87E-95	heat shock protein family A (Hsp70) member 8 pseudogene 8 [Source:HGNC Symbol;Acc:HGNC:44923]	chr7	+
[6]	RASD1	ENSG00000108551.5	5.209623	1.06E-63	ras related dexamethasone induced 1 [Source:HGNC Symbol;Acc:HGNC:15828]	chr17	-
[7]	UBE2F-SCLY	ENSG00000258984.5	4.781227	2.26E-31	UBE2F-SCLY readthrough (NMD candidate) [Source:HGNC Symbol;Acc:HGNC:48339]	chr2	+
[8]	SNAI1	ENSG00000124216.4	4.486268	7.86E-57	snail family transcriptional repressor 1 [Source:HGNC Symbol;Acc:HGNC:11128]	chr20	+
[9]	ANKRD1	ENSG00000148677.6	4.277808	2.10E-36	ankyrin repeat domain 1 [Source:HGNC Symbol;Acc:HGNC:15819]	chr10	-
[10]	XIRP1	ENSG00000168334.9	4.002498	2.98E-18	xin actin binding repeat containing 1 [Source:HGNC Symbol;Acc:HGNC:14301]	chr3	-
[11]	TEX14	ENSG00000121101.15	3.608406	3.21E-14	testis expressed 14, intercellular bridge forming factor [Source:HGNC Symbol;Acc:HGNC:11737]	chr17	-
[12]	TCTE1	ENSG00000146221.10	3.586367	3.78E-14	t-complex-associated-testis-expressed 1 [Source:HGNC Symbol;Acc:HGNC:11693]	chr6	-
[13]	FAM166A	ENSG00000188163.8	3.54554	4.45E-30	family with sequence similarity 166 member A [Source:HGNC Symbol;Acc:HGNC:33818]	chr9	-

[14]	FRG2B	ENSG00000225899.8	3.521867	3.69E-16	FSHD region gene 2 family member B [Source:HGNC Symbol;Acc:HGNC:33518]	chr10	-
[15]	PYGM	ENSG00000068976.14	3.504451	2.13E-13	glycogen phosphorylase, muscle associated [Source:HGNC Symbol;Acc:HGNC:9726]	chr11	-
[16]	FOSB	ENSG00000125740.14	3.471092	1.06E-163	FosB proto-oncogene, AP-1 transcription factor subunit [Source:HGNC Symbol;Acc:HGNC:3797]	chr19	+
[17]	FOS	ENSG00000170345.10	3.45061	3.67E-195	Fos proto-oncogene, AP-1 transcription factor subunit [Source:HGNC Symbol;Acc:HGNC:3796]	chr14	+
[18]	GDF15	ENSG00000130513.6	3.341701	0	growth differentiation factor 15 [Source:HGNC Symbol;Acc:HGNC:30142]	chr19	+
[19]	CHAC1	ENSG00000128965.13	3.332273	1.64E-75	ChaC glutathione specific gamma-glutamylcyclotransferase 1 [Source:HGNC Symbol;Acc:HGNC:28680]	chr15	+
[20]	TRIB3	ENSG00000101255.11	3.27466	2.86E-259	tribbles pseudokinase 3 [Source:HGNC Symbol;Acc:HGNC:16228]	chr20	+
[21]	CDH16	ENSG00000166589.13	3.259462	2.31E-11	cadherin 16 [Source:HGNC Symbol;Acc:HGNC:1755]	chr16	-
[22]	RGS16	ENSG00000143333.7	3.257436	2.42E-15	regulator of G protein signaling 16 [Source:HGNC Symbol;Acc:HGNC:9997]	chr1	-
[23]	FNDC7	ENSG00000143107.10	3.16889	8.49E-14	fibronectin type III domain containing 7 [Source:HGNC Symbol;Acc:HGNC:26668]	chr1	+
[24]	SEMA3G	ENSG00000010319.7	3.149107	1.40E-10	semaphorin 3G [Source:HGNC Symbol;Acc:HGNC:30400]	chr3	-
[25]	KCNB1	ENSG00000158445.10	3.123574	1.39E-14	potassium voltage-gated channel subfamily B member 1 [Source:HGNC Symbol;Acc:HGNC:6231]	chr20	-
[26]	HRNR	ENSG00000197915.6	3.114236	1.21E-208	hornerin [Source:HGNC Symbol;Acc:HGNC:20846]	chr1	-
[27]	AOC3	ENSG00000131471.7	3.093482	1.10E-19	amine oxidase copper containing 3 [Source:HGNC Symbol;Acc:HGNC:550]	chr17	+

[28]	BTG2	ENSG00000159388.6	3.075122	1.59E-34	BTG anti-proliferation factor 2 [Source:HGNC Symbol;Acc:HGNC:1131]	chr1	+
[29]	GADD45B	ENSG00000099860.9	3.004451	0	growth arrest and DNA damage inducible beta [Source:HGNC Symbol;Acc:HGNC:4096]	chr19	+
[30]	ULBP1	ENSG00000111981.5	2.996039	2.62E-111	UL16 binding protein 1 [Source:HGNC Symbol;Acc:HGNC:14893]	chr6	+
[31]	CRYAB	ENSG00000109846.9	2.991635	7.17E-12	crystallin alpha B [Source:HGNC Symbol;Acc:HGNC:2389]	chr11	-
[32]	TRIM54	ENSG00000138100.13	2.972804	3.03E-12	tripartite motif containing 54 [Source:HGNC Symbol;Acc:HGNC:16008]	chr2	+
[33]	LINC01647	ENSG00000235643.2	2.890033	8.34E-09	long intergenic non-protein coding RNA 1647 [Source:HGNC Symbol;Acc:HGNC:52434]	chr1	+
[34]	ADM2	ENSG00000128165.9	2.861327	3.33E-11	adrenomedullin 2 [Source:HGNC Symbol;Acc:HGNC:28898]	chr22	+
[35]	SLC7A11	ENSG00000151012.13	2.846124	0	solute carrier family 7 member 11 [Source:HGNC Symbol;Acc:HGNC:11059]	chr4	-
[36]	CYP4F11	ENSG00000171903.16	2.844703	1.78E-09	cytochrome P450 family 4 subfamily F member 11 [Source:HGNC Symbol;Acc:HGNC:13265]	chr19	-
[37]	WDR49	ENSG00000174776.11	2.835377	1.65E-08	WD repeat domain 49 [Source:HGNC Symbol;Acc:HGNC:26587]	chr3	-
[38]	CD79A	ENSG00000105369.9	2.79531	3.01E-08	CD79a molecule [Source:HGNC Symbol;Acc:HGNC:1698]	chr19	+
[39]	MORN3	ENSG00000139714.12	2.768175	4.12E-08	MORN repeat containing 3 [Source:HGNC Symbol;Acc:HGNC:29807]	chr12	-
[40]	ZCCHC12	ENSG00000174460.4	2.761703	4.51E-08	zinc finger CCHC-type containing 12 [Source:HGNC Symbol;Acc:HGNC:27273]	chrX	+
[41]	AC116317.1	ENSG00000286176.2	2.757175	5.97E-08	novel transcript	chr4	-
[42]	PCDH10	ENSG00000138650.9	2.74145	9.23E-48	protocadherin 10 [Source:HGNC Symbol;Acc:HGNC:13404]	chr4	+

[43]	VLDLR-AS1	ENSG00000236404.10	2.739858	1.38E-08	VLDLR antisense RNA 1 [Source:HGNC Symbol;Acc:HGNC:49621]	chr9	-
[44]	NR4A2	ENSG00000153234.14	2.726517	2.68E-22	nuclear receptor subfamily 4 group A member 2 [Source:HGNC Symbol;Acc:HGNC:7981]	chr2	-
[45]	C1orf116	ENSG00000182795.13	2.725826	1.71E-08	chromosome 1 open reading frame 116 [Source:HGNC Symbol;Acc:HGNC:28667]	chr1	-
[46]	AC004988.1	ENSG00000203446.2	2.721426	4.96E-33	SUGCT antisense RNA 1 [Source:HGNC Symbol;Acc:HGNC:40745]	chr7	-
[47]	ATF3	ENSG00000162772.17	2.709543	4.34E-176	activating transcription factor 3 [Source:HGNC Symbol;Acc:HGNC:785]	chr1	+
[48]	MYO16	ENSG00000041515.16	2.703252	1.82E-08	myosin XVI [Source:HGNC Symbol;Acc:HGNC:29822]	chr13	+
[49]	AOC2	ENSG00000131480.9	2.701219	3.83E-24	amine oxidase copper containing 2 [Source:HGNC Symbol;Acc:HGNC:549]	chr17	+
[50]	CDC42BPG	ENSG00000171219.9	2.689907	4.92E-08	CDC42 binding protein kinase gamma [Source:HGNC Symbol;Acc:HGNC:29829]	chr11	-
[51]	SESN2	ENSG00000130766.5	2.684871	6.12E-287	sestrin 2 [Source:HGNC Symbol;Acc:HGNC:20746]	chr1	+
[52]	RND1	ENSG00000172602.11	2.676245	6.16E-11	Rho family GTPase 1 [Source:HGNC Symbol;Acc:HGNC:18314]	chr12	-
[53]	RNF103-CHMP3	ENSG00000249884.8	2.673563	2.32E-09	RNF103-CHMP3 readthrough [Source:HGNC Symbol;Acc:HGNC:38847]	chr2	-
[54]	TRIM69	ENSG00000185880.13	2.660738	1.67E-07	tripartite motif containing 69 [Source:HGNC Symbol;Acc:HGNC:17857]	chr15	+
[55]	CTH	ENSG00000116761.12	2.637362	2.48E-74	cystathionine gamma-lyase [Source:HGNC Symbol;Acc:HGNC:2501]	chr1	+
[56]	KLHL30-AS1	ENSG00000279484.1	2.636637	9.60E-12	KLHL30 antisense RNA 1 [Source:HGNC Symbol;Acc:HGNC:31018]	chr2	-
[57]	IGFL2-AS1	ENSG00000268621.6	2.632185	5.17E-10	IGFL2 antisense RNA 1 [Source:HGNC Symbol;Acc:HGNC:52559]	chr19	-
[58]	FAM71E2	ENSG00000180043.11	2.595935	1.90E-07	family with sequence similarity 71 member E2 [Source:HGNC Symbol;Acc:HGNC:25278]	chr19	-

[59]	MRC1	ENSG00000260314.3	2.595682	4.76E-08	mannose receptor C-type 1 [Source:HGNC Symbol;Acc:HGNC:7228]	chr10	+
[60]	MYO15B	ENSG00000266714.9	2.578392	2.13E-08	myosin XVB [Source:HGNC Symbol;Acc:HGNC:14083]	chr17	+
[61]	RGS2	ENSG00000116741.8	2.558029	1.26E-53	regulator of G protein signaling 2 [Source:HGNC Symbol;Acc:HGNC:9998]	chr1	+
[62]	MMP25	ENSG00000008516.17	2.549562	3.26E-07	matrix metallopeptidase 25 [Source:HGNC Symbol;Acc:HGNC:14246]	chr16	+
[63]	TRIM72	ENSG00000177238.14	2.544406	1.84E-07	tripartite motif containing 72 [Source:HGNC Symbol;Acc:HGNC:32671]	chr16	+
[64]	SERPINB10	ENSG00000242550.6	2.530519	3.91E-07	serpin family B member 10 [Source:HGNC Symbol;Acc:HGNC:8942]	chr18	+
[65]	AC005532.1	ENSG00000230825.2	2.512786	9.59E-07	novel transcript	chr7	+
[66]	DDIT4	ENSG00000168209.5	2.510242	0	DNA damage inducible transcript 4 [Source:HGNC Symbol;Acc:HGNC:24944]	chr10	+
[67]	AC009336.2	ENSG00000279160.1	2.5036	3.33E-07	TEC	chr2	-
[68]	FUT1	ENSG00000174951.11	2.488023	9.59E-07	fucosyltransferase 1 (H blood group) [Source:HGNC Symbol;Acc:HGNC:4012]	chr19	-
[69]	LSMEM1	ENSG00000181016.9	2.478281	8.38E-10	leucine rich single-pass membrane protein 1 [Source:HGNC Symbol;Acc:HGNC:22036]	chr7	+
[70]	FP236383.1	ENSG00000274868.1	2.441704	1.14E-06	nan	chr21	+
[71]	FP671120.1	ENSG00000275664.1	2.441704	1.14E-06	nan	chr21	+
[72]	FP671120.3	ENSG00000277671.1	2.441704	1.14E-06	nan	chr21	+
[73]	FP236383.2	ENSG00000278775.1	2.441704	1.14E-06	nan	chr21	+
[74]	AC068506.1	ENSG00000278464.2	2.43209	1.31E-06	novel transcript	chr18	-
[75]	PKD1L1	ENSG00000158683.8	2.427537	2.59E-06	polycystin 1 like 1, transient receptor potential channel interacting [Source:HGNC Symbol;Acc:HGNC:18053]	chr7	-
[76]	TRIM55	ENSG00000147573.17	2.405025	3.27E-06	tripartite motif containing 55 [Source:HGNC Symbol;Acc:HGNC:14215]	chr8	+
[77]	ARC	ENSG00000198576.4	2.396054	1.46E-233	activity regulated cytoskeleton associated protein [Source:HGNC Symbol;Acc:HGNC:648]	chr8	-

[78]	RADIL	ENSG00000157927.17	2.391756	6.36E-08	Rap associating with DIL domain [Source:HGNC Symbol;Acc:HGNC:22226]	chr7	-
[79]	ARHGAP9	ENSG00000123329.19	2.391134	3.69E-06	Rho GTPase activating protein 9 [Source:HGNC Symbol;Acc:HGNC:14130]	chr12	-
[80]	TMPRSS11F	ENSG00000198092.6	2.385111	1.12E-07	transmembrane serine protease 11F [Source:HGNC Symbol;Acc:HGNC:29994]	chr4	-
[81]	TENT5C	ENSG00000183508.5	2.383051	8.70E-27	terminal nucleotidyltransferase 5C [Source:HGNC Symbol;Acc:HGNC:24712]	chr1	+
[82]	CMYA5	ENSG00000164309.15	2.377949	4.25E-18	cardiomyopathy associated 5 [Source:HGNC Symbol;Acc:HGNC:14305]	chr5	+
[83]	BMF	ENSG00000104081.14	2.371261	6.41E-24	Bcl2 modifying factor [Source:HGNC Symbol;Acc:HGNC:24132]	chr15	-
[84]	UNC13D	ENSG00000092929.11	2.363321	6.47E-07	unc-13 homoLog D [Source:HGNC Symbol;Acc:HGNC:23147]	chr17	-
[85]	PIWIL2	ENSG00000197181.12	2.34879	3.74E-06	piwi like RNA-mediated gene silencing 2 [Source:HGNC Symbol;Acc:HGNC:17644]	chr8	+
[86]	IL17REL	ENSG00000188263.10	2.345556	5.96E-06	interleukin 17 receptor E like [Source:HGNC Symbol;Acc:HGNC:33808]	chr22	-
[87]	TBR1	ENSG00000136535.15	2.341482	6.21E-06	T-box brain transcription factor 1 [Source:HGNC Symbol;Acc:HGNC:11590]	chr2	+
[88]	MUC17	ENSG00000169876.14	2.337255	6.46E-06	mucin 17, cell surface associated [Source:HGNC Symbol;Acc:HGNC:16800]	chr7	+
[89]	CSPG5	ENSG00000114646.10	2.336411	6.51E-06	chondroitin sulfate proteoglycan 5 [Source:HGNC Symbol;Acc:HGNC:2467]	chr3	-
[90]	KLHL30	ENSG00000168427.9	2.336367	1.23E-06	kelch like family member 30 [Source:HGNC Symbol;Acc:HGNC:24770]	chr2	+
[91]	TTC24	ENSG00000187862.12	2.33036	6.94E-06	tetratricopeptide repeat domain 24 [Source:HGNC Symbol;Acc:HGNC:32348]	chr1	+
[92]	LINC02009	ENSG00000283646.2	2.319849	7.61E-08	long intergenic non-protein coding RNA 2009 [Source:HGNC Symbol;Acc:HGNC:52845]	chr3	-

[93]	NMRAL2P	ENSG00000171658.8	2.318376	4.20E-09	NmrA like redox sensor 2, pseudogene [Source:HGNC Symbol;Acc:HGNC:52332]	chr3	+
[94]	RGMA	ENSG00000182175.14	2.316319	1.98E-06	repulsive guidance molecule BMP co-receptor a [Source:HGNC Symbol;Acc:HGNC:30308]	chr15	-
[95]	PLK2	ENSG00000145632.15	2.301251	0	polo like kinase 2 [Source:HGNC Symbol;Acc:HGNC:19699]	chr5	-
[96]	TINCR	ENSG00000223573.7	2.298154	8.43E-06	TINCR ubiquitin domain containing [Source:HGNC Symbol;Acc:HGNC:14607]	chr19	-
[97]	CCDC62	ENSG00000130783.14	2.293842	2.36E-07	coiled-coil domain containing 62 [Source:HGNC Symbol;Acc:HGNC:30723]	chr12	+
[98]	AC010336.1	ENSG00000214248.2	2.286967	1.07E-05	novel transcript, antisense to LRRC8E	chr19	-
[99]	SPINK1	ENSG00000164266.10	2.268021	1.86E-24	serine peptidase inhibitor Kazal type 1 [Source:HGNC Symbol;Acc:HGNC:11244]	chr5	-
[100]	AC108517.2	ENSG00000286097.1	2.26795	3.80E-06	novel transcript	chr4	-
[101]	OVGP1	ENSG00000085465.13	2.262879	2.46E-06	oviductal glycoprotein 1 [Source:HGNC Symbol;Acc:HGNC:8524]	chr1	-
[102]	CREBRF	ENSG00000164463.12	2.262448	7.96E-114	CREB3 regulatory factor [Source:HGNC Symbol;Acc:HGNC:24050]	chr5	+
[103]	FLG-AS1	ENSG00000237975.7	2.257098	6.58E-11	FLG antisense RNA 1 [Source:HGNC Symbol;Acc:HGNC:27913]	chr1	+
[104]	MSC	ENSG00000178860.8	2.25523	1.68E-112	musculin [Source:HGNC Symbol;Acc:HGNC:7321]	chr8	-
[105]	GADD45A	ENSG00000116717.13	2.253486	2.60E-271	growth arrest and DNA damage inducible alpha [Source:HGNC Symbol;Acc:HGNC:4095]	chr1	+
[106]	SLIT2	ENSG00000145147.20	2.245738	3.99E-27	slit guidance ligand 2 [Source:HGNC Symbol;Acc:HGNC:11086]	chr4	+
[107]	PMAIP1	ENSG00000141682.11	2.245264	1.16E-198	phorbol-12-myristate-13-acetate-induced protein 1 [Source:HGNC Symbol;Acc:HGNC:9108]	chr18	+
[108]	INHBE	ENSG00000139269.3	2.244313	1.72E-08	inhibin subunit beta E [Source:HGNC Symbol;Acc:HGNC:24029]	chr12	+

[109]	VWF	ENSG00000110799.13	2.237611	3.17E-06	von Willebrand factor [Source:HGNC Symbol;Acc:HGNC:12726]	chr12	-
[110]	STX17-AS1	ENSG00000255145.3	2.235405	1.77E-05	STX17 antisense RNA 1 [Source:HGNC Symbol;Acc:HGNC:51174]	chr9	-
[111]	ADGRG3	ENSG00000182885.17	2.228503	1.45E-05	adhesion G protein-coupled receptor G3 [Source:HGNC Symbol;Acc:HGNC:13728]	chr16	+
[112]	COL20A1	ENSG00000101203.16	2.226244	1.45E-05	collagen type XX alpha 1 chain [Source:HGNC Symbol;Acc:HGNC:14670]	chr20	+
[113]	HSPA1A	ENSG00000204389.10	2.212583	4.32E-68	heat shock protein family A (Hsp70) member 1A [Source:HGNC Symbol;Acc:HGNC:5232]	chr6	+
[114]	SLC7A11-AS1	ENSG00000250033.5	2.209068	6.75E-09	SLC7A11 antisense RNA 1 [Source:HGNC Symbol;Acc:HGNC:44064]	chr4	+
[115]	C5AR1	ENSG00000197405.8	2.198236	1.34E-20	complement C5a receptor 1 [Source:HGNC Symbol;Acc:HGNC:1338]	chr19	+
[116]	LRRC66	ENSG00000188993.3	2.192187	2.67E-05	leucine rich repeat containing 66 [Source:HGNC Symbol;Acc:HGNC:34299]	chr4	-
[117]	PTGER3	ENSG00000050628.20	2.189278	1.33E-06	prostaglandin E receptor 3 [Source:HGNC Symbol;Acc:HGNC:9595]	chr1	-
[118]	ATP1B2	ENSG00000129244.9	2.182853	8.26E-06	ATPase Na+/K+ transporting subunit beta 2 [Source:HGNC Symbol;Acc:HGNC:805]	chr17	+
[119]	GPD1	ENSG00000167588.13	2.176708	3.06E-05	glycerol-3-phosphate dehydrogenase 1 [Source:HGNC Symbol;Acc:HGNC:4455]	chr12	+
[120]	AL355916.2	ENSG00000258926.2	2.176231	3.08E-05	novel transcript, antisense to PRKCH	chr14	-
[121]	AC087521.1	ENSG00000244953.1	2.171182	3.24E-05	novel transcript	chr11	-
[122]	AC105046.1	ENSG00000261026.1	2.164324	3.44E-05	novel transcript, overlapping to EGR3	chr8	-
[123]	AC017104.2	ENSG00000230047.1	2.157895	3.50E-05	HIGD2A pseudogene 1 [Source:HGNC Symbol;Acc:HGNC:54793]	chr2	-
[124]	TP53INP1	ENSG00000164938.14	2.15744	9.20E-260	tumor protein p53 inducible nuclear protein 1 [Source:HGNC Symbol;Acc:HGNC:18022]	chr8	-

[125]	CRISPLD2	ENSG00000103196.12	2.153065	1.79E-10	cysteine rich secretory protein LCCL domain containing 2 [Source:HGNC Symbol;Acc:HGNC:25248]	chr16	+
[126]	DDIT3	ENSG00000175197.12	2.150635	4.64E-242	DNA damage inducible transcript 3 [Source:HGNC Symbol;Acc:HGNC:2726]	chr12	-
[127]	KLF4	ENSG00000136826.15	2.145219	4.15E-41	Kruppel like factor 4 [Source:HGNC Symbol;Acc:HGNC:6348]	chr9	-
[128]	AC099329.2	ENSG00000273328.5	2.138803	3.65E-05	novel transcript	chr3	+
[129]	KLHL24	ENSG00000114796.16	2.138262	1.31E-131	kelch like family member 24 [Source:HGNC Symbol;Acc:HGNC:25947]	chr3	+
[130]	TMEM184A	ENSG00000164855.16	2.132881	1.77E-05	transmembrane protein 184A [Source:HGNC Symbol;Acc:HGNC:28797]	chr7	-
[131]	FRAS1	ENSG00000138759.19	2.132744	2.26E-11	Fraser extracellular matrix complex subunit 1 [Source:HGNC Symbol;Acc:HGNC:19185]	chr4	+
[132]	KLF5	ENSG00000102554.14	2.130805	2.31E-104	Kruppel like factor 5 [Source:HGNC Symbol;Acc:HGNC:6349]	chr13	+
[133]	PART1	ENSG00000152931.8	2.128041	2.24E-09	prostate androgen-regulated transcript 1 [Source:HGNC Symbol;Acc:HGNC:17263]	chr5	+
[134]	ATF4P4	ENSG00000256167.1	2.127367	8.88E-28	activating transcription factor 4 pseudogene 4 [Source:HGNC Symbol;Acc:HGNC:787]	chr11	+
[135]	AC131160.1	ENSG00000283765.1	2.1264	4.91E-06	novel protein	chr3	-
[136]	KLHDC7B	ENSG00000130487.9	2.124529	4.33E-06	kelch domain containing 7B [Source:HGNC Symbol;Acc:HGNC:25145]	chr22	+
[137]	DCAF4L1	ENSG00000182308.7	2.117241	6.64E-09	DDB1 and CUL4 associated factor 4 like 1 [Source:HGNC Symbol;Acc:HGNC:27723]	chr4	+
[138]	IQCN	ENSG00000130518.17	2.115791	9.95E-30	IQ motif containing N [Source:HGNC Symbol;Acc:HGNC:29350]	chr19	-
[139]	AKNA	ENSG00000106948.16	2.109713	7.66E-29	AT-hook transcription factor [Source:HGNC Symbol;Acc:HGNC:24108]	chr9	-
[140]	SLC6A9	ENSG00000196517.11	2.107995	4.20E-29	solute carrier family 6 member 9 [Source:HGNC Symbol;Acc:HGNC:11056]	chr1	-

[141]	OBSCN	ENSG00000154358.21	2.107337	4.25E-05	obscurin, cytoskeletal calmodulin and titin-interacting RhoGEF [Source:HGNC Symbol;Acc:HGNC:15719]	chr1	+
[142]	PTPRB	ENSG00000127329.15	2.100913	2.36E-15	protein tyrosine phosphatase receptor type B [Source:HGNC Symbol;Acc:HGNC:9665]	chr12	-
[143]	SPOCK2	ENSG00000107742.13	2.09873	6.21E-05	SPARC (osteonectin), cwcv and kazal like domains proteoglycan 2 [Source:HGNC Symbol;Acc:HGNC:13564]	chr10	-
[144]	GADD45G	ENSG00000130222.11	2.096315	6.34E-05	growth arrest and DNA damage inducible gamma [Source:HGNC Symbol;Acc:HGNC:4097]	chr9	+
[145]	N4BP3	ENSG00000145911.6	2.096066	6.36E-05	NEDD4 binding protein 3 [Source:HGNC Symbol;Acc:HGNC:29852]	chr5	+
[146]	ASXL3	ENSG00000141431.12	2.093583	5.16E-06	ASXL transcriptional regulator 3 [Source:HGNC Symbol;Acc:HGNC:29357]	chr18	+
[147]	ATP6V1FNB	ENSG00000272899.4	2.093466	4.73E-25	ATP6V1F neighbor [Source:HGNC Symbol;Acc:HGNC:52392]	chr7	+
[148]	AC068946.3	ENSG00000287529.1	2.093451	6.47E-05	novel transcript, antisense to ZFAND2B	chr2	-
[149]	PRR9	ENSG00000203783.5	2.088022	1.96E-11	proline rich 9 [Source:HGNC Symbol;Acc:HGNC:32057]	chr1	+
[150]	FLG	ENSG00000143631.10	2.086563	1.84E-26	filaggrin [Source:HGNC Symbol;Acc:HGNC:3748]	chr1	-
[151]	NR4A3	ENSG00000119508.18	2.086386	1.49E-16	nuclear receptor subfamily 4 group A member 3 [Source:HGNC Symbol;Acc:HGNC:7982]	chr9	+
[152]	ATF4P3	ENSG00000228218.1	2.081002	5.53E-106	activating transcription factor 4 pseudogene 3 [Source:HGNC Symbol;Acc:HGNC:788]	chr17	-
[153]	AQP3	ENSG00000165272.16	2.074731	5.84E-05	aquaporin 3 (Gill blood group) [Source:HGNC Symbol;Acc:HGNC:636]	chr9	-
[154]	RTL5	ENSG00000242732.4	2.068024	2.46E-08	retrotransposon Gag like 5 [Source:HGNC Symbol;Acc:HGNC:29430]	chrX	-

[155]	AC007728.2	ENSG00000261644.2	2.06681	1.46E-08	CYLD antisense RNA 1 [Source:HGNC Symbol;Acc:HGNC:55352]	chr16	-
[156]	SCIN	ENSG00000006747.15	2.059584	8.68E-05	scinderin [Source:HGNC Symbol;Acc:HGNC:21695]	chr7	+
[157]	AC091230.1	ENSG00000261606.5	2.057144	8.85E-05	novel transcript	chr15	+
[158]	AKR1C1	ENSG00000187134.14	2.05459	0	aldo-keto reductase family 1 member C1 [Source:HGNC Symbol;Acc:HGNC:384]	chr10	+
[159]	NATD1	ENSG00000274180.1	2.049595	5.97E-14	N-acetyltransferase domain containing 1 [Source:HGNC Symbol;Acc:HGNC:30770]	chr17	-
[160]	AC087632.2	ENSG00000259316.11	2.046574	7.62E-05	novel protein	chr15	-
[161]	ATCAY	ENSG00000167654.18	2.037408	0.000104	ATCAY kinesin light chain interacting caytaxin [Source:HGNC Symbol;Acc:HGNC:779]	chr19	+
[162]	DPPA2P4	ENSG00000261429.2	2.034474	6.19E-05	developmental pluripotency associated 2 pseudogene 4 [Source:HGNC Symbol;Acc:HGNC:44629]	chr16	+
[163]	AC244453.2	ENSG00000233029.3	2.033394	0.000103	novel transcript	chr1	-
[164]	AL022326.1	ENSG00000224794.2	2.029977	0.000111	novel transcript	chr22	+
[165]	RRAD	ENSG00000166592.12	2.029252	8.11E-255	RRAD, Ras related glycolysis inhibitor and calcium channel regulator [Source:HGNC Symbol;Acc:HGNC:10446]	chr16	-
[166]	GABARAPL1	ENSG00000139112.11	2.028935	1.13E-128	GABA type A receptor associated protein like 1 [Source:HGNC Symbol;Acc:HGNC:4068]	chr12	+
[167]	PPP1R15A	ENSG00000087074.8	2.023321	0	protein phosphatase 1 regulatory subunit 15A [Source:HGNC Symbol;Acc:HGNC:14375]	chr19	+
[168]	EGLN3	ENSG00000129521.14	2.021904	1.10E-05	egl-9 family hypoxia inducible factor 3 [Source:HGNC Symbol;Acc:HGNC:14661]	chr14	-
[169]	B3GNT4	ENSG00000176383.9	2.015336	6.47E-07	UDP-GlcNAc:betaGal beta-1,3-N-acetylglucosaminyltransferase 4 [Source:HGNC Symbol;Acc:HGNC:15683]	chr12	+
[170]	PNRC1	ENSG00000146278.11	2.011547	1.63E-94	proline rich nuclear receptor coactivator 1 [Source:HGNC Symbol;Acc:HGNC:17278]	chr6	+
[171]	LINC00536	ENSG00000249917.3	2.003938	0.000113	long intergenic non-protein coding RNA 536	chr8	-

					[Source:HGNC Symbol;Acc:HGNC:43645]		
