# Supplementary material for: In Vitro and In Vivo Enhancement of Temozolomide Effect in Human Glioblastoma by Non-Invasive Application of Cold Atmospheric Plasma
Source: Cancers (Basel). 2021 Sep 6;13(17):4485. doi: 10.3390/cancers13174485 (PMC8430547; doi:10.3390/cancers13174485)
Supplement: Supplementary file 1 [file cancers-13-04485-s001.zip › SI Table S2 - Top downregulated genes in CAP treatment.pdf]

Supplementary Table 2: List of top 134 down-regulated genes in CAP treatment

Sr. No	Gene Name	GeneID	Log2(FC)	P-adj	Description	Chromosome	Strand
[1]	AC004949.1	ENSG00000283117.1	-6.9793	1.88E-80	uncharacterized LOC79150 [Source:NCBI gene (formerly Entrezgene);Acc:79150]	chr7	-
[2]	SNORA48	ENSG00000209582.1	-4.63482	1.29E-49	small nucleolar RNA, H/ACA box 48 [Source:HGNC Symbol;Acc:HGNC:32641]	chr17	+
[3]	PIGW	ENSG00000277161.2	-4.48098	5.40E-25	phosphatidylinositol glycan anchor biosynthesis class W [Source:HGNC Symbol;Acc:HGNC:23213]	chr17	+
[4]	SRGAP2D	ENSG00000270872.2	-4.37259	1.31E-23	SLIT-ROBO Rho GTPase activating protein 2D (pseudogene) [Source:HGNC Symbol;Acc:HGNC:43932]	chr1	+
[5]	AC026464.4	ENSG00000260914.3	-4.12949	2.14E-19	novel protein	chr16	+
[6]	SNORA63	ENSG00000200320.1	-3.9004	7.88E-22	small nucleolar RNA, H/ACA box 63 [Source:HGNC Symbol;Acc:HGNC:10106]	chr3	+
[7]	MT-TN	ENSG00000210135.1	-3.60601	3.86E-66	mitochondrially encoded tRNA-Asn (AAU/C) [Source:HGNC Symbol;Acc:HGNC:7493]	chrM	-
[8]	MT-TF	ENSG00000210049.1	-3.60452	6.22E-21	mitochondrially encoded tRNA-Phe (UUU/C) [Source:HGNC Symbol;Acc:HGNC:7481]	chrM	+
[9]	SMIM11B	ENSG00000273590.4	-3.48028	3.41E-13	small integral membrane protein 11B [Source:HGNC Symbol;Acc:HGNC:51846]	chr21	+
[10]	HIST2H3D	ENSG00000183598.3	-3.38011	6.88E-190	H3 clustered histone 13 [Source:HGNC Symbol;Acc:HGNC:25311]	chr1	-
[11]	Y_RNA	ENSG00000200494.1	-3.33084	4.13E-24	Y RNA [Source:RFAM;Acc:RF00019]	chr20	-
[12]	RNY3P1	ENSG00000201955.1	-3.33084	4.13E-24	RNY3 pseudogene 1 [Source:HGNC Symbol;Acc:HGNC:42477]	chr5	-
[13]	RNY3	ENSG00000202354.1	-3.33084	4.13E-24	RNA, Ro60-associated Y3 [Source:HGNC Symbol;Acc:HGNC:10243]	chr7	+
[14]	Y_RNA	ENSG00000207142.1	-3.33084	4.13E-24	Y RNA [Source:RFAM;Acc:RF00019]	chr12	+
[15]	HIST1H3J	ENSG00000197153.4	-3.25795	5.45E-161	H3 clustered histone 12 [Source:HGNC Symbol;Acc:HGNC:4774]	chr6	-
[16]	AC051619.8	ENSG00000260035.1	-3.25346	9.26E-17	novel transcript	chr15	-
[17]	HIST1H2BM	ENSG00000273703.1	-3.24626	6.00E-117	H2B clustered histone 14 [Source:HGNC Symbol;Acc:HGNC:4750]	chr6	+

[18]	HIST1H2AB	ENSG00000278463.1	-3.18572	1.32E-89	H2A clustered histone 4 [Source:HGNC Symbol;Acc:HGNC:4734]	chr6	-
[19]	MT-TA	ENSG00000210127.1	-3.16725	1.45E-22	mitochondrially encoded tRNA-Ala (GCN) [Source:HGNC Symbol;Acc:HGNC:7475]	chrM	-
[20]	HIST1H3I	ENSG00000275379.1	-3.14679	4.97E-65	H3 clustered histone 11 [Source:HGNC Symbol;Acc:HGNC:4771]	chr6	-
[21]	SNORA57	ENSG00000206597.1	-3.13598	6.66E-48	small nucleolar RNA, H/ACA box 57 [Source:HGNC Symbol;Acc:HGNC:32651]	chr11	+
[22]	SNORD10	ENSG00000238917.1	-3.06821	9.92E-13	small nucleolar RNA, C/D box 10 [Source:HGNC Symbol;Acc:HGNC:32706]	chr17	+
[23]	HIST1H2AL	ENSG00000276903.1	-3.03548	1.74E-180	H2A clustered histone 16 [Source:HGNC Symbol;Acc:HGNC:4730]	chr6	+
[24]	HIST1H3G	ENSG00000273983.1	-3.00694	1.20E-228	H3 clustered histone 8 [Source:HGNC Symbol;Acc:HGNC:4772]	chr6	-
[25]	RNU5F-1	ENSG00000199377.1	-2.96175	2.49E-14	RNA, U5F small nuclear 1 [Source:HGNC Symbol;Acc:HGNC:10216]	chr1	-
[26]	C4orf48	ENSG00000243449.6	-2.91532	3.52E-09	chromosome 4 open reading frame 48 [Source:HGNC Symbol;Acc:HGNC:34437]	chr4	+
[27]	RN7SL128P	ENSG00000240869.3	-2.91498	0	RNA, 7SL, cytoplasmic 128, pseudogene [Source:HGNC Symbol;Acc:HGNC:46144]	chr6	-
[28]	HIST1H2BJ	ENSG00000124635.8	-2.91303	2.62E-169	H2B clustered histone 11 [Source:HGNC Symbol;Acc:HGNC:4761]	chr6	-
[29]	MT-ND3	ENSG00000198840.2	-2.90632	1.03E-125	mitochondrially encoded NADH:ubiquinone oxidoreductase core subunit 3 [Source:HGNC Symbol;Acc:HGNC:7458]	chrM	+
[30]	HIST1H3A	ENSG00000275714.1	-2.88092	4.86E-70	H3 clustered histone 1 [Source:HGNC Symbol;Acc:HGNC:4766]	chr6	+
[31]	HIST1H1A	ENSG00000124610.4	-2.85819	1.44E-38	H1.1 linker histone, cluster member [Source:HGNC Symbol;Acc:HGNC:4715]	chr6	-
[32]	HIST2H3DP1	ENSG00000213244.3	-2.85738	2.60E-46	H3 histone pseudogene 4 [Source:HGNC Symbol;Acc:HGNC:43797]	chr1	-
[33]	RNU4-2	ENSG00000202538.1	-2.84925	5.95E-52	RNA, U4 small nuclear 2 [Source:HGNC Symbol;Acc:HGNC:10193]	chr12	-
[34]	RNU4-1	ENSG00000200795.1	-2.81825	3.44E-41	RNA, U4 small nuclear 1 [Source:HGNC Symbol;Acc:HGNC:10192]	chr12	-
[35]	RNU2-2P	ENSG00000222328.1	-2.80875	2.01E-10	RNA, U2 small nuclear 2, pseudogene [Source:HGNC Symbol;Acc:HGNC:10152]	chr11	-

[36]	HIST1H2AI	ENSG00000196747.4	-2.80745	1.19E-196	H2A clustered histone 13 [Source:HGNC Symbol;Acc:HGNC:4725]	chr6	+
[37]	HIST1H4B	ENSG00000278705.1	-2.80472	5.82E-260	H4 clustered histone 2 [Source:HGNC Symbol;Acc:HGNC:4789]	chr6	-
[38]	HIST1H2AG	ENSG00000196787.3	-2.80116	6.76E-266	H2A clustered histone 11 [Source:HGNC Symbol;Acc:HGNC:4737]	chr6	+
[39]	RN7SL674P	ENSG00000239899.3	-2.79438	0	RNA, 7SL, cytoplasmic 674, pseudogene [Source:HGNC Symbol;Acc:HGNC:46690]	chr2	+
[40]	FP671120.2	ENSG00000277437.1	-2.79154	7.00E-11	nan	chr21	+
[41]	SNORA70	ENSG00000207165.1	-2.78785	1.16E-09	small nucleolar RNA, H/ACA box 70 [Source:HGNC Symbol;Acc:HGNC:10231]	chrX	+
[42]	CR392039.1	ENSG00000264063.1	-2.76565	1.15E-10	nan	chr21	+
[43]	HIST2H4B	ENSG00000270276.2	-2.76552	0	H4 clustered histone 15 [Source:HGNC Symbol;Acc:HGNC:29607]	chr1	-
[44]	HIST2H4A	ENSG00000270882.2	-2.76466	0	H4 clustered histone 14 [Source:HGNC Symbol;Acc:HGNC:4794]	chr1	+
[45]	RN7SKP230	ENSG00000202512.1	-2.74653	1.91E-232	RN7SK pseudogene 230 [Source:HGNC Symbol;Acc:HGNC:45954]	chr5	-
[46]	SNORA54	ENSG00000207008.1	-2.73449	2.29E-11	small nucleolar RNA, H/ACA box 54 [Source:HGNC Symbol;Acc:HGNC:32647]	chr11	-
[47]	ASPM	ENSG00000066279.18	-2.72395	0	assembly factor for spindle microtubules [Source:HGNC Symbol;Acc:HGNC:19048]	chr1	-
[48]	PRR11	ENSG00000068489.12	-2.70463	2.88E-127	proline rich 11 [Source:HGNC Symbol;Acc:HGNC:25619]	chr17	+
[49]	AC008894.3	ENSG00000279198.1	-2.70336	3.58E-36	TEC	chr19	-
[50]	HIST1H2BI	ENSG00000278588.1	-2.68702	4.85E-151	H2B clustered histone 10 [Source:HGNC Symbol;Acc:HGNC:4756]	chr6	+
[51]	HIST1H1B	ENSG00000184357.4	-2.67681	0	H1.5 linker histone, cluster member [Source:HGNC Symbol;Acc:HGNC:4719]	chr6	-
[52]	SNORA73A	ENSG00000274266.1	-2.66579	5.85E-239	small nucleolar RNA, H/ACA box 73A [Source:HGNC Symbol;Acc:HGNC:10115]	chr1	+
[53]	SNORA71A	ENSG00000225091.3	-2.65584	6.06E-09	small nucleolar RNA, H/ACA box 71A [Source:HGNC Symbol;Acc:HGNC:10232]	chr20	-
[54]	HIST1H2BF	ENSG00000277224.2	-2.65106	2.80E-128	H2B clustered histone 7 [Source:HGNC Symbol;Acc:HGNC:4752]	chr6	+
[55]	MT-TC	ENSG00000210140.1	-2.63505	6.61E-62	mitochondrially encoded tRNA-Cys (UGU/C)	chrM	-

					[Source:HGNC Symbol;Acc:HGNC:7477]		
[56]	HIST1H2BO	ENSG00000274641.1	-2.61714	8.61E-146	H2B clustered histone 17 [Source:HGNC Symbol;Acc:HGNC:4758]	chr6	+
[57]	HIST1H2AH	ENSG00000274997.1	-2.60995	7.51E-153	H2A clustered histone 12 [Source:HGNC Symbol;Acc:HGNC:13671]	chr6	+
[58]	HIST1H4L	ENSG00000275126.1	-2.58083	3.56E-62	H4 clustered histone 13 [Source:HGNC Symbol;Acc:HGNC:4791]	chr6	-
[59]	HIST1H1D	ENSG00000124575.6	-2.57455	3.94E-280	H1.3 linker histone, cluster member [Source:HGNC Symbol;Acc:HGNC:4717]	chr6	-
[60]	Y_RNA	ENSG00000200314.1	-2.56581	2.27E-09	Y RNA [Source:RFAM;Acc:RF00019]	chr6	+
[61]	HIST1H2AJ	ENSG00000276368.1	-2.56318	1.26E-165	H2A clustered histone 14 [Source:HGNC Symbol;Acc:HGNC:4727]	chr6	-
[62]	HIST1H3C	ENSG00000287080.1	-2.55383	5.81E-256	H3 clustered histone 3 [Source:HGNC Symbol;Acc:HGNC:4768]	chr6	+
[63]	PHLDA2	ENSG00000181649.8	-2.5288	6.64E-07	pleckstrin homoLogy like domain family A member 2 [Source:HGNC Symbol;Acc:HGNC:12385]	chr11	-
[64]	MT-TY	ENSG00000210144.1	-2.50629	3.49E-74	mitochondrially encoded tRNA-Tyr (UAU/C) [Source:HGNC Symbol;Acc:HGNC:7502]	chrM	-
[65]	HIST1H3F	ENSG00000277775.1	-2.48751	1.60E-264	H3 clustered histone 7 [Source:HGNC Symbol;Acc:HGNC:4773]	chr6	-
[66]	HIST2H2AC	ENSG00000184260.5	-2.46797	0	H2A clustered histone 20 [Source:HGNC Symbol;Acc:HGNC:4738]	chr1	+
[67]	Y_RNA	ENSG00000199677.1	-2.46495	1.12E-09	Y RNA [Source:RFAM;Acc:RF00019]	chr5	-
[68]	HERC3	ENSG00000138641.18	-2.45856	7.13E-07	HECT and RLD domain containing E3 ubiquitin protein ligase 3 [Source:HGNC Symbol;Acc:HGNC:4876]	chr4	+
[69]	HIST2H3C	ENSG00000203811.1	-2.45211	0	H3 clustered histone 14 [Source:HGNC Symbol;Acc:HGNC:20503]	chr1	-
[70]	HIST2H3A	ENSG00000203852.3	-2.45172	0	H3 clustered histone 15 [Source:HGNC Symbol;Acc:HGNC:20505]	chr1	+
[71]	SNORA52	ENSG00000199785.1	-2.44577	9.61E-08	small nucleolar RNA, H/ACA box 52 [Source:HGNC Symbol;Acc:HGNC:32645]	chr11	+
[72]	KIF18B	ENSG00000186185.14	-2.43213	2.55E-83	kinesin family member 18B [Source:HGNC Symbol;Acc:HGNC:27102]	chr17	-
[73]	MT-TE	ENSG00000210194.1	-2.43124	1.52E-23	mitochondrially encoded tRNA-Glu (GAA/G)	chrM	-

					[Source:HGNC Symbol;Acc:HGNC:7479]		
[74]	RN7SL767P	ENSG00000241529.3	-2.42491	3.09E-23	RNA, 7SL, cytoplasmic 767, pseudogene [Source:HGNC Symbol;Acc:HGNC:46783]	chr3	+
[75]	RNU5E-1	ENSG00000199347.1	-2.40068	5.49E-08	RNA, U5E small nuclear 1 [Source:HGNC Symbol;Acc:HGNC:10215]	chr1	+
[76]	CENPA	ENSG00000115163.15	-2.39523	4.37E-43	centromere protein A [Source:HGNC Symbol;Acc:HGNC:1851]	chr2	+
[77]	UHRF1	ENSG00000276043.5	-2.39224	1.27E-97	ubiquitin like with PHD and ring finger domains 1 [Source:HGNC Symbol;Acc:HGNC:12556]	chr19	+
[78]	PLK1	ENSG00000166851.15	-2.39024	4.16E-211	polo like kinase 1 [Source:HGNC Symbol;Acc:HGNC:9077]	chr16	+
[79]	NEURL1B	ENSG00000214357.9	-2.34878	3.36E-56	neuralized E3 ubiquitin protein ligase 1B [Source:HGNC Symbol;Acc:HGNC:35422]	chr5	+
[80]	HJURP	ENSG00000123485.12	-2.34196	5.33E-194	Holliday junction recognition protein [Source:HGNC Symbol;Acc:HGNC:25444]	chr2	-
[81]	TOP2A	ENSG00000131747.15	-2.33901	0	DNA topoisomerase II alpha [Source:HGNC Symbol;Acc:HGNC:11989]	chr17	-
[82]	HIST1H2AE	ENSG00000277075.2	-2.33858	1.94E-139	H2A clustered histone 8 [Source:HGNC Symbol;Acc:HGNC:4724]	chr6	+
[83]	RDM1	ENSG00000278023.6	-2.33482	5.98E-06	RAD52 motif containing 1 [Source:HGNC Symbol;Acc:HGNC:19950]	chr17	-
[84]	KIF20A	ENSG00000112984.12	-2.33418	2.75E-167	kinesin family member 20A [Source:HGNC Symbol;Acc:HGNC:9787]	chr5	+
[85]	CENPE	ENSG00000138778.12	-2.32713	0	centromere protein E [Source:HGNC Symbol;Acc:HGNC:1856]	chr4	-
[86]	AC055736.1	ENSG00000257500.1	-2.32144	7.02E-06	novel transcript, antisense to KRT75 and KRT82	chr12	+
[87]	HIST1H2BB	ENSG00000276410.3	-2.31358	2.47E-94	H2B clustered histone 3 [Source:HGNC Symbol;Acc:HGNC:4751]	chr6	-
[88]	POLA2	ENSG00000014138.9	-2.30274	1.65E-25	DNA polymerase alpha 2, accessory subunit [Source:HGNC Symbol;Acc:HGNC:30073]	chr11	+
[89]	SIGLEC15	ENSG00000197046.12	-2.25273	2.77E-157	sialic acid binding Ig like lectin 15 [Source:HGNC Symbol;Acc:HGNC:27596]	chr18	+
[90]	HIST2H2BE	ENSG00000184678.10	-2.2433	2.54E-297	H2B clustered histone 21 [Source:HGNC Symbol;Acc:HGNC:4760]	chr1	-
[91]	SNORA49	ENSG00000208892.1	-2.23619	1.05E-07	small nucleolar RNA, H/ACA box 49	chr12	+

					[Source:HGNC Symbol;Acc:HGNC:32642]		
[92]	LMNB1	ENSG00000113368.12	-2.23459	5.79E-79	lamin B1 [Source:HGNC Symbol;Acc:HGNC:6637]	chr5	+
[93]	HIST1H4A	ENSG00000278637.1	-2.23286	3.48E-44	H4 clustered histone 1 [Source:HGNC Symbol;Acc:HGNC:4781]	chr6	+
[94]	AL162151.2	ENSG00000234648.1	-2.23103	8.62E-11	pseudogene similar to part of ribosomal protein L3 (RPL3)	chr14	+
[95]	HMMR	ENSG00000072571.20	-2.22078	3.10E-146	hyaluronan mediated motility receptor [Source:HGNC Symbol;Acc:HGNC:5012]	chr5	+
[96]	MKI67	ENSG00000148773.14	-2.21612	0	marker of proliferation Ki-67 [Source:HGNC Symbol;Acc:HGNC:7107]	chr10	-
[97]	CDCA8	ENSG00000134690.11	-2.21327	1.34E-132	cell division cycle associated 8 [Source:HGNC Symbol;Acc:HGNC:14629]	chr1	+
[98]	MT-ATP8	ENSG00000228253.1	-2.21192	6.04E-89	mitochondrially encoded ATP synthase membrane subunit 8 [Source:HGNC Symbol;Acc:HGNC:7415]	chrM	+
[99]	HIST2H2AA4	ENSG00000272196.2	-2.18859	0	H2A clustered histone 19 [Source:HGNC Symbol;Acc:HGNC:29668]	chr1	+
[100]	HIST2H2AA3	ENSG00000203812.2	-2.1874	0	H2A clustered histone 18 [Source:HGNC Symbol;Acc:HGNC:4736]	chr1	-
[101]	ARL17B	ENSG00000228696.9	-2.18695	2.76E-05	ADP ribosylation factor like GTPase 17B [Source:HGNC Symbol;Acc:HGNC:32387]	chr17	-
[102]	SCARNA22	ENSG00000249784.1	-2.18425	2.15E-06	small Cajal body-specific RNA 22 [Source:HGNC Symbol;Acc:HGNC:32580]	chr4	+
[103]	BUB1	ENSG00000169679.15	-2.17022	1.68E-220	BUB1 mitotic checkpoint serine/threonine kinase [Source:HGNC Symbol;Acc:HGNC:1148]	chr2	-
[104]	CDC20	ENSG00000117399.14	-2.15594	2.26E-178	cell division cycle 20 [Source:HGNC Symbol;Acc:HGNC:1723]	chr1	+
[105]	HIST1H2BG	ENSG00000273802.2	-2.15481	8.23E-170	H2B clustered histone 8 [Source:HGNC Symbol;Acc:HGNC:4746]	chr6	-
[106]	GTSE1	ENSG00000075218.19	-2.14291	1.56E-79	G2 and S-phase expressed 1 [Source:HGNC Symbol;Acc:HGNC:13698]	chr22	+
[107]	RNY1	ENSG00000201098.1	-2.13736	3.95E-07	RNA, Ro60-associated Y1 [Source:HGNC Symbol;Acc:HGNC:10242]	chr7	-
[108]	AC009119.1	ENSG00000260228.6	-2.12052	5.17E-05	novel transcript, antisense to CDH13	chr16	-
[109]	MT-TL1	ENSG00000209082.1	-2.11931	6.23E-25	mitochondrially encoded tRNA-Leu (UUA/G) 1 [Source:HGNC Symbol;Acc:HGNC:7490]	chrM	+

[110]	FAM110A	ENSG00000125898.13	-2.11612	6.73E-07	family with sequence similarity 110 member A [Source:HGNC Symbol;Acc:HGNC:16188]	chr20	+
[111]	HGF	ENSG00000019991.18	-2.10729	3.08E-88	hepatocyte growth factor [Source:HGNC Symbol;Acc:HGNC:4893]	chr7	-
[112]	SNORA71D	ENSG00000200354.1	-2.10293	5.16E-06	small nucleolar RNA, H/ACA box 71D [Source:HGNC Symbol;Acc:HGNC:32657]	chr20	-
[113]	PSRC1	ENSG00000134222.16	-2.09555	2.02E-59	proline and serine rich coiled-coil 1 [Source:HGNC Symbol;Acc:HGNC:24472]	chr1	-
[114]	CHTF18	ENSG00000127586.17	-2.09224	4.61E-22	chromosome transmission fidelity factor 18 [Source:HGNC Symbol;Acc:HGNC:18435]	chr16	+
[115]	CCNA2	ENSG00000145386.10	-2.09153	5.05E-205	cyclin A2 [Source:HGNC Symbol;Acc:HGNC:1578]	chr4	-
[116]	FOXC2-AS1	ENSG00000260944.1	-2.07957	7.44E-05	FOXC2 antisense RNA 1 [Source:HGNC Symbol;Acc:HGNC:50665]	chr16	-
[117]	CCNF	ENSG00000162063.13	-2.07929	1.17E-58	cyclin F [Source:HGNC Symbol;Acc:HGNC:1591]	chr16	+
[118]	FAM83D	ENSG00000101447.15	-2.07722	5.12E-118	family with sequence similarity 83 member D [Source:HGNC Symbol;Acc:HGNC:16122]	chr20	+
[119]	AC112777.1	ENSG00000256663.1	-2.07368	6.25E-38	ubiquitin-like with PHD and ring finger domains 1 (UHRF1) pseudogene	chr12	+
[120]	HIST1H2AM	ENSG00000278677.1	-2.06322	1.33E-133	H2A clustered histone 17 [Source:HGNC Symbol;Acc:HGNC:4735]	chr6	-
[121]	HIST1H3B	ENSG00000286522.1	-2.06016	1.41E-295	H3 clustered histone 2 [Source:HGNC Symbol;Acc:HGNC:4776]	chr6	-
[122]	ANLN	ENSG00000011426.11	-2.05611	0	anillin actin binding protein [Source:HGNC Symbol;Acc:HGNC:14082]	chr7	+
[123]	CDCA7	ENSG00000144354.14	-2.0559	9.31E-38	cell division cycle associated 7 [Source:HGNC Symbol;Acc:HGNC:14628]	chr2	+
[124]	CCNB1	ENSG00000134057.15	-2.05156	0	cyclin B1 [Source:HGNC Symbol;Acc:HGNC:1579]	chr5	+
[125]	HIST1H3E	ENSG00000274750.2	-2.04388	9.21E-28	H3 clustered histone 6 [Source:HGNC Symbol;Acc:HGNC:4769]	chr6	+
[126]	PODNL1	ENSG00000132000.13	-2.04222	0.000104	podocan like 1 [Source:HGNC Symbol;Acc:HGNC:26275]	chr19	-
[127]	HIST1H2BH	ENSG00000275713.2	-2.04055	3.25E-188	H2B clustered histone 9 [Source:HGNC Symbol;Acc:HGNC:4755]	chr6	+
[128]	ID1	ENSG00000125968.9	-2.03915	2.81E-283	inhibitor of DNA binding 1, HLH protein [Source:HGNC Symbol;Acc:HGNC:5360]	chr20	+

[129]	PALMD	ENSG00000099260.11	-2.03627	1.22E-120	palmdelphin [Source:HGNC Symbol;Acc:HGNC:15846]	chr1	+
[130]	MIR3648-2	ENSG00000264462.1	-2.02883	6.66E-08	microRNA 3648-2 [Source:HGNC Symbol;Acc:HGNC:50843]	chr21	+
[131]	MIR3648-1	ENSG00000275708.1	-2.02883	6.66E-08	microRNA 3648-1 [Source:HGNC Symbol;Acc:HGNC:38941]	chr21	+
[132]	DHRS4	ENSG00000157326.19	-2.02561	5.74E-05	dehydrogenase/reductase 4 [Source:HGNC Symbol;Acc:HGNC:16985]	chr14	+
[133]	SNORA7B	ENSG00000207088.1	-2.02261	4.44E-06	small nucleolar RNA, H/ACA box 7B [Source:HGNC Symbol;Acc:HGNC:32593]	chr3	-
[134]	HIST1H2BE	ENSG00000274290.2	-2.00237	8.89E-64	H2B clustered histone 6 [Source:HGNC Symbol;Acc:HGNC:4753]	chr6	+
