# Supplementary material for: In Vitro and In Vivo Enhancement of Temozolomide Effect in Human Glioblastoma by Non-Invasive Application of Cold Atmospheric Plasma
Source: Cancers (Basel). 2021 Sep 6;13(17):4485. doi: 10.3390/cancers13174485 (PMC8430547; doi:10.3390/cancers13174485)
Supplement: Supplementary file 1 [file cancers-13-04485-s001.zip › SI Table S3 - Top upregulated genes in TMZ treatment.pdf]

Supplementary Table 3: List of top 80 up-regulated genes in TMZ treatment

Sr. No	Gene Name	GeneID	Log2(FC)	P-adj	Description	Chromosome	Strand
[1]	PDXP	ENSG00000241360.2	8.790654	0.456811	pyridoxal phosphatase [Source:HGNC Symbol;Acc:HGNC:30259]	chr22	+
[2]	AC009133.6	ENSG00000280893.1	8.354358	0.249621	novel transcript	chr16	+
[3]	AL671762.1	ENSG00000285565.1	8.009808	-0.70319	novel transcript	chr6	+
[4]	SVIL2P	ENSG00000234814.8	6.91887	0.011704	supervillin family member 2, pseudogene [Source:HGNC Symbol;Acc:HGNC:44959]	chr10	+
[5]	FAM72D	ENSG00000215784.6	6.386992	0.186367	family with sequence similarity 72 member D [Source:HGNC Symbol;Acc:HGNC:33593]	chr1	+
[6]	CDC42EP2	ENSG00000149798.5	5.803778	0.809202	CDC42 effector protein 2 [Source:HGNC Symbol;Acc:HGNC:16263]	chr11	+
[7]	ATP23	ENSG00000166896.9	5.100459	0.099163	ATP23 metallopeptidase and ATP synthase assembly factor homoLog [Source:HGNC Symbol;Acc:HGNC:29452]	chr12	+
[8]	AC092641.1	ENSG00000227176.1	5.073101	0.763694	NSA2 pseudogene 5 [Source:HGNC Symbol;Acc:HGNC:54584]	chr2	-
[9]	AP001092.1	ENSG00000237410.1	4.975375	0.917648	NRXN2 antisense RNA 1 [Source:HGNC Symbol;Acc:HGNC:40416]	chr11	+
[10]	MAGOH3P	ENSG00000270975.1	4.909637	0.984236	mago homoLog 3, pseudogene [Source:HGNC Symbol;Acc:HGNC:20020]	chr14	+
[11]	ELMO3	ENSG00000102890.16	4.755558	0.227039	engulfment and cell motility 3 [Source:HGNC Symbol;Acc:HGNC:17289]	chr16	+
[12]	AP005233.2	ENSG00000260877.2	4.700475	0.964178	novel transcript	chr11	+
[13]	PYGM	ENSG00000068976.14	4.523836	-0.71648	glycogen phosphorylase, muscle associated [Source:HGNC Symbol;Acc:HGNC:9726]	chr11	-
[14]	GDF5	ENSG00000125965.9	4.32972	0.215926	growth differentiation factor 5 [Source:HGNC Symbol;Acc:HGNC:4220]	chr20	-
[15]	ERG	ENSG00000157554.19	4.30041	0.050171	ETS transcription factor ERG [Source:HGNC Symbol;Acc:HGNC:3446]	chr21	-

[16]	NFATC4	ENSG00000100968.14	4.28568	1.328864	nuclear factor of activated T cells 4 [Source:HGNC Symbol;Acc:HGNC:7778]	chr14	+
[17]	AC010624.5	ENSG00000287001.1	4.284199	0.011924	novel transcript	chr19	-
[18]	ENO3	ENSG00000108515.17	4.247756	0.189319	enolase 3 [Source:HGNC Symbol;Acc:HGNC:3354]	chr17	+
[19]	MBOAT1	ENSG00000172197.11	4.191913	0.086531	membrane bound O-acyltransferase domain containing 1 [Source:HGNC Symbol;Acc:HGNC:21579]	chr6	-
[20]	CRYZL2P-SEC16B	ENSG00000254154.8	4.187813	0.075692	CRYZL2P-SEC16B readthrough [Source:HGNC Symbol;Acc:HGNC:53757]	chr1	-
[21]	GRIA3	ENSG00000125675.18	4.16156	0.112235	glutamate ionotropic receptor AMPA type subunit 3 [Source:HGNC Symbol;Acc:HGNC:4573]	chrX	+
[22]	TSSK5P	ENSG00000227473.1	4.027879	0.657715	testis specific serine kinase 5, pseudogene [Source:HGNC Symbol;Acc:HGNC:31931]	chr8	-
[23]	NMU	ENSG00000109255.11	3.993564	0.005343	neuromedin U [Source:HGNC Symbol;Acc:HGNC:7859]	chr4	-
[24]	COL5A3	ENSG00000080573.7	3.97997	0.271222	collagen type V alpha 3 chain [Source:HGNC Symbol;Acc:HGNC:14864]	chr19	-
[25]	OPN1SW	ENSG00000128617.2	3.933764	0.687186	opsin 1, short wave sensitive [Source:HGNC Symbol;Acc:HGNC:1012]	chr7	-
[26]	RPS10-NUDT3	ENSG00000270800.3	3.927109	NA	RPS10-NUDT3 readthrough [Source:HGNC Symbol;Acc:HGNC:49181]	chr6	-
[27]	AC005480.1	ENSG00000258891.1	3.912291	0.77664	novel transcript, antisense to ZNF410	chr14	-
[28]	HIF1A-AS3	ENSG00000258667.2	3.904495	0.954607	HIF1A antisense RNA 3 [Source:HGNC Symbol;Acc:HGNC:54284]	chr14	-
[29]	C1QTNF9B	ENSG00000205863.10	3.897132	0.192879	C1q and TNF related 9B [Source:HGNC Symbol;Acc:HGNC:34072]	chr13	-
[30]	C1orf116	ENSG00000182795.13	3.8802	NA	chromosome 1 open reading frame 116 [Source:HGNC Symbol;Acc:HGNC:28667]	chr1	-
[31]	EIF4A1P9	ENSG00000262953.1	3.870801	0.883357	eukaryotic translation initiation factor 4A1 pseudogene 9 [Source:HGNC Symbol;Acc:HGNC:37928]	chr17	-

[32]	DMBX1	ENSG00000197587.10	3.829056	1.283838	diencephalon/mesencephalon homeobox 1 [Source:HGNC Symbol;Acc:HGNC:19026]	chr1	+
[33]	AC013643.2	ENSG00000253875.2	3.818946	0.775396	novel transcript	chr8	+
[34]	CARD16	ENSG00000204397.8	3.728535	0.900266	caspase recruitment domain family member 16 [Source:HGNC Symbol;Acc:HGNC:33701]	chr11	-
[35]	PLSCR3	ENSG00000187838.17	3.707013	0.528082	phospholipid scramblase 3 [Source:HGNC Symbol;Acc:HGNC:16495]	chr17	-
[36]	5_8S_rRNA	ENSG00000277739.1	3.697777	0.620011	5.8S ribosomal RNA [Source:RFAM;Acc:RF00002]	chr21	+
[37]	RNA5-8SN1	ENSG00000278189.1	3.697777	0.620011	RNA, 5.8S ribosomal N1 [Source:HGNC Symbol;Acc:HGNC:53517]	chr21	+
[38]	RNA5-8SN2	ENSG00000278233.1	3.697777	0.620011	RNA, 5.8S ribosomal N2 [Source:HGNC Symbol;Acc:HGNC:53521]	chr21	+
[39]	RNA5-8SN3	ENSG00000275215.1	3.697777	0.620011	RNA, 5.8S ribosomal N3 [Source:HGNC Symbol;Acc:HGNC:53525]	chr21	+
[40]	BMS1P1	ENSG00000204177.10	3.681547	1.382871	BMS1 pseudogene 1 [Source:HGNC Symbol;Acc:HGNC:23649]	chr10	+
[41]	AP001042.1	ENSG00000205622.11	3.666836	0.484591	novel transcript	chr21	-
[42]	AC008163.1	ENSG00000233491.8	3.654234	0.079642	novel pseudogene containing cadherin repeats	chr7	-
[43]	HSPB6	ENSG00000004776.13	3.650925	0.107049	heat shock protein family B (small) member 6 [Source:HGNC Symbol;Acc:HGNC:26511]	chr19	-
[44]	SLC14A1	ENSG00000141469.18	3.595615	0.60588	solute carrier family 14 member 1 (Kidd blood group) [Source:HGNC Symbol;Acc:HGNC:10918]	chr18	+
[45]	PMF1-BGLAP	ENSG00000260238.6	3.586703	1.17072	PMF1-BGLAP readthrough [Source:HGNC Symbol;Acc:HGNC:42953]	chr1	+
[46]	XDH	ENSG00000158125.10	3.561086	-0.63873	xanthine dehydrogenase [Source:HGNC Symbol;Acc:HGNC:12805]	chr2	-
[47]	MSH5-SAPCD1	ENSG00000255152.8	3.445346	1.098256	MSH5-SAPCD1 readthrough (NMD candidate) [Source:HGNC Symbol;Acc:HGNC:41994]	chr6	+

[48]	DNLZ	ENSG00000213221.5	3.441806	0.020381	DNL-type zinc finger [Source:HGNC Symbol;Acc:HGNC:33879]	chr9	-
[49]	GNB3	ENSG00000111664.10	3.438486	0.006444	G protein subunit beta 3 [Source:HGNC Symbol;Acc:HGNC:4400]	chr12	+
[50]	GRIK5	ENSG00000105737.9	3.42963	-0.38605	glutamate ionotropic receptor kainate type subunit 5 [Source:HGNC Symbol;Acc:HGNC:4583]	chr19	-
[51]	AC009542.1	ENSG00000231794.5	3.424139	0.649091	novel transcript, antisense to WDR91	chr7	+
[52]	AC079313.1	ENSG00000258086.1	3.400609	0.07073	novel transcript, antisense to ZNF385A, GPR84, ITGA5 and GTSF1	chr12	+
[53]	PPFIA2	ENSG00000139220.16	3.396283	0.140413	PTPRF interacting protein alpha 2 [Source:HGNC Symbol;Acc:HGNC:9246]	chr12	-
[54]	POC1B-GALNT4	ENSG00000259075.6	3.346762	NA	POC1B-GALNT4 readthrough [Source:HGNC Symbol;Acc:HGNC:42957]	chr12	-
[55]	ADGRF2	ENSG00000164393.8	3.335385	0.284489	adhesion G protein-coupled receptor F2 [Source:HGNC Symbol;Acc:HGNC:18991]	chr6	+
[56]	Y_RNA	ENSG00000200419.1	3.32602	0.032089	Y RNA [Source:RFAM;Acc:RF00019]	chr15	+
[57]	AL591684.2	ENSG00000254929.6	3.310497	0.933861	novel transcript	chr10	-
[58]	PGGHG	ENSG00000142102.16	3.298142	0.869404	protein-glucosylgalactosylhydroxylysine glucosidase [Source:HGNC Symbol;Acc:HGNC:26210]	chr11	+
[59]	ELN-AS1	ENSG00000232415.1	3.265965	0.99909	ELN antisense RNA 1 [Source:HGNC Symbol;Acc:HGNC:40212]	chr7	-
[60]	PEAR1	ENSG00000187800.13	3.256126	-0.53419	platelet endothelial aggregation receptor 1 [Source:HGNC Symbol;Acc:HGNC:33631]	chr1	+
[61]	SNORA80A	ENSG00000200792.1	3.239645	0.000187	small nucleolar RNA, H/ACA box 80A [Source:HGNC Symbol;Acc:HGNC:32666]	chr21	-
[62]	TICAM2	ENSG00000243414.5	3.237514	NA	toll like receptor adaptor molecule 2 [Source:HGNC Symbol;Acc:HGNC:21354]	chr5	-
[63]	TMPRSS11E	ENSG00000087128.10	3.226192	NA	transmembrane serine protease 11E [Source:HGNC Symbol;Acc:HGNC:24465]	chr4	+

[64]	DRGX	ENSG00000165606.8	3.189911	-0.00052	dorsal root ganglia homeobox [Source:HGNC Symbol;Acc:HGNC:21536]	chr10	-
[65]	ASGR1	ENSG00000141505.12	3.154534	0.605036	asiaLoglycoprotein receptor 1 [Source:HGNC Symbol;Acc:HGNC:742]	chr17	-
[66]	TMPRSS11F	ENSG00000198092.6	3.139908	-1.18228	transmembrane serine protease 11F [Source:HGNC Symbol;Acc:HGNC:29994]	chr4	-
[67]	RN7SL823P	ENSG00000263595.2	3.133108	0.003036	RNA, 7SL, cytoplasmic 823, pseudogene [Source:HGNC Symbol;Acc:HGNC:46839]	chr19	-
[68]	AL512603.1	ENSG00000286442.1	3.097356	0.280014	novel transcript	chr10	+
[69]	GREM2	ENSG00000180875.5	3.080702	0.146003	gremlin 2, DAN family BMP antagonist [Source:HGNC Symbol;Acc:HGNC:17655]	chr1	-
[70]	ZBTB9	ENSG00000213588.6	3.071934	1.141274	zinc finger and BTB domain containing 9 [Source:HGNC Symbol;Acc:HGNC:28323]	chr6	+
[71]	RN7SL217P	ENSG00000264706.2	3.067268	0.036611	RNA, 7SL, cytoplasmic 217, pseudogene [Source:HGNC Symbol;Acc:HGNC:46233]	chr3	-
[72]	EPSTI1	ENSG00000133106.14	3.063561	0.192257	epithelial stromal interaction 1 [Source:HGNC Symbol;Acc:HGNC:16465]	chr13	-
[73]	RN7SKP230	ENSG00000202512.1	3.05839	NA	RN7SK pseudogene 230 [Source:HGNC Symbol;Acc:HGNC:45954]	chr5	-
[74]	RN7SL77P	ENSG00000240837.3	3.057757	0.083916	RNA, 7SL, cytoplasmic 77, pseudogene [Source:HGNC Symbol;Acc:HGNC:46093]	chr14	+
[75]	MYO16	ENSG00000041515.16	3.051369	-0.69228	myosin XVI [Source:HGNC Symbol;Acc:HGNC:29822]	chr13	+
[76]	AC093512.2	ENSG00000285043.1	3.044571	0.736987	novel protein	chr16	+
[77]	PRSS12	ENSG00000164099.3	3.038351	0.558723	serine protease 12 [Source:HGNC Symbol;Acc:HGNC:9477]	chr4	-
[78]	TRIM55	ENSG00000147573.17	3.037922	NA	tripartite motif containing 55 [Source:HGNC Symbol;Acc:HGNC:14215]	chr8	+
[79]	MYPN	ENSG00000138347.15	3.03143	0.00049	myopalladin [Source:HGNC Symbol;Acc:HGNC:23246]	chr10	+
[80]	DOK2	ENSG00000147443.13	3.021816	0.218979	docking protein 2 [Source:HGNC Symbol;Acc:HGNC:2991]	chr8	-
