# Supplementary material for: In Vitro and In Vivo Enhancement of Temozolomide Effect in Human Glioblastoma by Non-Invasive Application of Cold Atmospheric Plasma
Source: Cancers (Basel). 2021 Sep 6;13(17):4485. doi: 10.3390/cancers13174485 (PMC8430547; doi:10.3390/cancers13174485)
Supplement: Supplementary file 1 [file cancers-13-04485-s001.zip › SI Table S4 - Top downregulated genes in TMZ treatment.pdf]

Supplementary Table 4: List of top 97 down-regulated genes in TMZ treatment

Sr. No	Gene Name	GeneID	Log2(FC)	P-adj	Description	Chromosome	Strand
[1]	FRG2	ENSG00000205097.6	-8.7864	0.132686	FSHD region gene 2 [Source:HGNC Symbol;Acc:HGNC:19136]	chr4	-
[2]	KRT16	ENSG00000186832.9	-6.68282	0.08388	keratin 16 [Source:HGNC Symbol;Acc:HGNC:6423]	chr17	-
[3]	UCA1	ENSG00000214049.8	-6.15995	0.034703	urothelial cancer associated 1 [Source:HGNC Symbol;Acc:HGNC:37126]	chr19	+
[4]	RPL12P42	ENSG00000213253.5	-6.05765	0.503126	ribosomal protein L12 pseudogene 42 [Source:HGNC Symbol;Acc:HGNC:36165]	chr19	-
[5]	AC026464.4	ENSG00000260914.3	-5.77026	-1.16457	novel protein	chr16	+
[6]	SRGAP2D	ENSG00000270872.2	-5.76371	-0.91615	SLIT-ROBO Rho GTPase activating protein 2D (pseudogene) [Source:HGNC Symbol;Acc:HGNC:43932]	chr1	+
[7]	IGSF9	ENSG00000085552.17	-5.74445	0.284834	immunoglobulin superfamily member 9 [Source:HGNC Symbol;Acc:HGNC:18132]	chr1	-
[8]	USH1G	ENSG00000182040.9	-5.73359	0.461178	USH1 protein network component sans [Source:HGNC Symbol;Acc:HGNC:16356]	chr17	-
[9]	COL26A1	ENSG00000160963.14	-5.52236	0.840672	collagen type XXVI alpha 1 chain [Source:HGNC Symbol;Acc:HGNC:18038]	chr7	+
[10]	SPANXN5	ENSG00000204363.5	-5.44722	0.840672	SPANX family member N5 [Source:HGNC Symbol;Acc:HGNC:33178]	chrX	-
[11]	LINC02158	ENSG00000225611.1	-5.4109	0.136383	long intergenic non-protein coding RNA 2158 [Source:HGNC Symbol;Acc:HGNC:53019]	chr3	+
[12]	PIGW	ENSG00000277161.2	-5.38043	-0.4541	phosphatidylinositol glycan anchor biosynthesis class W [Source:HGNC Symbol;Acc:HGNC:23213]	chr17	+
[13]	TMEM256-PLSCR3	ENSG00000262481.5	-5.21211	0.120749	TMEM256-PLSCR3 readthrough (NMD	chr17	-

					candidate) [Source:HGNC Symbol;Acc:HGNC:49186]		
[14]	ITGAM	ENSG00000169896.18	-5.19877	0.000669	integrin subunit alpha M [Source:HGNC Symbol;Acc:HGNC:6149]	chr16	+
[15]	SUN3	ENSG00000164744.13	-5.12938	0.091184	Sad1 and UNC84 domain containing 3 [Source:HGNC Symbol;Acc:HGNC:22429]	chr7	-
[16]	AC008060.1	ENSG00000218672.1	-5.01584	0.279553	novel transcript	chr7	+
[17]	EEF1AKMT4-ECE2	ENSG00000284917.1	-4.99906	0.531394	EEF1AKMT4-ECE2 readthrough [Source:HGNC Symbol;Acc:HGNC:53615]	chr3	+
[18]	IGFL2-AS1	ENSG00000268621.6	-4.91773	9.04E-11	IGFL2 antisense RNA 1 [Source:HGNC Symbol;Acc:HGNC:52559]	chr19	-
[19]	AC087203.2	ENSG00000255556.2	-4.80291	0.406698	ubiquitin specific peptidase 17-like 2 (USP17L2) pseudogene	chr8	-
[20]	TTYH2	ENSG00000141540.11	-4.67	0.292644	tweety family member 2 [Source:HGNC Symbol;Acc:HGNC:13877]	chr17	+
[21]	DPEP2	ENSG00000167261.14	-4.46583	0.081606	dipeptidase 2 [Source:HGNC Symbol;Acc:HGNC:23028]	chr16	-
[22]	AC010307.3	ENSG00000249478.1	-4.41534	0.00128	novel transcript	chr5	+
[23]	ZPLD1	ENSG00000170044.8	-4.40466	0.84065	zona pellucida like domain containing 1 [Source:HGNC Symbol;Acc:HGNC:27022]	chr3	+
[24]	AC243772.2	ENSG00000233030.2	-4.33069	0.345848	novel transcript, antisense to FCGR1A	chr1	-
[25]	AC087203.1	ENSG00000254423.1	-4.314	0.513816	ubiquitin specific peptidase 17-like 2 (USP17L2) pseudogene	chr8	-
[26]	RSAD2	ENSG00000134321.12	-4.25453	0.466361	radical S-adenosyl methionine domain containing 2 [Source:HGNC Symbol;Acc:HGNC:30908]	chr2	+
[27]	ADAP2	ENSG00000184060.11	-4.2458	0.242928	ArfGAP with dual PH domains 2 [Source:HGNC Symbol;Acc:HGNC:16487]	chr17	+
[28]	ZNF606	ENSG00000166704.11	-4.17544	0.865038	zinc finger protein 606 [Source:HGNC Symbol;Acc:HGNC:25879]	chr19	-
[29]	AGT	ENSG00000135744.8	-4.13111	1.221965	angiotensinogen [Source:HGNC Symbol;Acc:HGNC:333]	chr1	-

[30]	LINC01979	ENSG00000262585.1	-4.1248	0.464105	long intergenic non-protein coding RNA 1979 [Source:HGNC Symbol;Acc:HGNC:52807]	chr17	-
[31]	AL356804.1	ENSG00000259033.2	-4.09553	0.010383	novel transcript	chr14	+
[32]	USP17L7	ENSG00000226430.6	-4.09284	0.166146	ubiquitin specific peptidase 17 like family member 7 [Source:HGNC Symbol;Acc:HGNC:37180]	chr8	-
[33]	AC007317.1	ENSG00000285898.1	-4.0806	0.386161	Novel transcript	chr2	+
[34]	ZNF114	ENSG00000178150.10	-4.07478	1.057927	zinc finger protein 114 [Source:HGNC Symbol;Acc:HGNC:12894]	chr19	+
[35]	AC116317.1	ENSG00000286176.2	-3.9913	1.24E-08	novel transcript	chr4	-
[36]	FRG2C	ENSG00000172969.7	-3.97335	0.000402	FSHD region gene 2 family member C [Source:HGNC Symbol;Acc:HGNC:33626]	chr3	+
[37]	CLDN1	ENSG00000163347.6	-3.96997	0.529157	claudin 1 [Source:HGNC Symbol;Acc:HGNC:2032]	chr3	-
[38]	FRG2B	ENSG00000225899.8	-3.94699	4.50E-17	FSHD region gene 2 family member B [Source:HGNC Symbol;Acc:HGNC:33518]	chr10	-
[39]	DNAH3	ENSG00000158486.13	-3.94657	0.265449	dynein axonemal heavy chain 3 [Source:HGNC Symbol;Acc:HGNC:2949]	chr16	-
[40]	FOXJ1	ENSG00000129654.8	-3.92186	0.550647	forkhead box J1 [Source:HGNC Symbol;Acc:HGNC:3816]	chr17	-
[41]	CEMP1	ENSG00000205923.3	-3.87666	0.499586	cementum protein 1 [Source:HGNC Symbol;Acc:HGNC:32553]	chr16	-
[42]	PIP5K1B	ENSG00000107242.19	-3.8652	0.706124	phosphatidylinositol-4-phosphate 5-kinase type 1 beta [Source:HGNC Symbol;Acc:HGNC:8995]	chr9	+
[43]	BIRC3	ENSG00000023445.14	-3.85706	0.870428	baculoviral IAP repeat containing 3 [Source:HGNC Symbol;Acc:HGNC:591]	chr11	+
[44]	AC087428.1	ENSG00000287505.1	-3.81978	0.647273	novel transcript	chr3	-
[45]	CCR2	ENSG00000121807.5	-3.77359	0.550516	C-C motif chemokine receptor 2 [Source:HGNC Symbol;Acc:HGNC:1603]	chr3	+
[46]	KRTAP21-2	ENSG00000187026.3	-3.75421	0.207967	keratin associated protein 21-2 [Source:HGNC Symbol;Acc:HGNC:18946]	chr21	-

[47]	ADGRD1	ENSG00000111452.13	-3.74749	0.458347	adhesion G protein-coupled receptor D1 [Source:HGNC Symbol;Acc:HGNC:19893]	chr12	+
[48]	ZFHX2	ENSG00000136367.14	-3.72835	1.094583	zinc finger homeobox 2 [Source:HGNC Symbol;Acc:HGNC:20152]	chr14	-
[49]	VTCN1	ENSG00000134258.17	-3.66681	0.109916	V-set domain containing T cell activation inhibitor 1 [Source:HGNC Symbol;Acc:HGNC:28873]	chr1	-
[50]	PPP1R36	ENSG00000165807.8	-3.65104	0.039906	protein phosphatase 1 regulatory subunit 36 [Source:HGNC Symbol;Acc:HGNC:20097]	chr14	+
[51]	AL354872.2	ENSG00000271992.1	-3.6214	0.002496	novel transcript	chr1	+
[52]	CYP4F11	ENSG00000171903.16	-3.62111	3.24E-10	cytochrome P450 family 4 subfamily F member 11 [Source:HGNC Symbol;Acc:HGNC:13265]	chr19	-
[53]	MIR191	ENSG00000207605.3	-3.60233	0.289471	microRNA 191 [Source:HGNC Symbol;Acc:HGNC:31561]	chr3	-
[54]	ACAN	ENSG00000157766.17	-3.59056	0.646842	aggrecan [Source:HGNC Symbol;Acc:HGNC:319]	chr15	+
[55]	SPINK1	ENSG00000164266.10	-3.56928	1.57E-25	serine peptidase inhibitor Kazal type 1 [Source:HGNC Symbol;Acc:HGNC:11244]	chr5	-
[56]	AC011462.1	ENSG00000255730.5	-3.54558	NA	novel protein	chr19	+
[57]	AL139130.1	ENSG00000237390.1	-3.53887	0.992135	novel transcript	chr1	+
[58]	FAM71A	ENSG00000162771.8	-3.5218	0.003251	family with sequence similarity 71 member A [Source:HGNC Symbol;Acc:HGNC:26541]	chr1	+
[59]	KRTAP13-2	ENSG00000182816.9	-3.51667	0.646609	keratin associated protein 13-2 [Source:HGNC Symbol;Acc:HGNC:18923]	chr21	-
[60]	DISP3	ENSG00000204624.8	-3.50273	0.465766	dispatched RND transporter family member 3 [Source:HGNC Symbol;Acc:HGNC:29251]	chr1	+
[61]	GNG7	ENSG00000176533.13	-3.46381	0.000806	G protein subunit gamma 7 [Source:HGNC Symbol;Acc:HGNC:4410]	chr19	-
[62]	AL160269.1	ENSG00000285269.2	-3.45184	0.224817	novel transcript, SLC35D2-HSD17B3 readthrough	chr9	-

[63]	ADAM30	ENSG00000134249.7	-3.44707	0.839064	ADAM metallopeptidase domain 30 [Source:HGNC Symbol;Acc:HGNC:208]	chr1	-
[64]	WFIKKN2	ENSG00000173714.8	-3.44707	0.839064	WAP, follistatin/kazal, immunoglobulin, kunitz and netrin domain containing 2 [Source:HGNC Symbol;Acc:HGNC:30916]	chr17	+
[65]	MT1JP	ENSG00000255986.6	-3.43573	0.008158	metallothionein 1J, pseudogene [Source:HGNC Symbol;Acc:HGNC:7402]	chr16	+
[66]	ZDHHC11B	ENSG00000206077.11	-3.42131	0.001183	zinc finger DHHC-type containing 11B [Source:HGNC Symbol;Acc:HGNC:32962]	chr5	-
[67]	GADD45G	ENSG00000130222.11	-3.38139	1.86E-05	growth arrest and DNA damage inducible gamma [Source:HGNC Symbol;Acc:HGNC:4097]	chr9	+
[68]	RIMKLA	ENSG00000177181.15	-3.35908	1.405643	ribosomal modification protein rimK like family member A [Source:HGNC Symbol;Acc:HGNC:28725]	chr1	+
[69]	LINC00917	ENSG00000168367.11	-3.35592	0.828759	long intergenic non-protein coding RNA 917 [Source:HGNC Symbol;Acc:HGNC:48607]	chr16	-
[70]	HIPK4	ENSG00000160396.9	-3.34562	0.078817	homeodomain interacting protein kinase 4 [Source:HGNC Symbol;Acc:HGNC:19007]	chr19	-
[71]	MRGPRE	ENSG00000184350.11	-3.32657	1.217165	MAS related GPR family member E [Source:HGNC Symbol;Acc:HGNC:30694]	chr11	-
[72]	IGF2	ENSG00000167244.21	-3.32608	7.76E-05	insulin like growth factor 2 [Source:HGNC Symbol;Acc:HGNC:5466]	chr11	-
[73]	AL031590.1	ENSG00000284633.1	-3.32485	0.001218	novel transcript, antisense to PDGFB	chr22	+
[74]	CNTN4	ENSG00000144619.15	-3.32029	0.282148	contactin 4 [Source:HGNC Symbol;Acc:HGNC:2174]	chr3	+
[75]	U3	ENSG00000207119.2	-3.31464	0.397104	Small nucleolar RNA U3 [Source:RFAM;Acc:RF00012]	chr15	-
[76]	LINC01647	ENSG00000235643.2	-3.29703	1.60E-09	long intergenic non-protein coding RNA 1647 [Source:HGNC Symbol;Acc:HGNC:52434]	chr1	+

[77]	CD248	ENSG00000174807.4	-3.28354	1.458435	CD248 molecule [Source:HGNC Symbol;Acc:HGNC:18219]	chr11	-
[78]	AC008969.1	ENSG00000176593.8	-3.25031	0.837431	novel transcript, antisense to ZNF606	chr19	+
[79]	AC068946.3	ENSG00000287529.1	-3.23906	1.90E-05	novel transcript, antisense to ZFAND2B	chr2	-
[80]	AC004453.2	ENSG00000279853.1	-3.23473	0.542367	tec	chr7	-
[81]	SPRR2D	ENSG00000163216.7	-3.22746	1.27E-06	small proline rich protein 2D [Source:HGNC Symbol;Acc:HGNC:11264]	chr1	-
[82]	ATP1B2	ENSG00000129244.9	-3.22218	2.16E-06	ATPase Na+/K+ transporting subunit beta 2 [Source:HGNC Symbol;Acc:HGNC:805]	chr17	+
[83]	LINC01655	ENSG00000227925.2	-3.20261	1.053869	long intergenic non-protein coding RNA 1655 [Source:HGNC Symbol;Acc:HGNC:52443]	chr1	-
[84]	THSD7A	ENSG00000005108.16	-3.19182	0.404926	thrombospondin type 1 domain containing 7A [Source:HGNC Symbol;Acc:HGNC:22207]	chr7	-
[85]	ST6GALNAC1	ENSG00000070526.15	-3.1767	0.644325	ST6 N-acetylgalactosaminide alpha-2,6-sialyltransferase 1 [Source:HGNC Symbol;Acc:HGNC:23614]	chr17	-
[86]	TBC1D29P	ENSG00000266733.7	-3.17261	0.387559	TBC1 domain family member 29, pseudogene [Source:HGNC Symbol;Acc:HGNC:24509]	chr17	+
[87]	AC004835.1	ENSG00000230392.1	-3.1628	0.003573	novel transcript	chrX	+
[88]	AL022476.1	ENSG00000230319.1	-3.16201	1.07103	TTLL1 antisense RNA 1 [Source:HGNC Symbol;Acc:HGNC:40111]	chr22	+
[89]	RNU2-62P	ENSG00000222800.1	-3.16022	1.053147	RNA, U2 small nuclear 62, pseudogene [Source:HGNC Symbol;Acc:HGNC:48555]	chr6	-
[90]	ANKFN1	ENSG00000153930.12	-3.15852	0.012759	ankyrin repeat and fibronectin type III domain containing 1 [Source:HGNC Symbol;Acc:HGNC:26766]	chr17	+
[91]	AC024082.1	ENSG00000231923.1	-3.14161	1.212761	Der1-like domain family, member 2 (DERL2) pseudogene	chr7	+
[92]	DRAXIN	ENSG00000162490.7	-3.14049	0.880859	dorsal inhibitory axon guidance protein	chr1	+

					[Source:HGNC Symbol;Acc:HGNC:25054]		
[93]	DYDC2	ENSG00000133665.13	-3.12557	0.438439	DPY30 domain containing 2 [Source:HGNC Symbol;Acc:HGNC:23468]	chr10	+
[94]	OLAH	ENSG00000152463.14	-3.10515	0.224076	oleoyl-ACP hydrolase [Source:HGNC Symbol;Acc:HGNC:25625]	chr10	+
[95]	CEACAM22P	ENSG00000230666.5	-3.04973	1.206904	CEA cell adhesion molecule 22, pseudogene [Source:HGNC Symbol;Acc:HGNC:38029]	chr19	-
[96]	DHRS2	ENSG00000100867.14	-3.02059	0.026003	dehydrogenase/reductase 2 [Source:HGNC Symbol;Acc:HGNC:18349]	chr14	+
[97]	GSTM3	ENSG00000134202.11	-3.01131	0.182558	glutathione S-transferase mu 3 [Source:HGNC Symbol;Acc:HGNC:4635]	chr1	-
