# Supplementary material for: In Vitro and In Vivo Enhancement of Temozolomide Effect in Human Glioblastoma by Non-Invasive Application of Cold Atmospheric Plasma
Source: Cancers (Basel). 2021 Sep 6;13(17):4485. doi: 10.3390/cancers13174485 (PMC8430547; doi:10.3390/cancers13174485)
Supplement: Supplementary file 1 [file cancers-13-04485-s001.zip › SI Table S5 - Top upregulated genes in CAP + TMZ treatment.pdf]

## Supplementary Table 5: List of top 462 up-regulated genes in the case of CAP + TMZ treatment

| Sr. No | Gene Name | GeneID | Log2(FC) | P-adj | Description | Chromosome | Strand |
|---|---|---|---|---|---|---|---|
| [1] | RASD1 | ENSG00000108551.5 | 7.083646 | 2.39E-100 | ras related dexamethasone induced 1 [Source:HGNC Symbol;Acc:HGNC:15828] | chr17 | - |
| [2] | HSPA6 | ENSG00000173110.8 | 6.609536 | 3.37E-222 | heat shock protein family A (Hsp70) member 6 [Source:HGNC Symbol;Acc:HGNC:5239] | chr1 | + |
| [3] | FRG2 | ENSG00000205097.6 | 6.483626 | 2.57E-33 | FSHD region gene 2 [Source:HGNC Symbol;Acc:HGNC:19136] | chr4 | - |
| [4] | FRG2B | ENSG00000225899.8 | 6.153196 | 9.42E-43 | FSHD region gene 2 family member B [Source:HGNC Symbol;Acc:HGNC:33518] | chr10 | - |
| [5] | SNAI1 | ENSG00000124216.4 | 6.06363 | 8.96E-104 | snail family transcriptional repressor 1 [Source:HGNC Symbol;Acc:HGNC:11128] | chr20 | + |
| [6] | IGFL2-AS1 | ENSG00000268621.6 | 5.91067 | 1.55E-63 | IGFL2 antisense RNA 1 [Source:HGNC Symbol;Acc:HGNC:52559] | chr19 | - |
| [7] | HSPA7 | ENSG00000225217.1 | 5.88534 | 5.45E-76 | heat shock protein family A (Hsp70) member 7 (pseudogene) [Source:HGNC Symbol;Acc:HGNC:5240] | chr1 | + |
| [8] | HSPA8P8 | ENSG00000229091.3 | 5.717379 | 5.35E-78 | heat shock protein family A (Hsp70) member 8 pseudogene 8 [Source:HGNC Symbol;Acc:HGNC:44923] | chr7 | + |
| [9] | AC116317.1 | ENSG00000286176.2 | 5.418042 | 2.18E-20 | novel transcript | chr4 | - |
| [10] | KRT16 | ENSG00000186832.9 | 5.280486 | 2.29E-21 | keratin 16 [Source:HGNC Symbol;Acc:HGNC:6423] | chr17 | - |
| [11] | CYP4F11 | ENSG00000171903.16 | 5.258774 | 5.25E-28 | cytochrome P450 family 4 subfamily F member 11 [Source:HGNC Symbol;Acc:HGNC:13265] | chr19 | - |
| [12] | RGS16 | ENSG00000143333.7 | 5.25644 | 1.01E-39 | regulator of G protein signaling 16 [Source:HGNC Symbol;Acc:HGNC:9997] | chr1 | - |
| [13] | CDH16 | ENSG00000166589.13 | 5.164967 | 3.82E-18 | cadherin 16 [Source:HGNC Symbol;Acc:HGNC:1755] | chr16 | - |
| [14] | ITGAM | ENSG00000169896.18 | 5.101083 | 1.30E-17 | integrin subunit alpha M [Source:HGNC Symbol;Acc:HGNC:6149] | chr16 | + |
| [15] | LINC01647 | ENSG00000235643.2 | 5.088052 | 1.71E-17 | long intergenic non-protein coding RNA 1647 [Source:HGNC Symbol;Acc:HGNC:52434] | chr1 | + |
| [16] | UCA1 | ENSG00000214049.8 | 5.053508 | 3.54E-17 | urothelial cancer associated 1 [Source:HGNC Symbol;Acc:HGNC:37126] | chr19 | + |
| [17] | TEX14 | ENSG00000121101.15 | 5.0246 | 5.75E-17 | testis expressed 14, intercellular bridge forming | chr17 | - |

| | | | | | | | |
|---|---|---|---|---|---|---|---|
| | | | | | factor [Source:HGNC Symbol;Acc:HGNC:11737] | | |
| [18] | FAM166A | ENSG00000188163.8 | 4.947647 | 4.86E-60 | family with sequence similarity 166 member A [Source:HGNC Symbol;Acc:HGNC:33818] | chr9 | - |
| [19] | VLDLR-AS1 | ENSG00000236404.10 | 4.689785 | 5.42E-21 | VLDLR antisense RNA 1 [Source:HGNC Symbol;Acc:HGNC:49621] | chr9 | - |
| [20] | KCNB1 | ENSG00000158445.10 | 4.664124 | 1.84E-30 | potassium voltage-gated channel subfamily B member 1 [Source:HGNC Symbol;Acc:HGNC:6231] | chr20 | - |
| [21] | SPINK1 | ENSG00000164266.10 | 4.64754 | 9.86E-169 | serine peptidase inhibitor Kazal type 1 [Source:HGNC Symbol;Acc:HGNC:11244] | chr5 | - |
| [22] | UBE2F-SCLY | ENSG00000258984.5 | 4.597201 | 1.36E-14 | UBE2F-SCLY readthrough (NMD candidate) [Source:HGNC Symbol;Acc:HGNC:48339] | chr2 | + |
| [23] | FOS | ENSG00000170345.10 | 4.544896 | 0 | Fos proto-oncogene, AP-1 transcription factor subunit [Source:HGNC Symbol;Acc:HGNC:3796] | chr14 | + |
| [24] | AC010307.3 | ENSG00000249478.1 | 4.5212 | 5.42E-21 | novel transcript | chr5 | + |
| [25] | SEMA3G | ENSG00000010319.7 | 4.48375 | 1.18E-12 | semaphorin 3G [Source:HGNC Symbol;Acc:HGNC:30400] | chr3 | - |
| [26] | FRG2C | ENSG00000172969.7 | 4.397368 | 7.33E-15 | FSHD region gene 2 family member C [Source:HGNC Symbol;Acc:HGNC:33626] | chr3 | + |
| [27] | CHAC1 | ENSG00000128965.13 | 4.391572 | 9.71E-153 | ChaC glutathione specific gamma-glutamylcyclotransferase 1 [Source:HGNC Symbol;Acc:HGNC:28680] | chr15 | + |
| [28] | TRIM69 | ENSG00000185880.13 | 4.360619 | 4.09E-11 | tripartite motif containing 69 [Source:HGNC Symbol;Acc:HGNC:17857] | chr15 | + |
| [29] | GADD45G | ENSG00000130222.11 | 4.350572 | 9.94E-12 | growth arrest and DNA damage inducible gamma [Source:HGNC Symbol;Acc:HGNC:4097] | chr9 | + |
| [30] | ATP1B2 | ENSG00000129244.9 | 4.330974 | 7.74E-23 | ATPase Na+/K+ transporting subunit beta 2 [Source:HGNC Symbol;Acc:HGNC:805] | chr17 | + |
| [31] | TRIM72 | ENSG00000177238.14 | 4.295404 | 3.23E-16 | tripartite motif containing 72 [Source:HGNC Symbol;Acc:HGNC:32671] | chr16 | + |
| [32] | RPL12P42 | ENSG00000213253.5 | 4.288776 | 1.78E-11 | ribosomal protein L12 pseudogene 42 [Source:HGNC Symbol;Acc:HGNC:36165] | chr19 | - |
| [33] | ZCCHC12 | ENSG00000174460.4 | 4.275539 | 2.24E-11 | zinc finger CCHC-type containing 12 [Source:HGNC Symbol;Acc:HGNC:27273] | chrX | + |
| [34] | AC068946.3 | ENSG00000287529.1 | 4.252821 | 3.31E-11 | novel transcript, antisense to ZFAND2B | chr2 | - |

| | | | | | | | |
|---|---|---|---|---|---|---|---|
| [35] | TMEM256-PLSCR3 | ENSG00000262481.5 | 4.24652 | 3.58E-23 | TMEM256-PLSCR3 readthrough (NMD candidate) [Source:HGNC Symbol;Acc:HGNC:49186] | chr17 | - |
| [36] | IGSF9 | ENSG00000085552.17 | 4.245213 | 3.28E-11 | immunoglobulin superfamily member 9 [Source:HGNC Symbol;Acc:HGNC:18132] | chr1 | - |
| [37] | LINC02158 | ENSG00000225611.1 | 4.226267 | 4.93E-11 | long intergenic non-protein coding RNA 2158 [Source:HGNC Symbol;Acc:HGNC:53019] | chr3 | + |
| [38] | IGF2 | ENSG00000167244.21 | 4.15105 | 1.29E-10 | insulin like growth factor 2 [Source:HGNC Symbol;Acc:HGNC:5466] | chr11 | - |
| [39] | SUN3 | ENSG00000164744.13 | 4.140504 | 2.24E-10 | Sad1 and UNC84 domain containing 3 [Source:HGNC Symbol;Acc:HGNC:22429] | chr7 | - |
| [40] | RND1 | ENSG00000172602.11 | 4.130541 | 7.32E-25 | Rho family GTPase 1 [Source:HGNC Symbol;Acc:HGNC:18314] | chr12 | - |
| [41] | USH1G | ENSG00000182040.9 | 4.095689 | 2.93E-10 | USH1 protein network component sans [Source:HGNC Symbol;Acc:HGNC:16356] | chr17 | - |
| [42] | GDF15 | ENSG00000130513.6 | 3.996778 | 0 | growth differentiation factor 15 [Source:HGNC Symbol;Acc:HGNC:30142] | chr19 | + |
| [43] | FOSB | ENSG00000125740.14 | 3.98363 | 1.33E-224 | FosB proto-oncogene, AP-1 transcription factor subunit [Source:HGNC Symbol;Acc:HGNC:3797] | chr19 | + |
| [44] | FUT1 | ENSG00000174951.11 | 3.978679 | 1.66E-10 | fucosyltransferase 1 (H blood group) [Source:HGNC Symbol;Acc:HGNC:4012] | chr19 | - |
| [45] | SPRR2D | ENSG00000163216.7 | 3.973165 | 3.66E-34 | small proline rich protein 2D [Source:HGNC Symbol;Acc:HGNC:11264] | chr1 | - |
| [46] | AC005532.1 | ENSG00000230825.2 | 3.94482 | 1.93E-09 | novel transcript | chr7 | + |
| [47] | AC087521.1 | ENSG00000244953.1 | 3.943737 | 1.98E-09 | novel transcript | chr11 | - |
| [48] | GNG7 | ENSG00000176533.13 | 3.935902 | 2.21E-09 | G protein subunit gamma 7 [Source:HGNC Symbol;Acc:HGNC:4410] | chr19 | - |
| [49] | AL356804.1 | ENSG00000259033.2 | 3.932982 | 2.30E-09 | novel transcript | chr14 | + |
| [50] | AL354872.2 | ENSG00000271992.1 | 3.909684 | 4.13E-10 | novel transcript | chr1 | + |
| [51] | DRAIC | ENSG00000245750.9 | 3.902544 | 3.81E-09 | downregulated RNA in cancer, inhibitor of cell invasion and migration [Source:HGNC Symbol;Acc:HGNC:27082] | chr15 | + |
| [52] | ZDHHC11B | ENSG00000206077.11 | 3.890776 | 8.37E-11 | zinc finger DHHC-type containing 11B [Source:HGNC Symbol;Acc:HGNC:32962] | chr5 | - |
| [53] | XIRP1 | ENSG00000168334.9 | 3.847332 | 6.79E-09 | xin actin binding repeat containing 1 [Source:HGNC Symbol;Acc:HGNC:14301] | chr3 | - |

| | | | | | | | |
|---|---|---|---|---|---|---|---|
| [54] | COL26A1 | ENSG00000160963.14 | 3.822978 | 9.52E-09 | collagen type XXVI alpha 1 chain [Source:HGNC Symbol;Acc:HGNC:18038] | chr7 | + |
| [55] | TRIB3 | ENSG00000101255.11 | 3.807516 | 0 | tribbles pseudokinase 3 [Source:HGNC Symbol;Acc:HGNC:16228] | chr20 | + |
| [56] | FAM71A | ENSG00000162771.8 | 3.801103 | 1.78E-09 | family with sequence similarity 71 member A [Source:HGNC Symbol;Acc:HGNC:26541] | chr1 | + |
| [57] | GADD45B | ENSG00000099860.9 | 3.786702 | 0 | growth arrest and DNA damage inducible beta [Source:HGNC Symbol;Acc:HGNC:4096] | chr19 | + |
| [58] | AL031590.1 | ENSG00000284633.1 | 3.782483 | 1.46E-08 | novel transcript, antisense to PDGFB | chr22 | + |
| [59] | SPANXN5 | ENSG00000204363.5 | 3.772884 | 1.84E-08 | SPANX family member N5 [Source:HGNC Symbol;Acc:HGNC:33178] | chrX | - |
| [60] | AC008060.1 | ENSG00000218672.1 | 3.76491 | 1.93E-08 | novel transcript | chr7 | + |
| [61] | DPEP2 | ENSG00000167261.14 | 3.727886 | 2.62E-08 | dipeptidase 2 [Source:HGNC Symbol;Acc:HGNC:23028] | chr16 | - |
| [62] | FAM71E2 | ENSG00000180043.11 | 3.7062 | 1.25E-09 | family with sequence similarity 71 member E2 [Source:HGNC Symbol;Acc:HGNC:25278] | chr19 | - |
| [63] | CTH | ENSG00000116761.12 | 3.689789 | 1.00E-171 | cystathionine gamma-lyase [Source:HGNC Symbol;Acc:HGNC:2501] | chr1 | + |
| [64] | RGMA | ENSG00000182175.14 | 3.687721 | 4.27E-12 | repulsive guidance molecule BMP co-receptor a [Source:HGNC Symbol;Acc:HGNC:30308] | chr15 | - |
| [65] | ULBP1 | ENSG00000111981.5 | 3.685598 | 1.44E-193 | UL16 binding protein 1 [Source:HGNC Symbol;Acc:HGNC:14893] | chr6 | + |
| [66] | DPPA2P4 | ENSG00000261429.2 | 3.670316 | 2.68E-11 | developmental pluripotency associated 2 pseudogene 4 [Source:HGNC Symbol;Acc:HGNC:44629] | chr16 | + |
| [67] | AC087203.2 | ENSG00000255556.2 | 3.658845 | 2.32E-09 | ubiquitin specific peptidase 17-like 2 (USP17L2) pseudogene | chr8 | - |
| [68] | CRYAB | ENSG00000109846.9 | 3.643301 | 3.94E-12 | crystallin alpha B [Source:HGNC Symbol;Acc:HGNC:2389] | chr11 | - |
| [69] | CALCB | ENSG00000175868.14 | 3.630166 | 4.07E-09 | calcitonin related polypeptide beta [Source:HGNC Symbol;Acc:HGNC:1438] | chr11 | + |
| [70] | EEF1AKMT4-ECE2 | ENSG00000284917.1 | 3.564778 | 1.55E-07 | EEF1AKMT4-ECE2 readthrough [Source:HGNC Symbol;Acc:HGNC:53615] | chr3 | + |
| [71] | AC004835.1 | ENSG00000230392.1 | 3.562897 | 7.86E-09 | novel transcript | chrX | + |
| [72] | ANKRD1 | ENSG00000148677.6 | 3.55128 | 7.28E-15 | ankyrin repeat domain 1 [Source:HGNC Symbol;Acc:HGNC:15819] | chr10 | - |

| | | | | | | | |
|---|---|---|---|---|---|---|---|
| [73] | UNC5B | ENSG00000107731.12 | 3.541083 | 0 | unc-5 netrin receptor B [Source:HGNC Symbol;Acc:HGNC:12568] | chr10 | + |
| [74] | MT1JP | ENSG00000255986.6 | 3.527006 | 2.28E-07 | metallothionein 1J, pseudogene [Source:HGNC Symbol;Acc:HGNC:7402] | chr16 | + |
| [75] | TTYH2 | ENSG00000141540.11 | 3.521148 | 2.44E-07 | tweety family member 2 [Source:HGNC Symbol;Acc:HGNC:13877] | chr17 | + |
| [76] | TCTE1 | ENSG00000146221.10 | 3.507395 | 2.85E-07 | t-complex-associated-testis-expressed 1 [Source:HGNC Symbol;Acc:HGNC:11693] | chr6 | - |
| [77] | SLC7A11 | ENSG00000151012.13 | 3.500562 | 0 | solute carrier family 7 member 11 [Source:HGNC Symbol;Acc:HGNC:11059] | chr4 | - |
| [78] | AC009336.2 | ENSG00000279160.1 | 3.488563 | 6.58E-09 | TEC | chr2 | - |
| [79] | USP17L7 | ENSG00000226430.6 | 3.488239 | 7.54E-10 | ubiquitin specific peptidase 17 like family member 7 [Source:HGNC Symbol;Acc:HGNC:37180] | chr8 | - |
| [80] | DDIT4 | ENSG00000168209.5 | 3.48771 | 0 | DNA damage inducible transcript 4 [Source:HGNC Symbol;Acc:HGNC:24944] | chr10 | + |
| [81] | PPP1R36 | ENSG00000165807.8 | 3.470223 | 2.15E-09 | protein phosphatase 1 regulatory subunit 36 [Source:HGNC Symbol;Acc:HGNC:20097] | chr14 | + |
| [82] | AC244453.2 | ENSG00000233029.3 | 3.446736 | 3.85E-08 | novel transcript | chr1 | - |
| [83] | PCDH10 | ENSG00000138650.9 | 3.445621 | 7.96E-93 | protocadherin 10 [Source:HGNC Symbol;Acc:HGNC:13404] | chr4 | + |
| [84] | ADM2 | ENSG00000128165.9 | 3.425809 | 8.70E-12 | adrenomedullin 2 [Source:HGNC Symbol;Acc:HGNC:28898] | chr22 | + |
| [85] | NR4A2 | ENSG00000153234.14 | 3.425524 | 1.91E-32 | nuclear receptor subfamily 4 group A member 2 [Source:HGNC Symbol;Acc:HGNC:7981] | chr2 | - |
| [86] | KLHL30-AS1 | ENSG00000279484.1 | 3.424713 | 1.58E-18 | KLHL30 antisense RNA 1 [Source:HGNC Symbol;Acc:HGNC:31018] | chr2 | - |
| [87] | AL136985.3 | ENSG00000283445.1 | 3.421542 | 2.29E-08 | novel transcript | chr1 | + |
| [88] | OBSCN | ENSG00000154358.21 | 3.421144 | 9.05E-09 | obscurin, cytoskeletal calmodulin and titin-interacting RhoGEF [Source:HGNC Symbol;Acc:HGNC:15719] | chr1 | + |
| [89] | IL17REL | ENSG00000188263.10 | 3.412165 | 7.76E-07 | interleukin 17 receptor E like [Source:HGNC Symbol;Acc:HGNC:33808] | chr22 | - |
| [90] | IL2RB | ENSG00000100385.14 | 3.399989 | 6.03E-08 | interleukin 2 receptor subunit beta [Source:HGNC Symbol;Acc:HGNC:6009] | chr22 | - |
| [91] | AC243772.2 | ENSG00000233030.2 | 3.391776 | 8.54E-09 | novel transcript, antisense to FCGR1A | chr1 | - |

| | | | | | | | |
|---|---|---|---|---|---|---|---|
| [92] | STPG3 | ENSG00000197768.10 | 3.374602 | 4.91E-10 | sperm-tail PG-rich repeat containing 3 [Source:HGNC Symbol;Acc:HGNC:37285] | chr9 | + |
| [93] | FCGR1A | ENSG00000150337.13 | 3.37236 | 1.07E-06 | Fc fragment of IgG receptor Ia [Source:HGNC Symbol;Acc:HGNC:3613] | chr1 | + |
| [94] | FGF18 | ENSG00000156427.8 | 3.338434 | 4.43E-14 | fibroblast growth factor 18 [Source:HGNC Symbol;Acc:HGNC:3674] | chr5 | + |
| [95] | DNAH2 | ENSG00000183914.14 | 3.334464 | 2.77E-13 | dynein axonemal heavy chain 2 [Source:HGNC Symbol;Acc:HGNC:2948] | chr17 | + |
| [96] | MYO15B | ENSG00000266714.9 | 3.3279 | 7.14E-11 | myosin XVB [Source:HGNC Symbol;Acc:HGNC:14083] | chr17 | + |
| [97] | LSMEM1 | ENSG00000181016.9 | 3.315882 | 1.11E-16 | leucine rich single-pass membrane protein 1 [Source:HGNC Symbol;Acc:HGNC:22036] | chr7 | + |
| [98] | ANKFN1 | ENSG00000153930.12 | 3.314569 | 1.03E-12 | ankyrin repeat and fibronectin type III domain containing 1 [Source:HGNC Symbol;Acc:HGNC:26766] | chr17 | + |
| [99] | LINC01978 | ENSG00000262188.2 | 3.30738 | 2.16E-06 | long intergenic non-protein coding RNA 1978 [Source:HGNC Symbol;Acc:HGNC:52806] | chr17 | + |
| [100] | RGS2 | ENSG00000116741.8 | 3.296927 | 3.14E-109 | regulator of G protein signaling 2 [Source:HGNC Symbol;Acc:HGNC:9998] | chr1 | + |
| [101] | LINC01705 | ENSG00000232679.2 | 3.296305 | 9.66E-19 | long intergenic non-protein coding RNA 1705 [Source:HGNC Symbol;Acc:HGNC:52493] | chr1 | - |
| [102] | ADAP2 | ENSG00000184060.11 | 3.291895 | 2.22E-06 | ArfGAP with dual PH domains 2 [Source:HGNC Symbol;Acc:HGNC:16487] | chr17 | + |
| [103] | BAHCC1 | ENSG00000266074.8 | 3.280479 | 6.74E-08 | BAH domain and coiled-coil containing 1 [Source:HGNC Symbol;Acc:HGNC:29279] | chr17 | + |
| [104] | AC087203.1 | ENSG00000254423.1 | 3.272256 | 2.57E-07 | ubiquitin specific peptidase 17-like 2 ( USP17L2) pseudogene | chr8 | - |
| [105] | TINCR | ENSG00000223573.7 | 3.255755 | 1.06E-06 | TINCR ubiquitin domain containing [Source:HGNC Symbol;Acc:HGNC:14607] | chr19 | - |
| [106] | ATCAY | ENSG00000167654.18 | 3.230318 | 4.04E-06 | ATCAY kinesin light chain interacting caytaxin [Source:HGNC Symbol;Acc:HGNC:779] | chr19 | + |
| [107] | PCDH1 | ENSG00000156453.13 | 3.213897 | 6.94E-07 | protocadherin 1 [Source:HGNC Symbol;Acc:HGNC:8655] | chr5 | - |
| [108] | VN2R3P | ENSG00000234680.1 | 3.201704 | 5.08E-06 | vomeronasal 2 receptor 3, pseudogene [Source:HGNC Symbol;Acc:HGNC:33209] | chr9 | + |
| [109] | TATDN2P3 | ENSG00000235205.1 | 3.1742 | 1.09E-06 | TatD DNase domain containing 2 pseudogene 3 | chr13 | + |

| | | | | | | | |
|---|---|---|---|---|---|---|---|
| | | | | | [Source:HGNC Symbol;Acc:HGNC:39256] | | |
| [110] | MOV10L1 | ENSG00000073146.16 | 3.17417 | 1.50E-06 | Mov10 like RISC complex RNA helicase 1 [Source:HGNC Symbol;Acc:HGNC:7201] | chr22 | + |
| [111] | CD79A | ENSG00000105369.9 | 3.17112 | 6.49E-06 | CD79a molecule [Source:HGNC Symbol;Acc:HGNC:1698] | chr19 | + |
| [112] | DNAH3 | ENSG00000158486.13 | 3.162367 | 3.05E-07 | dynein axonemal heavy chain 3 [Source:HGNC Symbol;Acc:HGNC:2949] | chr16 | - |
| [113] | DACH1 | ENSG00000276644.5 | 3.159199 | 4.17E-20 | dachshund family transcription factor 1 [Source:HGNC Symbol;Acc:HGNC:2663] | chr13 | - |
| [114] | RPLP0P2 | ENSG00000243742.5 | 3.143877 | 8.29E-81 | ribosomal protein lateral stalk subunit P0 pseudogene 2 [Source:HGNC Symbol;Acc:HGNC:17960] | chr11 | + |
| [115] | AL022326.1 | ENSG00000224794.2 | 3.13394 | 9.93E-06 | novel transcript | chr22 | + |
| [116] | SLIT2 | ENSG00000145147.20 | 3.128024 | 4.00E-64 | slit guidance ligand 2 [Source:HGNC Symbol;Acc:HGNC:11086] | chr4 | + |
| [117] | VTCN1 | ENSG00000134258.17 | 3.120048 | 1.03E-05 | V-set domain containing T cell activation inhibitor 1 [Source:HGNC Symbol;Acc:HGNC:28873] | chr1 | - |
| [118] | RSAD2 | ENSG00000134321.12 | 3.106324 | 1.16E-05 | radical S-adenosyl methionine domain containing 2 [Source:HGNC Symbol;Acc:HGNC:30908] | chr2 | + |
| [119] | HIPK4 | ENSG00000160396.9 | 3.105017 | 1.08E-06 | homeodomain interacting protein kinase 4 [Source:HGNC Symbol;Acc:HGNC:19007] | chr19 | - |
| [120] | LINC01977 | ENSG00000262772.2 | 3.094091 | 1.33E-05 | long intergenic non-protein coding RNA 1977 [Source:HGNC Symbol;Acc:HGNC:52804] | chr17 | + |
| [121] | ARC | ENSG00000198576.4 | 3.091599 | 0 | activity regulated cytoskeleton associated protein [Source:HGNC Symbol;Acc:HGNC:648] | chr8 | - |
| [122] | AC104825.1 | ENSG00000251615.3 | 3.089389 | 2.11E-08 | novel transcript | chr4 | - |
| [123] | ZPLD1 | ENSG00000170044.8 | 3.077843 | 1.48E-05 | zona pellucida like domain containing 1 [Source:HGNC Symbol;Acc:HGNC:27022] | chr3 | + |
| [124] | AC011462.1 | ENSG00000255730.5 | 3.070303 | 1.84E-05 | novel protein | chr19 | + |
| [125] | N4BP3 | ENSG00000145911.6 | 3.048438 | 1.84E-05 | NEDD4 binding protein 3 [Source:HGNC Symbol;Acc:HGNC:29852] | chr5 | + |
| [126] | AC007317.1 | ENSG00000285898.1 | 3.046239 | 1.94E-05 | Novel transcript | chr2 | + |
| [127] | STPG3-AS1 | ENSG00000275549.1 | 3.045982 | 6.88E-13 | STPG3 antisense RNA 1 [Source:HGNC Symbol;Acc:HGNC:51176] | chr9 | - |

| | | | | | | | |
|---|---|---|---|---|---|---|---|
| [128] | SLC7A11-AS1 | ENSG00000250033.5 | 3.044937 | 1.05E-13 | SLC7A11 antisense RNA 1 [Source:HGNC Symbol;Acc:HGNC:44064] | chr4 | + |
| [129] | FLG-AS1 | ENSG00000237975.7 | 3.041116 | 3.39E-21 | FLG antisense RNA 1 [Source:HGNC Symbol;Acc:HGNC:27913] | chr1 | + |
| [130] | PEAK3 | ENSG00000188305.6 | 3.040021 | 6.28E-10 | PEAK family member 3 [Source:HGNC Symbol;Acc:HGNC:24793] | chr19 | - |
| [131] | AC010378.2 | ENSG00000286403.1 | 3.023012 | 4.91E-06 | novel transcript | chr5 | + |
| [132] | HAVCR2 | ENSG00000135077.9 | 3.021262 | 2.24E-05 | hepatitis A virus cellular receptor 2 [Source:HGNC Symbol;Acc:HGNC:18437] | chr5 | - |
| [133] | LINC01979 | ENSG00000262585.1 | 3.021262 | 2.24E-05 | long intergenic non-protein coding RNA 1979 [Source:HGNC Symbol;Acc:HGNC:52807] | chr17 | - |
| [134] | AC105046.1 | ENSG00000261026.1 | 3.015918 | 2.41E-05 | novel transcript, overlapping to EGR3 | chr8 | - |
| [135] | FMOD | ENSG00000122176.12 | 3.00828 | 2.49E-05 | fibromodulin [Source:HGNC Symbol;Acc:HGNC:3774] | chr1 | - |
| [136] | KLF4 | ENSG00000136826.15 | 3.007449 | 3.10E-93 | Kruppel like factor 4 [Source:HGNC Symbol;Acc:HGNC:6348] | chr9 | - |
| [137] | KRTAP21-2 | ENSG00000187026.3 | 3.006379 | 2.53E-05 | keratin associated protein 21-2 [Source:HGNC Symbol;Acc:HGNC:18946] | chr21 | - |
| [138] | CSPG5 | ENSG00000114646.10 | 3.005571 | 2.55E-05 | chondroitin sulfate proteoglycan 5 [Source:HGNC Symbol;Acc:HGNC:2467] | chr3 | - |
| [139] | GULP1 | ENSG00000144366.16 | 3.002643 | 1.80E-257 | GULP PTB domain containing engulfment adaptor 1 [Source:HGNC Symbol;Acc:HGNC:18649] | chr2 | + |
| [140] | FP236383.1 | ENSG00000274868.1 | 2.995039 | 4.59E-06 | nan | chr21 | + |
| [141] | FP671120.1 | ENSG00000275664.1 | 2.995039 | 4.59E-06 | nan | chr21 | + |
| [142] | FP671120.3 | ENSG00000277671.1 | 2.995039 | 4.59E-06 | nan | chr21 | + |
| [143] | FP236383.2 | ENSG00000278775.1 | 2.995039 | 4.59E-06 | nan | chr21 | + |
| [144] | CLDN1 | ENSG00000163347.6 | 2.990274 | 2.87E-06 | claudin 1 [Source:HGNC Symbol;Acc:HGNC:2032] | chr3 | - |
| [145] | LINC00881 | ENSG00000241135.6 | 2.988584 | 2.88E-06 | long intergenic non-protein coding RNA 881 [Source:HGNC Symbol;Acc:HGNC:48567] | chr3 | + |
| [146] | TENT5C | ENSG00000183508.5 | 2.971829 | 9.44E-47 | terminal nucleotidyltransferase 5C [Source:HGNC Symbol;Acc:HGNC:24712] | chr1 | + |
| [147] | C5AR1 | ENSG00000197405.8 | 2.963257 | 1.11E-41 | complement C5a receptor 1 [Source:HGNC Symbol;Acc:HGNC:1338] | chr19 | + |
| [148] | SENP3-EIF4A1 | ENSG00000277957.1 | 2.960422 | 1.03E-08 | SENP3-EIF4A1 readthrough (NMD candidate) [Source:HGNC Symbol;Acc:HGNC:49182] | chr17 | + |

| | | | | | | | |
|---|---|---|---|---|---|---|---|
| [149] | SERPINB10 | ENSG00000242550.6 | 2.94732 | 1.08E-05 | serpin family B member 10 [Source:HGNC Symbol;Acc:HGNC:8942] | chr18 | + |
| [150] | CRISPLD2 | ENSG00000103196.12 | 2.943871 | 2.79E-20 | cysteine rich secretory protein LCCL domain containing 2 [Source:HGNC Symbol;Acc:HGNC:25248] | chr16 | + |
| [151] | AC022217.2 | ENSG00000253295.2 | 2.942618 | 4.85E-06 | novel transcript | chr5 | + |
| [152] | ADGRD1 | ENSG00000111452.13 | 2.941348 | 4.13E-06 | adhesion G protein-coupled receptor D1 [Source:HGNC Symbol;Acc:HGNC:19893] | chr12 | + |
| [153] | UNC13D | ENSG00000092929.11 | 2.940452 | 8.63E-08 | unc-13 homoLog D [Source:HGNC Symbol;Acc:HGNC:23147] | chr17 | - |
| [154] | MAFB | ENSG00000204103.4 | 2.934381 | 1.34E-90 | MAF bZIP transcription factor B [Source:HGNC Symbol;Acc:HGNC:6408] | chr20 | - |
| [155] | PCK1 | ENSG00000124253.11 | 2.92488 | 4.72E-05 | phosphoenolpyruvate carboxykinase 1 [Source:HGNC Symbol;Acc:HGNC:8724] | chr20 | + |
| [156] | ZNF606 | ENSG00000166704.11 | 2.923542 | 2.42E-05 | zinc finger protein 606 [Source:HGNC Symbol;Acc:HGNC:25879] | chr19 | - |
| [157] | LINC00622 | ENSG00000260941.1 | 2.91903 | 1.78E-28 | long intergenic non-protein coding RNA 622 [Source:HGNC Symbol;Acc:HGNC:44251] | chr1 | - |
| [158] | AL031602.2 | ENSG00000287691.1 | 2.9155 | 5.06E-05 | novel transcript, antisense to RNF19B | chr1 | + |
| [159] | AL157388.1 | ENSG00000229272.2 | 2.913517 | 5.26E-05 | novel transcript | chr10 | - |
| [160] | AC108517.2 | ENSG00000286097.1 | 2.910364 | 4.93E-07 | novel transcript | chr4 | - |
| [161] | SPOCK2 | ENSG00000107742.13 | 2.906413 | 5.29E-05 | SPARC (osteonectin), cwcv and kazal like domains proteoglycan 2 [Source:HGNC Symbol;Acc:HGNC:13564] | chr10 | - |
| [162] | NMRAL2P | ENSG00000171658.8 | 2.894806 | 3.47E-11 | NmrA like redox sensor 2, pseudogene [Source:HGNC Symbol;Acc:HGNC:52332] | chr3 | + |
| [163] | SESN2 | ENSG00000130766.5 | 2.884742 | 0 | sestrin 2 [Source:HGNC Symbol;Acc:HGNC:20746] | chr1 | + |
| [164] | CYP4F3 | ENSG00000186529.16 | 2.876757 | 1.69E-05 | cytochrome P450 family 4 subfamily F member 3 [Source:HGNC Symbol;Acc:HGNC:2646] | chr19 | + |
| [165] | LINC00334 | ENSG00000182586.9 | 2.863991 | 7.32E-05 | long intergenic non-protein coding RNA 334 [Source:HGNC Symbol;Acc:HGNC:16425] | chr21 | + |
| [166] | AP003721.4 | ENSG00000257052.1 | 2.863991 | 7.32E-05 | novel transcript | chr11 | + |
| [167] | TCP11L2 | ENSG00000166046.11 | 2.849936 | 2.36E-24 | t-complex 11 like 2 [Source:HGNC Symbol;Acc:HGNC:28627] | chr12 | + |
| [168] | NATD1 | ENSG00000274180.1 | 2.842175 | 3.46E-31 | N-acetyltransferase domain containing 1 [Source:HGNC Symbol;Acc:HGNC:30770] | chr17 | - |

| | | | | | | | |
|---|---|---|---|---|---|---|---|
| [169] | SOX9-AS1 | ENSG00000234899.10 | 2.838408 | 6.10E-05 | SOX9 antisense RNA 1 [Source:HGNC Symbol;Acc:HGNC:49321] | chr17 | - |
| [170] | CEMP1 | ENSG00000205923.3 | 2.837033 | 0.000107 | cementum protein 1 [Source:HGNC Symbol;Acc:HGNC:32553] | chr16 | - |
| [171] | FOXJ1 | ENSG00000129654.8 | 2.835279 | 9.04E-05 | forkhead box J1 [Source:HGNC Symbol;Acc:HGNC:3816] | chr17 | - |
| [172] | MGAT4A | ENSG00000071073.13 | 2.831889 | 1.54E-05 | alpha-1,3-mannosyl-glycoprotein 4-beta-N-acetylglucosaminyltransferase A [Source:HGNC Symbol;Acc:HGNC:7047] | chr2 | - |
| [173] | NUPR1 | ENSG00000176046.8 | 2.831097 | 1.06E-280 | nuclear protein 1, transcriptional regulator [Source:HGNC Symbol;Acc:HGNC:29990] | chr16 | - |
| [174] | MIR191 | ENSG00000207605.3 | 2.811346 | 0.000105 | microRNA 191 [Source:HGNC Symbol;Acc:HGNC:31561] | chr3 | - |
| [175] | CLDN19 | ENSG00000164007.11 | 2.810283 | 0.000106 | claudin 19 [Source:HGNC Symbol;Acc:HGNC:2040] | chr1 | - |
| [176] | ZNF114 | ENSG00000178150.10 | 2.808349 | 0.000107 | zinc finger protein 114 [Source:HGNC Symbol;Acc:HGNC:12894] | chr19 | + |
| [177] | ATF3 | ENSG00000162772.17 | 2.807166 | 2.46E-180 | activating transcription factor 3 [Source:HGNC Symbol;Acc:HGNC:785] | chr1 | + |
| [178] | RNF103-CHMP3 | ENSG00000249884.8 | 2.803409 | 2.19E-05 | RNF103-CHMP3 readthrough [Source:HGNC Symbol;Acc:HGNC:38847] | chr2 | - |
| [179] | ESRP1 | ENSG00000104413.18 | 2.802984 | 1.14E-05 | epithelial splicing regulatory protein 1 [Source:HGNC Symbol;Acc:HGNC:25966] | chr8 | + |
| [180] | MYADML2 | ENSG00000185105.5 | 2.802119 | 3.48E-05 | myeloid associated differentiation marker like 2 [Source:HGNC Symbol;Acc:HGNC:34548] | chr17 | - |
| [181] | PIP5K1B | ENSG00000107242.19 | 2.796258 | 6.75E-05 | phosphatidylinositol-4-phosphate 5-kinase type 1 beta [Source:HGNC Symbol;Acc:HGNC:8995] | chr9 | + |
| [182] | C3orf85 | ENSG00000241224.8 | 2.792024 | 1.56E-14 | chromosome 3 open reading frame 85 [Source:HGNC Symbol;Acc:HGNC:53432] | chr3 | + |
| [183] | AL160269.1 | ENSG00000285269.2 | 2.786214 | 0.00015 | novel transcript, SLC35D2-HSD17B3 readthrough | chr9 | - |
| [184] | WDR49 | ENSG00000174776.11 | 2.784164 | 0.000148 | WD repeat domain 49 [Source:HGNC Symbol;Acc:HGNC:26587] | chr3 | - |
| [185] | ISY1-RAB43 | ENSG00000261796.1 | 2.778204 | 4.02E-51 | ISY1-RAB43 readthrough [Source:HGNC Symbol;Acc:HGNC:42969] | chr3 | - |
| [186] | AC245100.3 | ENSG00000228626.1 | 2.770193 | 0.000141 | novel pseudogene | chr1 | - |
| [187] | PIWIL2 | ENSG00000197181.12 | 2.76999 | 2.19E-05 | piwi like RNA-mediated gene silencing 2 [Source:HGNC Symbol;Acc:HGNC:17644] | chr8 | + |

| | | | | | | | |
|---|---|---|---|---|---|---|---|
| [188] | AL157935.1 | ENSG00000227218.7 | 2.762698 | 3.95E-24 | novel transcript | chr9 | + |
| [189] | OVGP1 | ENSG00000085465.13 | 2.758537 | 1.31E-06 | oviductal glycoprotein 1 [Source:HGNC Symbol;Acc:HGNC:8524] | chr1 | - |
| [190] | KLHL24 | ENSG00000114796.16 | 2.751849 | 1.19E-233 | kelch like family member 24 [Source:HGNC Symbol;Acc:HGNC:25947] | chr3 | + |
| [191] | MSH4 | ENSG00000057468.7 | 2.743112 | 0.000189 | mutS homoLog 4 [Source:HGNC Symbol;Acc:HGNC:7327] | chr1 | + |
| [192] | BX470209.2 | ENSG00000236507.2 | 2.737528 | 0.000174 | novel transcript | chr9 | - |
| [193] | AC087428.1 | ENSG00000287505.1 | 2.737504 | 0.000176 | novel transcript | chr3 | - |
| [194] | AC010336.1 | ENSG00000214248.2 | 2.73735 | 2.60E-05 | novel transcript, antisense to LRRC8E | chr19 | - |
| [195] | PDGFB | ENSG00000100311.17 | 2.737338 | 0.000174 | platelet derived growth factor subunit B [Source:HGNC Symbol;Acc:HGNC:8800] | chr22 | - |
| [196] | TLR9 | ENSG00000239732.3 | 2.737338 | 0.000174 | toll like receptor 9 [Source:HGNC Symbol;Acc:HGNC:15633] | chr3 | - |
| [197] | AC009108.3 | ENSG00000270020.1 | 2.737295 | 0.000177 | novel transcript | chr16 | - |
| [198] | CCR2 | ENSG00000121807.5 | 2.736433 | 0.000178 | C-C motif chemokine receptor 2 [Source:HGNC Symbol;Acc:HGNC:1603] | chr3 | + |
| [199] | MYO1G | ENSG00000136286.16 | 2.736394 | 0.000175 | myosin IG [Source:HGNC Symbol;Acc:HGNC:13880] | chr7 | - |
| [200] | AL138828.1 | ENSG00000237596.7 | 2.736187 | 1.94E-23 | novel transcript | chr6 | - |
| [201] | OLAH | ENSG00000152463.14 | 2.732731 | 7.53E-06 | oleoyl-ACP hydrolase [Source:HGNC Symbol;Acc:HGNC:25625] | chr10 | + |
| [202] | BMF | ENSG00000104081.14 | 2.715476 | 1.29E-32 | Bcl2 modifying factor [Source:HGNC Symbol;Acc:HGNC:24132] | chr15 | - |
| [203] | TG | ENSG00000042832.12 | 2.713046 | 6.67E-06 | thyroglobulin [Source:HGNC Symbol;Acc:HGNC:11764] | chr8 | + |
| [204] | AC022868.2 | ENSG00000286113.1 | 2.709742 | 5.20E-05 | long intergenic non-protein coding RNA 1617 | chr8 | + |
| [205] | CD14 | ENSG00000170458.14 | 2.707562 | 2.12E-05 | CD14 molecule [Source:HGNC Symbol;Acc:HGNC:1628] | chr5 | - |
| [206] | DHRS2 | ENSG00000100867.14 | 2.707123 | 5.23E-31 | dehydrogenase/reductase 2 [Source:HGNC Symbol;Acc:HGNC:18349] | chr14 | + |
| [207] | IQCN | ENSG00000130518.17 | 2.699402 | 7.83E-48 | IQ motif containing N [Source:HGNC Symbol;Acc:HGNC:29350] | chr19 | - |
| [208] | AGT | ENSG00000135744.8 | 2.697159 | 9.31E-05 | angiotensinogen [Source:HGNC Symbol;Acc:HGNC:333] | chr1 | - |
| [209] | CNTN4 | ENSG00000144619.15 | 2.695478 | 0.000158 | contactin 4 [Source:HGNC Symbol;Acc:HGNC:2174] | chr3 | + |
| [210] | EGLN3 | ENSG00000129521.14 | 2.68587 | 5.88E-07 | egl-9 family hypoxia inducible factor 3 [Source:HGNC Symbol;Acc:HGNC:14661] | chr14 | - |
| [211] | AL137077.2 | ENSG00000283078.1 | 2.684352 | 6.41E-11 | novel transcript | chr20 | + |

| | | | | | | | |
|---|---|---|---|---|---|---|---|
| [212] | RRH | ENSG00000180245.6 | 2.677651 | 0.00026 | retinal pigment epithelium-derived rhodopsin homoLog [Source:HGNC Symbol;Acc:HGNC:10450] | chr4 | + |
| [213] | AC068506.1 | ENSG00000278464.2 | 2.675931 | 0.000104 | novel transcript | chr18 | - |
| [214] | HRNR | ENSG00000197915.6 | 2.675867 | 4.06E-129 | hornerin [Source:HGNC Symbol;Acc:HGNC:20846] | chr1 | - |
| [215] | ZNF177 | ENSG00000188629.12 | 2.669696 | 0.000287 | zinc finger protein 177 [Source:HGNC Symbol;Acc:HGNC:12966] | chr19 | + |
| [216] | PKMP3 | ENSG00000220563.1 | 2.666462 | 1.95E-05 | pyruvate kinase M1/2 pseudogene 3 [Source:HGNC Symbol;Acc:HGNC:44245] | chr6 | - |
| [217] | KLHDC7B | ENSG00000130487.9 | 2.653156 | 4.86E-07 | kelch domain containing 7B [Source:HGNC Symbol;Acc:HGNC:25145] | chr22 | + |
| [218] | NGF-AS1 | ENSG00000228035.1 | 2.637768 | 0.000336 | NGF antisense RNA 1 [Source:HGNC Symbol;Acc:HGNC:53922] | chr1 | + |
| [219] | AQP3 | ENSG00000165272.16 | 2.631131 | 6.94E-05 | aquaporin 3 (Gill blood group) [Source:HGNC Symbol;Acc:HGNC:636] | chr9 | - |
| [220] | KDM7A | ENSG00000006459.11 | 2.630778 | 2.49E-108 | lysine demethylase 7A [Source:HGNC Symbol;Acc:HGNC:22224] | chr7 | - |
| [221] | AOC3 | ENSG00000131471.7 | 2.629999 | 1.16E-09 | amine oxidase copper containing 3 [Source:HGNC Symbol;Acc:HGNC:550] | chr17 | + |
| [222] | GSTM3 | ENSG00000134202.11 | 2.627311 | 0.000146 | glutathione S-transferase mu 3 [Source:HGNC Symbol;Acc:HGNC:4635] | chr1 | - |
| [223] | PNRC1 | ENSG00000146278.11 | 2.618825 | 6.33E-151 | proline rich nuclear receptor coactivator 1 [Source:HGNC Symbol;Acc:HGNC:17278] | chr6 | + |
| [224] | DDIT3 | ENSG00000175197.12 | 2.616806 | 0 | DNA damage inducible transcript 3 [Source:HGNC Symbol;Acc:HGNC:2726] | chr12 | - |
| [225] | CREBRF | ENSG00000164463.12 | 2.6092 | 1.17E-173 | CREB3 regulatory factor [Source:HGNC Symbol;Acc:HGNC:24050] | chr5 | + |
| [226] | PRTG | ENSG00000166450.13 | 2.606044 | 0.000405 | protogenin [Source:HGNC Symbol;Acc:HGNC:26373] | chr15 | - |
| [227] | DISP3 | ENSG00000204624.8 | 2.605127 | 0.000408 | dispatched RND transporter family member 3 [Source:HGNC Symbol;Acc:HGNC:29251] | chr1 | + |
| [228] | HNF4A | ENSG00000101076.16 | 2.602077 | 0.00017 | hepatocyte nuclear factor 4 alpha [Source:HGNC Symbol;Acc:HGNC:5024] | chr20 | + |
| [229] | DIRAS2 | ENSG00000165023.7 | 2.592641 | 6.12E-09 | DIRAS family GTPase 2 [Source:HGNC Symbol;Acc:HGNC:19323] | chr9 | - |
| [230] | FNDC7 | ENSG00000143107.10 | 2.587827 | 1.19E-05 | fibronectin type III domain containing 7 [Source:HGNC Symbol;Acc:HGNC:26668] | chr1 | + |
| [231] | ACAN | ENSG00000157766.17 | 2.58469 | 0.000464 | aggrecan [Source:HGNC Symbol;Acc:HGNC:319] | chr15 | + |

| | | | | | | | |
|---|---|---|---|---|---|---|---|
| [232] | AC016717.2 | ENSG00000273301.1 | 2.584604 | 3.01E-05 | novel transcript | chr2 | + |
| [233] | EAF1-AS1 | ENSG00000249786.7 | 2.584376 | 1.46E-05 | EAF1 antisense RNA 1 [Source:HGNC Symbol;Acc:HGNC:42328] | chr3 | - |
| [234] | MRC1 | ENSG00000260314.3 | 2.584291 | 3.02E-05 | mannose receptor C-type 1 [Source:HGNC Symbol;Acc:HGNC:7228] | chr10 | + |
| [235] | ZFHX2 | ENSG00000136367.14 | 2.583394 | 0.000122 | zinc finger homeobox 2 [Source:HGNC Symbol;Acc:HGNC:20152] | chr14 | - |
| [236] | SHANK2 | ENSG00000162105.19 | 2.579888 | 1.50E-05 | SH3 and multiple ankyrin repeat domains 2 [Source:HGNC Symbol;Acc:HGNC:14295] | chr11 | - |
| [237] | AC006538.1 | ENSG00000261342.1 | 2.578608 | 4.34E-17 | novel transcript | chr19 | - |
| [238] | KCNJ3 | ENSG00000162989.5 | 2.574115 | 0.000152 | potassium inwardly rectifying channel subfamily J member 3 [Source:HGNC Symbol;Acc:HGNC:6264] | chr2 | + |
| [239] | BAMBI | ENSG00000095739.11 | 2.572906 | 5.18E-16 | BMP and activin membrane bound inhibitor [Source:HGNC Symbol;Acc:HGNC:30251] | chr10 | + |
| [240] | ID2 | ENSG00000115738.10 | 2.559267 | 0 | inhibitor of DNA binding 2 [Source:HGNC Symbol;Acc:HGNC:5361] | chr2 | + |
| [241] | C1orf61 | ENSG00000125462.18 | 2.554901 | 2.36E-07 | MIR9-1 host gene [Source:HGNC Symbol;Acc:HGNC:30780] | chr1 | - |
| [242] | TUSC3 | ENSG00000104723.21 | 2.549187 | 2.57E-05 | tumor suppressor candidate 3 [Source:HGNC Symbol;Acc:HGNC:30242] | chr8 | + |
| [243] | COL20A1 | ENSG00000101203.16 | 2.545362 | 0.00021 | collagen type XX alpha 1 chain [Source:HGNC Symbol;Acc:HGNC:14670] | chr20 | + |
| [244] | RPL3L | ENSG00000140986.8 | 2.54158 | 0.000622 | ribosomal protein L3 like [Source:HGNC Symbol;Acc:HGNC:10351] | chr16 | - |
| [245] | RTL5 | ENSG00000242732.4 | 2.539789 | 8.37E-11 | retrotransposon Gag like 5 [Source:HGNC Symbol;Acc:HGNC:29430] | chrX | - |
| [246] | AKNA | ENSG00000106948.16 | 2.537497 | 3.32E-42 | AT-hook transcription factor [Source:HGNC Symbol;Acc:HGNC:24108] | chr9 | - |
| [247] | KRTAP13-2 | ENSG00000182816.9 | 2.535435 | 0.000619 | keratin associated protein 13-2 [Source:HGNC Symbol;Acc:HGNC:18923] | chr21 | - |
| [248] | S1PR1 | ENSG00000170989.9 | 2.533316 | 5.88E-09 | sphingosine-1-phosphate receptor 1 [Source:HGNC Symbol;Acc:HGNC:3165] | chr1 | + |
| [249] | AL606500.1 | ENSG00000287064.1 | 2.532006 | 5.01E-10 | novel transcript | chr1 | + |
| [250] | AC125257.1 | ENSG00000259623.1 | 2.528314 | 0.00023 | novel transcript, sense overlapping to KLHL11 | chr17 | - |
| [251] | ERBB3 | ENSG00000065361.15 | 2.523134 | 3.00E-05 | erb-b2 receptor tyrosine kinase 3 [Source:HGNC Symbol;Acc:HGNC:3431] | chr12 | + |

| | | | | | | | |
|---|---|---|---|---|---|---|---|
| [252] | TRIM54 | ENSG00000138100.13 | 2.516766 | 2.53E-05 | tripartite motif containing 54 [Source:HGNC Symbol;Acc:HGNC:16008] | chr2 | + |
| [253] | PLCL1 | ENSG00000115896.16 | 2.515448 | 2.34E-06 | phospholipase C like 1 (inactive) [Source:HGNC Symbol;Acc:HGNC:9063] | chr2 | + |
| [254] | MYCT1 | ENSG00000120279.6 | 2.5123 | 0.00071 | MYC target 1 [Source:HGNC Symbol;Acc:HGNC:23172] | chr6 | + |
| [255] | MORC1 | ENSG00000114487.10 | 2.509442 | 4.23E-32 | MORC family CW-type zinc finger 1 [Source:HGNC Symbol;Acc:HGNC:7198] | chr3 | - |
| [256] | AC144831.1 | ENSG00000261888.1 | 2.509218 | 7.55E-05 | novel transcript | chr17 | + |
| [257] | AL139130.1 | ENSG00000237390.1 | 2.49687 | 0.000355 | novel transcript | chr1 | + |
| [258] | CDKL3 | ENSG00000006837.12 | 2.493738 | 1.58E-05 | cyclin dependent kinase like 3 [Source:HGNC Symbol;Acc:HGNC:15483] | chr5 | - |
| [259] | SLC6A9 | ENSG00000196517.11 | 2.483774 | 4.33E-46 | solute carrier family 6 member 9 [Source:HGNC Symbol;Acc:HGNC:11056] | chr1 | - |
| [260] | CD163 | ENSG00000177575.12 | 2.482114 | 0.0008 | CD163 molecule [Source:HGNC Symbol;Acc:HGNC:1631] | chr12 | - |
| [261] | LINC00917 | ENSG00000168367.11 | 2.480641 | 8.97E-05 | long intergenic non-protein coding RNA 917 [Source:HGNC Symbol;Acc:HGNC:48607] | chr16 | - |
| [262] | NRCAM | ENSG00000091129.20 | 2.480298 | 3.47E-05 | neuronal cell adhesion molecule [Source:HGNC Symbol;Acc:HGNC:7994] | chr7 | - |
| [263] | AC051619.5 | ENSG00000259520.6 | 2.474166 | 0.000879 | novel transcript, antisense to SLC28A2 | chr15 | - |
| [264] | DYDC2 | ENSG00000133665.13 | 2.471525 | 0.000486 | DPY30 domain containing 2 [Source:HGNC Symbol;Acc:HGNC:23468] | chr10 | + |
| [265] | MAB21L3 | ENSG00000173212.4 | 2.47132 | 4.58E-07 | mab-21 like 3 [Source:HGNC Symbol;Acc:HGNC:26787] | chr1 | + |
| [266] | ATP6V0D2 | ENSG00000147614.4 | 2.46874 | 3.86E-97 | ATPase H+ transporting V0 subunit d2 [Source:HGNC Symbol;Acc:HGNC:18266] | chr8 | + |
| [267] | ADAMTSL4 | ENSG00000143382.14 | 2.464102 | 1.62E-10 | ADAMTS like 4 [Source:HGNC Symbol;Acc:HGNC:19706] | chr1 | + |
| [268] | MRNIP | ENSG00000161010.15 | 2.460702 | 2.65E-09 | MRN complex interacting protein [Source:HGNC Symbol;Acc:HGNC:30817] | chr5 | - |
| [269] | MYLIP | ENSG00000007944.15 | 2.459974 | 1.47E-22 | myosin regulatory light chain interacting protein [Source:HGNC Symbol;Acc:HGNC:21155] | chr6 | + |
| [270] | AC004453.2 | ENSG00000279853.1 | 2.453484 | 0.00078 | tec | chr7 | - |
| [271] | IDH1 | ENSG00000138413.13 | 2.451229 | 0 | isocitrate dehydrogenase (NADP(+)) 1 [Source:HGNC Symbol;Acc:HGNC:5382] | chr2 | - |
| [272] | TEX54 | ENSG00000283268.1 | 2.450338 | 9.76E-05 | testis expressed 54 [Source:HGNC Symbol;Acc:HGNC:53729] | chr11 | - |

| | | | | | | | |
|---|---|---|---|---|---|---|---|
| [273] | TCL6 | ENSG00000187621.14 | 2.448647 | 1.66E-09 | T cell leukemia/lymphoma 6 [Source:HGNC Symbol;Acc:HGNC:13463] | chr14 | + |
| [274] | MUC17 | ENSG00000169876.14 | 2.439946 | 0.00107 | mucin 17, cell surface associated [Source:HGNC Symbol;Acc:HGNC:16800] | chr7 | + |
| [275] | CPNE5 | ENSG00000124772.12 | 2.439676 | 0.001071 | copine 5 [Source:HGNC Symbol;Acc:HGNC:2318] | chr6 | - |
| [276] | ADAM30 | ENSG00000134249.7 | 2.439454 | 0.001072 | ADAM metallopeptidase domain 30 [Source:HGNC Symbol;Acc:HGNC:208] | chr1 | - |
| [277] | WFIKKN2 | ENSG00000173714.8 | 2.439454 | 0.001072 | WAP, follistatin/kazal, immunoglobulin, kunitz and netrin domain containing 2 [Source:HGNC Symbol;Acc:HGNC:30916] | chr17 | + |
| [278] | MIR7-3HG | ENSG00000176840.12 | 2.438609 | 0.001077 | MIR7-3 host gene [Source:HGNC Symbol;Acc:HGNC:30049] | chr19 | + |
| [279] | TBC1D29P | ENSG00000266733.7 | 2.438609 | 0.001077 | TBC1 domain family member 29, pseudogene [Source:HGNC Symbol;Acc:HGNC:24509] | chr17 | + |
| [280] | AC005393.1 | ENSG00000276445.1 | 2.435954 | 0.000188 | novel transcript | chr19 | - |
| [281] | AC027644.4 | ENSG00000284461.2 | 2.434045 | 0.000535 | novel transcript | chr7 | + |
| [282] | BTG2 | ENSG00000159388.6 | 2.424563 | 1.85E-15 | BTG anti-proliferation factor 2 [Source:HGNC Symbol;Acc:HGNC:1131] | chr1 | + |
| [283] | KLF5 | ENSG00000102554.14 | 2.419831 | 3.69E-151 | Kruppel like factor 5 [Source:HGNC Symbol;Acc:HGNC:6349] | chr13 | + |
| [284] | SPTBN2 | ENSG00000173898.13 | 2.419723 | 0.000171 | spectrin beta, non-erythrocytic 2 [Source:HGNC Symbol;Acc:HGNC:11276] | chr11 | - |
| [285] | BIRC3 | ENSG00000023445.14 | 2.417533 | 6.33E-44 | baculoviral IAP repeat containing 3 [Source:HGNC Symbol;Acc:HGNC:591] | chr11 | + |
| [286] | AC139100.1 | ENSG00000267251.2 | 2.411606 | 0.000988 | novel transcript, antisense to PARD6G | chr18 | + |
| [287] | SOX2-OT | ENSG00000242808.9 | 2.410605 | 0.000546 | SOX2 overlapping transcript [Source:HGNC Symbol;Acc:HGNC:20209] | chr3 | + |
| [288] | VAMP8 | ENSG00000118640.11 | 2.408514 | 0.001283 | vesicle associated membrane protein 8 [Source:HGNC Symbol;Acc:HGNC:12647] | chr2 | + |
| [289] | AL031432.1 | ENSG00000231953.2 | 2.407127 | 0.001293 | novel transcript | chr1 | + |
| [290] | MARCKSL1 | ENSG00000175130.7 | 2.406513 | 1.14E-12 | MARCKS like 1 [Source:HGNC Symbol;Acc:HGNC:7142] | chr1 | - |
| [291] | FAM21FP | ENSG00000237840.6 | 2.397952 | 1.49E-06 | family with sequence similarity 21 member F, pseudogene [Source:HGNC Symbol;Acc:HGNC:45011] | chr10 | - |
| [292] | AC104530.1 | ENSG00000288156.1 | 2.397795 | 7.55E-05 | novel transcript | chr19 | + |
| [293] | DAPP1 | ENSG00000070190.13 | 2.395303 | 0.00014 | dual adaptor of phosphotyrosine and 3-phosphoinositides 1 | chr4 | + |

| | | | | | | | |
|---|---|---|---|---|---|---|---|
| | | | | | [Source:HGNC Symbol;Acc:HGNC:16500] | | |
| [294] | AL031432.5 | ENSG00000284657.1 | 2.385237 | 9.81E-05 | novel transcript | chr1 | + |
| [295] | OVOL1-AS1 | ENSG00000255120.5 | 2.38372 | 0.001458 | OVOL1 antisense RNA 1 [Source:HGNC Symbol;Acc:HGNC:49319] | chr11 | - |
| [296] | AC010336.8 | ENSG00000286138.1 | 2.382605 | 0.000507 | novel transcript | chr19 | + |
| [297] | PYDC1 | ENSG00000169900.7 | 2.381957 | 0.001472 | pyrin domain containing 1 [Source:HGNC Symbol;Acc:HGNC:30261] | chr16 | - |
| [298] | HOXD1 | ENSG00000128645.15 | 2.381009 | 0.000843 | homeobox D1 [Source:HGNC Symbol;Acc:HGNC:5132] | chr2 | + |
| [299] | LINC002481 | ENSG00000246526.2 | 2.375539 | 0.000706 | long intergenic non-protein coding RNA 2481 [Source:HGNC Symbol;Acc:HGNC:27958] | chr4 | - |
| [300] | HERPUD1 | ENSG00000051108.15 | 2.373438 | 6.31E-291 | homocysteine inducible ER protein with ubiquitin like domain 1 [Source:HGNC Symbol;Acc:HGNC:13744] | chr16 | + |
| [301] | AL133457.1 | ENSG00000261038.1 | 2.371722 | 0.001558 | novel transcript | chr6 | - |
| [302] | MT-TK | ENSG00000210156.1 | 2.36067 | 0.001325 | mitochondrially encoded tRNA-Lys (AAA/G) [Source:HGNC Symbol;Acc:HGNC:7489] | chrM | + |
| [303] | FLG | ENSG00000143631.10 | 2.357529 | 8.63E-37 | filaggrin [Source:HGNC Symbol;Acc:HGNC:3748] | chr1 | - |
| [304] | DRD5P1 | ENSG00000184303.5 | 2.356934 | 0.000522 | dopamine receptor D5 pseudogene 1 [Source:HGNC Symbol;Acc:HGNC:3027] | chr2 | - |
| [305] | DRAXIN | ENSG00000162490.7 | 2.350635 | 4.13E-05 | dorsal inhibitory axon guidance protein [Source:HGNC Symbol;Acc:HGNC:25054] | chr1 | + |
| [306] | CD300LB | ENSG00000178789.9 | 2.350051 | 0.000988 | CD300 molecule like family member b [Source:HGNC Symbol;Acc:HGNC:30811] | chr17 | - |
| [307] | AC087632.2 | ENSG00000259316.11 | 2.348215 | 0.000534 | novel protein | chr15 | - |
| [308] | GAPDHP33 | ENSG00000240244.3 | 2.340461 | 2.15E-05 | glyceraldehyde 3 phosphate dehydrogenase pseudogene 33 [Source:HGNC Symbol;Acc:HGNC:37785] | chr1 | - |
| [309] | PRX | ENSG00000105227.14 | 2.339117 | 1.25E-19 | periaxin [Source:HGNC Symbol;Acc:HGNC:13797] | chr19 | - |
| [310] | ABHD4 | ENSG00000100439.10 | 2.33717 | 3.57E-68 | abhydrolase domain containing 4, N-acyl phospholipase B [Source:HGNC Symbol;Acc:HGNC:20154] | chr14 | + |
| [311] | B3GNT7 | ENSG00000156966.7 | 2.330312 | 0.001152 | UDP-GlcNAc:betaGal beta-1,3-N-acetylglucosaminyltransferase 7 [Source:HGNC Symbol;Acc:HGNC:18811] | chr2 | + |
| [312] | AF127577.4 | ENSG00000235609.7 | 2.325928 | 4.89E-55 | novel transcript | chr21 | - |
| [313] | HSPA2 | ENSG00000126803.9 | 2.321252 | 1.30E-83 | heat shock protein family A (Hsp70) member 2 | chr14 | + |

| | | | | | | | |
|---|---|---|---|---|---|---|---|
| | | | | | [Source:HGNC Symbol;Acc:HGNC:5235] | | |
| [314] | TNFSF4 | ENSG00000117586.11 | 2.319433 | 0.000157 | TNF superfamily member 4 [Source:HGNC Symbol;Acc:HGNC:11934] | chr1 | - |
| [315] | HSPB9 | ENSG00000260325.2 | 2.319136 | 0.001666 | heat shock protein family B (small) member 9 [Source:HGNC Symbol;Acc:HGNC:30589] | chr17 | + |
| [316] | AOC2 | ENSG00000131480.9 | 2.318926 | 9.42E-14 | amine oxidase copper containing 2 [Source:HGNC Symbol;Acc:HGNC:549] | chr17 | + |
| [317] | ASNS | ENSG00000070669.17 | 2.318301 | 0 | asparagine synthetase (glutamine-hydrolyzing) [Source:HGNC Symbol;Acc:HGNC:753] | chr7 | - |
| [318] | RXFP4 | ENSG00000173080.5 | 2.316471 | 5.26E-08 | relaxin family peptide/INSL5 receptor 4 [Source:HGNC Symbol;Acc:HGNC:14666] | chr1 | + |
| [319] | HSD3BP5 | ENSG00000198857.3 | 2.314295 | 8.86E-21 | hydroxy-delta-5-steroid dehydrogenase, 3 beta, pseudogene 5 [Source:HGNC Symbol;Acc:HGNC:5223] | chr1 | + |
| [320] | CYP1A1 | ENSG00000140465.14 | 2.310071 | 4.83E-10 | cytochrome P450 family 1 subfamily A member 1 [Source:HGNC Symbol;Acc:HGNC:2595] | chr15 | - |
| [321] | AC037441.1 | ENSG00000248738.6 | 2.309595 | 0.002147 | novel transcript | chr8 | + |
| [322] | ST6GALNAC1 | ENSG00000070526.15 | 2.308784 | 0.002141 | ST6 N-acetylgalactosaminide alpha-2,6-sialyltransferase 1 [Source:HGNC Symbol;Acc:HGNC:23614] | chr17 | - |
| [323] | AC008969.1 | ENSG00000176593.8 | 2.308277 | 0.002161 | novel transcript, antisense to ZNF606 | chr19 | + |
| [324] | VWF | ENSG00000110799.13 | 2.303523 | 0.000227 | von Willebrand factor [Source:HGNC Symbol;Acc:HGNC:12726] | chr12 | - |
| [325] | ABCA11P | ENSG00000251595.7 | 2.302797 | 5.97E-05 | ATP binding cassette subfamily A member 11, pseudogene [Source:HGNC Symbol;Acc:HGNC:31] | chr4 | - |
| [326] | AC008063.2 | ENSG00000233397.3 | 2.297628 | 0.000627 | novel transcript | chr2 | + |
| [327] | MKNK2 | ENSG00000099875.14 | 2.288795 | 0 | MAPK interacting serine/threonine kinase 2 [Source:HGNC Symbol;Acc:HGNC:7111] | chr19 | - |
| [328] | CCND2 | ENSG00000118971.8 | 2.286916 | 0.00241 | cyclin D2 [Source:HGNC Symbol;Acc:HGNC:1583] | chr12 | + |
| [329] | C19orf38 | ENSG00000214212.9 | 2.286861 | 0.000156 | chromosome 19 open reading frame 38 [Source:HGNC Symbol;Acc:HGNC:34073] | chr19 | + |
| [330] | JMY | ENSG00000152409.9 | 2.285 | 3.61E-152 | junction mediating and regulatory protein, p53 cofactor [Source:HGNC Symbol;Acc:HGNC:28916] | chr5 | + |
| [331] | OR52K1 | ENSG00000196778.3 | 2.276702 | 0.001455 | olfactory receptor family 52 subfamily K member 1 | chr11 | + |

| | | | | | | | |
|---|---|---|---|---|---|---|---|
| | | | | | [Source:HGNC Symbol;Acc:HGNC:15222] | | |
| [332] | PMAIP1 | ENSG00000141682.11 | 2.274662 | 4.07E-196 | phorbol-12-myristate-13-acetate-induced protein 1 [Source:HGNC Symbol;Acc:HGNC:9108] | chr18 | + |
| [333] | LINC02009 | ENSG00000283646.2 | 2.273237 | 1.12E-05 | long intergenic non-protein coding RNA 2009 [Source:HGNC Symbol;Acc:HGNC:52845] | chr3 | - |
| [334] | SRSF12 | ENSG00000154548.9 | 2.267682 | 0.000317 | serine and arginine rich splicing factor 12 [Source:HGNC Symbol;Acc:HGNC:21220] | chr6 | - |
| [335] | HMX3 | ENSG00000188620.11 | 2.267039 | 0.000303 | H6 family homeobox 3 [Source:HGNC Symbol;Acc:HGNC:5019] | chr10 | + |
| [336] | CCDC62 | ENSG00000130783.14 | 2.265129 | 9.69E-05 | coiled-coil domain containing 62 [Source:HGNC Symbol;Acc:HGNC:30723] | chr12 | + |
| [337] | RIMKLA | ENSG00000177181.15 | 2.265126 | 0.000844 | ribosomal modification protein rimK like family member A [Source:HGNC Symbol;Acc:HGNC:28725] | chr1 | + |
| [338] | AL590822.3 | ENSG00000287356.1 | 2.261081 | 0.000723 | novel transcript, antisense to MORN1 | chr1 | + |
| [339] | LINC01081 | ENSG00000268754.4 | 2.261055 | 0.000458 | long intergenic non-protein coding RNA 1081 [Source:HGNC Symbol;Acc:HGNC:49124] | chr16 | - |
| [340] | MAP1LC3B | ENSG00000140941.14 | 2.260975 | 0 | microtubule associated protein 1 light chain 3 beta [Source:HGNC Symbol;Acc:HGNC:13352] | chr16 | + |
| [341] | OVOL1 | ENSG00000172818.10 | 2.257694 | 0.002784 | ovo like transcriptional repressor 1 [Source:HGNC Symbol;Acc:HGNC:8525] | chr11 | + |
| [342] | MST1L | ENSG00000186715.11 | 2.256896 | 0.002754 | macrophage stimulating 1 like [Source:HGNC Symbol;Acc:HGNC:7390] | chr1 | - |
| [343] | EGFL7 | ENSG00000172889.16 | 2.254288 | 0.001195 | EGF like domain multiple 7 [Source:HGNC Symbol;Acc:HGNC:20594] | chr9 | + |
| [344] | CCN2 | ENSG00000118523.6 | 2.250409 | 6.81E-155 | cellular communication network factor 2 [Source:HGNC Symbol;Acc:HGNC:2500] | chr6 | - |
| [345] | AC008969.2 | ENSG00000286949.1 | 2.246843 | 0.002892 | novel transcript | chr19 | - |
| [346] | GABRR2 | ENSG00000111886.11 | 2.246122 | 0.002001 | gamma-aminobutyric acid type A receptor subunit rho2 [Source:HGNC Symbol;Acc:HGNC:4091] | chr6 | - |
| [347] | AL121749.1 | ENSG00000273312.2 | 2.241643 | 0.003008 | novel transcript | chr10 | - |
| [348] | ATF4P3 | ENSG00000228218.1 | 2.239949 | 6.57E-112 | activating transcription factor 4 pseudogene 3 [Source:HGNC Symbol;Acc:HGNC:788] | chr17 | - |

| | | | | | | | |
|---|---|---|---|---|---|---|---|
| [349] | AL391427.1 | ENSG00000224251.6 | 2.234355 | 0.003073 | novel transcript, antisense to AKR1C2 | chr10 | + |
| [350] | ATF4P4 | ENSG00000256167.1 | 2.233408 | 2.79E-26 | activating transcription factor 4 pseudogene 4 [Source:HGNC Symbol;Acc:HGNC:787] | chr11 | + |
| [351] | LINC01655 | ENSG00000227925.2 | 2.226896 | 0.002813 | long intergenic non-protein coding RNA 1655 [Source:HGNC Symbol;Acc:HGNC:52443] | chr1 | - |
| [352] | BX005195.1 | ENSG00000276204.1 | 2.226896 | 0.002813 | calcium-sensing receptor (CASR) pseudogene | chr9 | - |
| [353] | LINC01447 | ENSG00000236078.2 | 2.226337 | 0.00319 | long intergenic non-protein coding RNA 1447 [Source:HGNC Symbol;Acc:HGNC:50783] | chr7 | + |
| [354] | AC243960.3 | ENSG00000268355.1 | 2.225275 | 0.003213 | novel transcript | chr19 | + |
| [355] | MUC20 | ENSG00000176945.17 | 2.223595 | 0.003238 | mucin 20, cell surface associated [Source:HGNC Symbol;Acc:HGNC:23282] | chr3 | + |
| [356] | TMEM268 | ENSG00000157693.15 | 2.220695 | 7.53E-53 | transmembrane protein 268 [Source:HGNC Symbol;Acc:HGNC:24513] | chr9 | + |
| [357] | AC134878.1 | ENSG00000274231.1 | 2.218299 | 4.66E-05 | asparagine synthetase (glutamine-hydrolyzing) (ASNS) pseudogene | chrY | + |
| [358] | LINC02427 | ENSG00000271538.6 | 2.214038 | 0.002283 | long intergenic non-protein coding RNA 2427 [Source:HGNC Symbol;Acc:HGNC:53358] | chr4 | - |
| [359] | KCNT1 | ENSG00000107147.13 | 2.212898 | 0.003462 | potassium sodium-activated channel subfamily T member 1 [Source:HGNC Symbol;Acc:HGNC:18865] | chr9 | + |
| [360] | IL19 | ENSG00000142224.16 | 2.21272 | 0.003464 | interleukin 19 [Source:HGNC Symbol;Acc:HGNC:5990] | chr1 | + |
| [361] | RPS7P2 | ENSG00000231837.2 | 2.21272 | 0.003464 | ribosomal protein S7 pseudogene 2 [Source:HGNC Symbol;Acc:HGNC:36079] | chr1 | + |
| [362] | HSPA1A | ENSG00000204389.10 | 2.209338 | 8.81E-64 | heat shock protein family A (Hsp70) member 1A [Source:HGNC Symbol;Acc:HGNC:5232] | chr6 | + |
| [363] | ANKRD22 | ENSG00000152766.6 | 2.208239 | 0.000342 | ankyrin repeat domain 22 [Source:HGNC Symbol;Acc:HGNC:28321] | chr10 | - |
| [364] | NXF1 | ENSG00000162231.14 | 2.207411 | 0 | nuclear RNA export factor 1 [Source:HGNC Symbol;Acc:HGNC:8071] | chr11 | - |
| [365] | FIBIN | ENSG00000176971.4 | 2.203896 | 2.00E-21 | fin bud initiation factor homoLog [Source:HGNC Symbol;Acc:HGNC:33747] | chr11 | + |
| [366] | AL022476.1 | ENSG00000230319.1 | 2.203567 | 0.002489 | TTLL1 antisense RNA 1 [Source:HGNC Symbol;Acc:HGNC:40111] | chr22 | + |
| [367] | DGCR9 | ENSG00000273032.2 | 2.20235 | 8.44E-05 | DiGeorge syndrome critical region gene 5 [Source:NCBI | chr22 | + |

| | | | | | | | |
|---|---|---|---|---|---|---|---|
| | | | | | gene (formerly Entrezgene);Acc:26220] | | |
| [368] | SLC6A12 | ENSG00000111181.12 | 2.200025 | 0.003609 | solute carrier family 6 member 12 [Source:HGNC Symbol;Acc:HGNC:11045] | chr12 | - |
| [369] | NGFR | ENSG00000064300.9 | 2.198736 | 0.003293 | nerve growth factor receptor [Source:HGNC Symbol;Acc:HGNC:7809] | chr17 | + |
| [370] | RNU2-62P | ENSG00000222800.1 | 2.198641 | 0.003294 | RNA, U2 small nuclear 62, pseudogene [Source:HGNC Symbol;Acc:HGNC:48555] | chr6 | - |
| [371] | MUC16 | ENSG00000181143.15 | 2.192521 | 0.003737 | mucin 16, cell surface associated [Source:HGNC Symbol;Acc:HGNC:15582] | chr19 | - |
| [372] | ADCY10P1 | ENSG00000161912.18 | 2.189565 | 0.00372 | ADCY10 pseudogene 1 [Source:HGNC Symbol;Acc:HGNC:44143] | chr6 | + |
| [373] | NGF | ENSG00000134259.3 | 2.184012 | 5.24E-12 | nerve growth factor [Source:HGNC Symbol;Acc:HGNC:7808] | chr1 | - |
| [374] | AL138878.2 | ENSG00000234115.2 | 2.182445 | 2.33E-20 | isocitrate dehydrogenase 1 (NADP+), soluble (IDH1) pseudogene | chr6 | - |
| [375] | CD248 | ENSG00000174807.4 | 2.181689 | 0.003621 | CD248 molecule [Source:HGNC Symbol;Acc:HGNC:18219] | chr11 | - |
| [376] | AC017104.2 | ENSG00000230047.1 | 2.18114 | 0.003636 | HIGD2A pseudogene 1 [Source:HGNC Symbol;Acc:HGNC:54793] | chr2 | - |
| [377] | COL23A1 | ENSG00000050767.17 | 2.177351 | 0.004022 | collagen type XXIII alpha 1 chain [Source:HGNC Symbol;Acc:HGNC:22990] | chr5 | - |
| [378] | AC017104.1 | ENSG00000224376.1 | 2.170292 | 0.002351 | novel transcript | chr2 | + |
| [379] | ZNF554 | ENSG00000172006.12 | 2.168949 | 1.13E-15 | zinc finger protein 554 [Source:HGNC Symbol;Acc:HGNC:26629] | chr19 | + |
| [380] | RAB44 | ENSG00000255587.9 | 2.168892 | 0.004177 | RAB44, member RAS oncogene family [Source:HGNC Symbol;Acc:HGNC:21068] | chr6 | + |
| [381] | MTTP | ENSG00000138823.13 | 2.161824 | 0.004378 | microsomal triglyceride transfer protein [Source:HGNC Symbol;Acc:HGNC:7467] | chr4 | + |
| [382] | MRGPRE | ENSG00000184350.11 | 2.160801 | 0.003293 | MAS related GPR family member E [Source:HGNC Symbol;Acc:HGNC:30694] | chr11 | - |
| [383] | STX17-AS1 | ENSG00000255145.3 | 2.159894 | 0.004339 | STX17 antisense RNA 1 [Source:HGNC Symbol;Acc:HGNC:51174] | chr9 | - |
| [384] | PART1 | ENSG00000152931.8 | 2.155555 | 1.23E-07 | prostate androgen-regulated transcript 1 [Source:HGNC Symbol;Acc:HGNC:17263] | chr5 | + |
| [385] | CMYA5 | ENSG00000164309.15 | 2.154561 | 6.24E-12 | cardiomyopathy associated 5 [Source:HGNC Symbol;Acc:HGNC:14305] | chr5 | + |

| | | | | | | | |
|---|---|---|---|---|---|---|---|
| [386] | TAS1R1 | ENSG00000173662.21 | 2.154344 | 0.00446 | taste 1 receptor member 1 [Source:HGNC Symbol;Acc:HGNC:14448] | chr1 | + |
| [387] | AC068722.2 | ENSG00000273972.1 | 2.147824 | 0.00469 | novel transcript | chr15 | + |
| [388] | PHF24 | ENSG00000122733.12 | 2.143271 | 0.001524 | PHD finger protein 24 [Source:HGNC Symbol;Acc:HGNC:29180] | chr9 | + |
| [389] | RPTN | ENSG00000215853.3 | 2.141245 | 1.62E-07 | repetin [Source:HGNC Symbol;Acc:HGNC:26809] | chr1 | - |
| [390] | MAF | ENSG00000178573.7 | 2.140444 | 1.14E-07 | MAF bZIP transcription factor [Source:HGNC Symbol;Acc:HGNC:6776] | chr16 | - |
| [391] | FBXO32 | ENSG00000156804.7 | 2.139916 | 0 | F-box protein 32 [Source:HGNC Symbol;Acc:HGNC:16731] | chr8 | - |
| [392] | KCNJ2 | ENSG00000123700.4 | 2.138461 | 1.30E-30 | potassium inwardly rectifying channel subfamily J member 2 [Source:HGNC Symbol;Acc:HGNC:6263] | chr17 | + |
| [393] | CLEC18B | ENSG00000140839.11 | 2.134213 | 0.003555 | C-type lectin domain family 18 member B [Source:HGNC Symbol;Acc:HGNC:33849] | chr16 | - |
| [394] | CACNA1I | ENSG00000100346.18 | 2.133684 | 0.004897 | calcium voltage-gated channel subunit alpha1 I [Source:HGNC Symbol;Acc:HGNC:1396] | chr22 | + |
| [395] | TNNI3 | ENSG00000129991.13 | 2.132414 | 0.004942 | troponin I3, cardiac type [Source:HGNC Symbol;Acc:HGNC:11947] | chr19 | - |
| [396] | AL158151.4 | ENSG00000268707.1 | 2.130865 | 0.003714 | novel transcript | chr9 | + |
| [397] | RFPL3S | ENSG00000205853.11 | 2.128962 | 3.58E-05 | RFPL3 antisense [Source:HGNC Symbol;Acc:HGNC:9981] | chr22 | - |
| [398] | KRT18P61 | ENSG00000267083.1 | 2.127079 | 0.002928 | keratin 18 pseudogene 61 [Source:HGNC Symbol;Acc:HGNC:48888] | chr17 | + |
| [399] | SESN3 | ENSG00000149212.12 | 2.124326 | 6.04E-60 | sestrin 3 [Source:HGNC Symbol;Acc:HGNC:23060] | chr11 | - |
| [400] | Z83844.3 | ENSG00000285304.1 | 2.121544 | 0.005332 | novel protein | chr22 | + |
| [401] | CSTA | ENSG00000121552.4 | 2.120137 | 1.87E-07 | cystatin A [Source:HGNC Symbol;Acc:HGNC:2481] | chr3 | + |
| [402] | BGLAP | ENSG00000242252.2 | 2.119422 | 0.002061 | bone gamma-carboxyglutamate protein [Source:HGNC Symbol;Acc:HGNC:1043] | chr1 | + |
| [403] | PLK2 | ENSG00000145632.15 | 2.118039 | 0 | polo like kinase 2 [Source:HGNC Symbol;Acc:HGNC:19699] | chr5 | - |
| [404] | CDC42BPG | ENSG00000171219.9 | 2.117367 | 0.005264 | CDC42 binding protein kinase gamma [Source:HGNC Symbol;Acc:HGNC:29829] | chr11 | - |
| [405] | AC012181.1 | ENSG00000261114.1 | 2.117039 | 4.98E-05 | novel transcript, sense intronic to HERPUD1 | chr16 | + |
| [406] | RAD51AP2 | ENSG00000214842.5 | 2.11423 | 0.005489 | RAD51 associated protein 2 [Source:HGNC Symbol;Acc:HGNC:34417] | chr2 | - |
| [407] | AC010198.1 | ENSG00000246331.2 | 2.114213 | 0.005489 | novel transcript | chr12 | + |

| | | | | | | | |
|---|---|---|---|---|---|---|---|
| [408] | AC107959.1 | ENSG00000245025.3 | 2.114187 | 8.80E-49 | novel transcript, antisense to RHOBTB2 | chr8 | - |
| [409] | CETP | ENSG00000087237.12 | 2.111445 | 0.002087 | cholesteryl ester transfer protein [Source:HGNC Symbol;Acc:HGNC:1869] | chr16 | + |
| [410] | NCOA7 | ENSG00000111912.20 | 2.105356 | 1.50E-171 | nuclear receptor coactivator 7 [Source:HGNC Symbol;Acc:HGNC:21081] | chr6 | + |
| [411] | STK32A | ENSG00000169302.16 | 2.103708 | 1.72E-21 | serine/threonine kinase 32A [Source:HGNC Symbol;Acc:HGNC:28317] | chr5 | + |
| [412] | ANPEP | ENSG00000166825.14 | 2.101163 | 0.005663 | alanyl aminopeptidase, membrane [Source:HGNC Symbol;Acc:HGNC:500] | chr15 | - |
| [413] | AL035448.1 | ENSG00000236526.1 | 2.100822 | 0.005669 | novel transcript | chr20 | - |
| [414] | SPX | ENSG00000134548.11 | 2.099621 | 0.000178 | spexin hormone [Source:HGNC Symbol;Acc:HGNC:28139] | chr12 | + |
| [415] | AL031432.4 | ENSG00000284602.1 | 2.096212 | 8.06E-09 | novel transcript | chr1 | + |
| [416] | TTN-AS1 | ENSG00000237298.10 | 2.095817 | 0.002821 | TTN antisense RNA 1 [Source:HGNC Symbol;Acc:HGNC:44124] | chr2 | + |
| [417] | MISP | ENSG00000099812.9 | 2.090049 | 0.004442 | mitotic spindle positioning [Source:HGNC Symbol;Acc:HGNC:27000] | chr19 | + |
| [418] | IL1A | ENSG00000115008.5 | 2.088719 | 1.73E-19 | interleukin 1 alpha [Source:HGNC Symbol;Acc:HGNC:5991] | chr2 | - |
| [419] | LINC02019 | ENSG00000273356.1 | 2.08248 | 6.97E-05 | long intergenic non-protein coding RNA 2019 [Source:HGNC Symbol;Acc:HGNC:52854] | chr3 | + |
| [420] | AC002331.1 | ENSG00000286712.1 | 2.081342 | 0.006339 | novel transcript | chr16 | + |
| [421] | AC135506.1 | ENSG00000270441.1 | 2.080522 | 0.006215 | laminin subunit beta 2 pseudogene 1 [Source:HGNC Symbol;Acc:HGNC:6488] | chr3 | - |
| [422] | NR4A3 | ENSG00000119508.18 | 2.07209 | 4.54E-15 | nuclear receptor subfamily 4 group A member 3 [Source:HGNC Symbol;Acc:HGNC:7982] | chr9 | + |
| [423] | AC004988.1 | ENSG00000203446.2 | 2.071683 | 1.88E-14 | SUGCT antisense RNA 1 [Source:HGNC Symbol;Acc:HGNC:40745] | chr7 | - |
| [424] | HEXIM1 | ENSG00000186834.3 | 2.070688 | 4.96E-132 | HEXIM P-TEFb complex subunit 1 [Source:HGNC Symbol;Acc:HGNC:24953] | chr17 | + |
| [425] | LRATD2 | ENSG00000168672.4 | 2.070662 | 2.86E-10 | LRAT domain containing 2 [Source:HGNC Symbol;Acc:HGNC:24166] | chr8 | - |
| [426] | ITGB4 | ENSG00000132470.14 | 2.066428 | 0.006042 | integrin subunit beta 4 [Source:HGNC Symbol;Acc:HGNC:6158] | chr17 | + |
| [427] | AC005593.1 | ENSG00000279584.1 | 2.065941 | 0.006575 | TEC | chr5 | + |
| [428] | AC127002.2 | ENSG00000275265.1 | 2.06204 | 0.003807 | novel transcript | chr12 | + |
| [429] | TSPOAP1 | ENSG00000005379.17 | 2.061452 | 0.00692 | TSPO associated protein 1 [Source:HGNC Symbol;Acc:HGNC:16831] | chr17 | - |

| | | | | | | | |
|---|---|---|---|---|---|---|---|
| [430] | CH25H | ENSG00000138135.7 | 2.060389 | 1.37E-05 | cholesterol 25-hydroxylase [Source:HGNC Symbol;Acc:HGNC:1907] | chr10 | - |
| [431] | AC090004.2 | ENSG00000287059.1 | 2.05787 | 0.000341 | novel transcript | chr3 | - |
| [432] | ANKRD18CP | ENSG00000159712.10 | 2.056538 | 0.004932 | ankyrin repeat domain 18C, pseudogene [Source:HGNC Symbol;Acc:HGNC:43601] | chr9 | - |
| [433] | AC073111.3 | ENSG00000284048.2 | 2.056508 | 0.005906 | novel transcript | chr7 | + |
| [434] | CD55 | ENSG00000196352.15 | 2.054501 | 7.88E-218 | CD55 molecule (Cromer blood group) [Source:HGNC Symbol;Acc:HGNC:2665] | chr1 | + |
| [435] | AC108517.1 | ENSG00000249111.1 | 2.05067 | 2.14E-07 | novel transcript | chr4 | + |
| [436] | C16orf46 | ENSG00000166455.13 | 2.047763 | 0.002585 | chromosome 16 open reading frame 46 [Source:HGNC Symbol;Acc:HGNC:26525] | chr16 | - |
| [437] | AC073283.3 | ENSG00000273269.3 | 2.047181 | 0.007148 | novel transcript | chr2 | - |
| [438] | SLIT1 | ENSG00000187122.17 | 2.041772 | 0.005318 | slit guidance ligand 1 [Source:HGNC Symbol;Acc:HGNC:11085] | chr10 | - |
| [439] | PLA2G4D | ENSG00000159337.7 | 2.04174 | 0.007303 | phospholipase A2 group IVD [Source:HGNC Symbol;Acc:HGNC:30038] | chr15 | - |
| [440] | AC024082.1 | ENSG00000231923.1 | 2.03749 | 0.006229 | Der1-like domain family, member 2 (DERL2) pseudogene | chr7 | + |
| [441] | PTPRR | ENSG00000153233.13 | 2.034595 | 5.25E-24 | protein tyrosine phosphatase receptor type R [Source:HGNC Symbol;Acc:HGNC:9680] | chr12 | - |
| [442] | NTN4 | ENSG00000074527.12 | 2.03456 | 6.22E-20 | netrin 4 [Source:HGNC Symbol;Acc:HGNC:13658] | chr12 | - |
| [443] | FAM209A | ENSG00000124103.9 | 2.033612 | 0.005665 | family with sequence similarity 209 member A [Source:HGNC Symbol;Acc:HGNC:16100] | chr20 | + |
| [444] | PCDHA12 | ENSG00000251664.5 | 2.031779 | 0.005845 | protocadherin alpha 12 [Source:HGNC Symbol;Acc:HGNC:8666] | chr5 | + |
| [445] | GADD45A | ENSG00000116717.13 | 2.03003 | 6.32E-197 | growth arrest and DNA damage inducible alpha [Source:HGNC Symbol;Acc:HGNC:4095] | chr1 | + |
| [446] | TMEM269 | ENSG00000274386.5 | 2.029629 | 0.003332 | transmembrane protein 269 [Source:HGNC Symbol;Acc:HGNC:52381] | chr1 | + |
| [447] | AC003005.1 | ENSG00000268107.6 | 2.028118 | 0.001761 | novel transcript | chr19 | + |
| [448] | LINC00862 | ENSG00000203721.7 | 2.025027 | 0.00159 | long intergenic non-protein coding RNA 862 [Source:HGNC Symbol;Acc:HGNC:21901] | chr1 | - |
| [449] | HEATR4 | ENSG00000187105.8 | 2.024636 | 0.005718 | HEAT repeat containing 4 [Source:HGNC Symbol;Acc:HGNC:16761] | chr14 | - |
| [450] | AC016925.3 | ENSG00000287796.1 | 2.024422 | 0.008198 | novel transcript | chrX | + |
| [451] | AC011365.1 | ENSG00000254042.2 | 2.018378 | 0.00578 | SLIT3 antisense RNA 2 [Source:HGNC Symbol;Acc:HGNC:40551] | chr5 | + |

| | | | | | | | |
|---|---|---|---|---|---|---|---|
| [452] | NEBL | ENSG00000078114.18 | 2.01785 | 0.001878 | nebulette [Source:HGNC Symbol;Acc:HGNC:16932] | chr10 | - |
| [453] | CTNNA3 | ENSG00000183230.17 | 2.012064 | 0.008428 | catenin alpha 3 [Source:HGNC Symbol;Acc:HGNC:2511] | chr10 | - |
| [454] | PINCR | ENSG00000224294.1 | 2.009538 | 0.008782 | p53-induced noncoding RNA [Source:HGNC Symbol;Acc:HGNC:53590] | chrX | + |
| [455] | MLC1 | ENSG00000100427.15 | 2.009266 | 0.00844 | modulator of VRAC current 1 [Source:HGNC Symbol;Acc:HGNC:17082] | chr22 | - |
| [456] | RNU6-781P | ENSG00000252186.1 | 2.009142 | 0.008528 | RNA, U6 small nuclear 781, pseudogene [Source:HGNC Symbol;Acc:HGNC:47744] | chr12 | + |
| [457] | SNX31 | ENSG00000174226.9 | 2.008973 | 0.008409 | sorting nexin 31 [Source:HGNC Symbol;Acc:HGNC:28605] | chr8 | - |
| [458] | RNF223 | ENSG00000237330.3 | 2.007028 | 0.008473 | ring finger protein 223 [Source:HGNC Symbol;Acc:HGNC:40020] | chr1 | - |
| [459] | AC138356.3 | ENSG00000253181.2 | 2.005857 | 0.008549 | novel transcript | chr8 | + |
| [460] | BTG1 | ENSG00000133639.5 | 2.00555 | 1.47E-147 | BTG anti-proliferation factor 1 [Source:HGNC Symbol;Acc:HGNC:1130] | chr12 | - |
| [461] | LINC01822 | ENSG00000229621.2 | 2.000452 | 0.004249 | long intergenic non-protein coding RNA 1822 [Source:HGNC Symbol;Acc:HGNC:52627] | chr2 | - |
