# Supplementary material for: In Vitro and In Vivo Enhancement of Temozolomide Effect in Human Glioblastoma by Non-Invasive Application of Cold Atmospheric Plasma
Source: Cancers (Basel). 2021 Sep 6;13(17):4485. doi: 10.3390/cancers13174485 (PMC8430547; doi:10.3390/cancers13174485)
Supplement: Supplementary file 1 [file cancers-13-04485-s001.zip › SI Table S6 - Top downregulated genes in CAP + TMZ treatment.pdf]

Supplementary Table 6: List of top 448 down-regulated genes in the case of CAP + TMZ treatment

Sr. No	Gene Name	GeneID	Log2(FC)	P-adj	Description	Chromosome	Strand
[1]	AC004949.1	ENSG00000283117.1	-7.64067	2.05E-52	uncharacterized LOC79150 [Source:NCBI gene (formerly Entrezgene);Acc:79150]	chr7	-
[2]	PDXP	ENSG00000241360.2	-6.28187	4.27E-31	pyridoxal phosphatase [Source:HGNC Symbol;Acc:HGNC:30259]	chr22	+
[3]	AC009133.6	ENSG00000280893.1	-6.16662	1.27E-29	novel transcript	chr16	+
[4]	SVIL2P	ENSG00000234814.8	-5.14657	1.12E-18	supervillin family member 2, pseudogene [Source:HGNC Symbol;Acc:HGNC:44959]	chr10	+
[5]	FAM72D	ENSG00000215784.6	-4.89078	2.03E-16	family with sequence similarity 72 member D [Source:HGNC Symbol;Acc:HGNC:33593]	chr1	+
[6]	RN7SKP230	ENSG00000202512.1	-4.78546	9.89E-189	RN7SK pseudogene 230 [Source:HGNC Symbol;Acc:HGNC:45954]	chr5	-
[7]	SNORA48	ENSG00000209582.1	-4.75196	2.58E-39	small nucleolar RNA, H/ACA box 48 [Source:HGNC Symbol;Acc:HGNC:32641]	chr17	+
[8]	Y_RNA	ENSG00000200494.1	-4.73713	4.99E-21	Y RNA [Source:RFAM;Acc:RF00019]	chr20	-
[9]	RNY3P1	ENSG00000201955.1	-4.73713	4.99E-21	RNY3 pseudogene 1 [Source:HGNC Symbol;Acc:HGNC:42477]	chr5	-
[10]	RN7SL674P	ENSG00000239899.3	-4.73331	0	RNA, 7SL, cytoplasmic 674, pseudogene [Source:HGNC Symbol;Acc:HGNC:46690]	chr2	+
[11]	SNORA57	ENSG00000206597.1	-4.64238	2.64E-40	small nucleolar RNA, H/ACA box 57 [Source:HGNC Symbol;Acc:HGNC:32651]	chr11	+
[12]	RNY3	ENSG00000202354.1	-4.56784	4.44E-21	RNA, Ro60-associated Y3 [Source:HGNC Symbol;Acc:HGNC:10243]	chr7	+
[13]	Y_RNA	ENSG00000207142.1	-4.56784	4.44E-21	Y RNA [Source:RFAM;Acc:RF00019]	chr12	+
[14]	RN7SL128P	ENSG00000240869.3	-4.54329	0	RNA, 7SL, cytoplasmic 128, pseudogene [Source:HGNC Symbol;Acc:HGNC:46144]	chr6	-
[15]	HIST1H2BM	ENSG00000273703.1	-4.54223	6.11E-98	H2B clustered histone 14 [Source:HGNC Symbol;Acc:HGNC:4750]	chr6	+

[16]	RNU4-2	ENSG00000202538.1	-4.3821	7.07E-48	RNA, U4 small nuclear 2 [Source:HGNC Symbol;Acc:HGNC:10193]	chr12	-
[17]	SMIM11B	ENSG00000273590.4	-4.28122	1.78E-11	small integral membrane protein 11B [Source:HGNC Symbol;Acc:HGNC:51846]	chr21	+
[18]	SNORA63	ENSG00000200320.1	-4.27003	1.99E-17	small nucleolar RNA, H/ACA box 63 [Source:HGNC Symbol;Acc:HGNC:10106]	chr3	+
[19]	MT-TF	ENSG00000210049.1	-4.26287	1.41E-17	mitochondrially encoded tRNA-Phe (UUU/C) [Source:HGNC Symbol;Acc:HGNC:7481]	chrM	+
[20]	AC051619.8	ENSG00000260035.1	-4.20922	1.17E-15	novel transcript	chr15	-
[21]	HIST1H2AB	ENSG00000278463.1	-4.19822	1.23E-77	H2A clustered histone 4 [Source:HGNC Symbol;Acc:HGNC:4734]	chr6	-
[22]	ATP23	ENSG00000166896.9	-4.17645	3.59E-11	ATP23 metallopeptidase and ATP synthase assembly factor homoLog [Source:HGNC Symbol;Acc:HGNC:29452]	chr12	+
[23]	CDC42EP2	ENSG00000149798.5	-4.15609	1.36E-10	CDC42 effector protein 2 [Source:HGNC Symbol;Acc:HGNC:16263]	chr11	+
[24]	RNU4-1	ENSG00000200795.1	-4.12504	9.63E-42	RNA, U4 small nuclear 1 [Source:HGNC Symbol;Acc:HGNC:10192]	chr12	-
[25]	RNU5F-1	ENSG00000199377.1	-4.07629	2.17E-14	RNA, U5F small nuclear 1 [Source:HGNC Symbol;Acc:HGNC:10216]	chr1	-
[26]	HIST2H3D	ENSG00000183598.3	-4.06289	7.80E-163	H3 clustered histone 13 [Source:HGNC Symbol;Acc:HGNC:25311]	chr1	-
[27]	NMU	ENSG00000109255.11	-4.06092	1.87E-10	neuromedin U [Source:HGNC Symbol;Acc:HGNC:7859]	chr4	-
[28]	HIST1H3J	ENSG00000197153.4	-4.03104	1.11E-140	H3 clustered histone 12 [Source:HGNC Symbol;Acc:HGNC:4774]	chr6	-
[29]	HIST1H2AI	ENSG00000196747.4	-3.94409	2.19E-183	H2A clustered histone 13 [Source:HGNC Symbol;Acc:HGNC:4725]	chr6	+
[30]	HIST1H3F	ENSG00000277775.1	-3.85179	3.41E-263	H3 clustered histone 7 [Source:HGNC Symbol;Acc:HGNC:4773]	chr6	-
[31]	ELMO3	ENSG00000102890.16	-3.81734	4.51E-09	engulfment and cell motility 3 [Source:HGNC Symbol;Acc:HGNC:17289]	chr16	+

[32]	HIST1H1B	ENSG00000184357.4	-3.78791	0	H1.5 linker histone, cluster member [Source:HGNC Symbol;Acc:HGNC:4719]	chr6	-
[33]	AC010624.5	ENSG00000287001.1	-3.78161	6.72E-09	novel transcript	chr19	-
[34]	Y_RNA	ENSG00000199677.1	-3.7704	6.16E-11	Y RNA [Source:RFAM;Acc:RF00019]	chr5	-
[35]	HIST1H3G	ENSG00000273983.1	-3.73645	1.04E-208	H3 clustered histone 8 [Source:HGNC Symbol;Acc:HGNC:4772]	chr6	-
[36]	SCARNA13	ENSG00000252481.1	-3.73281	4.84E-37	small Cajal body-specific RNA 13 [Source:HGNC Symbol;Acc:HGNC:32570]	chr14	-
[37]	CRYZL2P-SEC16B	ENSG00000254154.8	-3.72005	1.54E-08	CRYZL2P-SEC16B readthrough [Source:HGNC Symbol;Acc:HGNC:53757]	chr1	-
[38]	HIST1H2AL	ENSG00000276903.1	-3.71452	2.79E-161	H2A clustered histone 16 [Source:HGNC Symbol;Acc:HGNC:4730]	chr6	+
[39]	AC092641.1	ENSG00000227176.1	-3.69885	1.82E-08	NSA2 pseudogene 5 [Source:HGNC Symbol;Acc:HGNC:54584]	chr2	-
[40]	COL1A1	ENSG00000108821.13	-3.69756	0	collagen type I alpha 1 chain [Source:HGNC Symbol;Acc:HGNC:2197]	chr17	-
[41]	SNORA80A	ENSG00000200792.1	-3.69333	1.96E-08	small nucleolar RNA, H/ACA box 80A [Source:HGNC Symbol;Acc:HGNC:32666]	chr21	-
[42]	RN7SL767P	ENSG00000241529.3	-3.68781	4.29E-26	RNA, 7SL, cytoplasmic 767, pseudogene [Source:HGNC Symbol;Acc:HGNC:46783]	chr3	+
[43]	C4orf48	ENSG00000243449.6	-3.64586	3.45E-08	chromosome 4 open reading frame 48 [Source:HGNC Symbol;Acc:HGNC:34437]	chr4	+
[44]	MBOAT1	ENSG00000172197.11	-3.63223	4.29E-08	membrane bound O-acyltransferase domain containing 1 [Source:HGNC Symbol;Acc:HGNC:21579]	chr6	-
[45]	Y_RNA	ENSG00000200314.1	-3.6222	5.79E-10	Y RNA [Source:RFAM;Acc:RF00019]	chr6	+
[46]	HIST1H3B	ENSG00000286522.1	-3.59303	0	H3 clustered histone 2 [Source:HGNC Symbol;Acc:HGNC:4776]	chr6	-
[47]	HIST1H2AJ	ENSG00000276368.1	-3.55481	7.31E-164	H2A clustered histone 14 [Source:HGNC Symbol;Acc:HGNC:4727]	chr6	-

[48]	SNORD10	ENSG00000238917.1	-3.54538	3.99E-11	small nucleolar RNA, C/D box 10 [Source:HGNC Symbol;Acc:HGNC:32706]	chr17	+
[49]	GRIA3	ENSG00000125675.18	-3.54277	1.14E-07	glutamate ionotropic receptor AMPA type subunit 3 [Source:HGNC Symbol;Acc:HGNC:4573]	chrX	+
[50]	U1	ENSG00000270722.1	-3.52911	7.40E-13	RNA, variant U1 small nuclear 31 [Source:HGNC Symbol;Acc:HGNC:54448]	chr1	-
[51]	HIST1H2BI	ENSG00000278588.1	-3.52812	9.39E-149	H2B clustered histone 10 [Source:HGNC Symbol;Acc:HGNC:4756]	chr6	+
[52]	AL162151.2	ENSG00000234648.1	-3.52398	3.87E-13	pseudogene similar to part of ribosomal protein L3 (RPL3)	chr14	+
[53]	SNORA54	ENSG00000207008.1	-3.47616	7.42E-11	small nucleolar RNA, H/ACA box 54 [Source:HGNC Symbol;Acc:HGNC:32647]	chr11	-
[54]	MAGOH3P	ENSG00000270975.1	-3.46821	2.28E-07	mago homoLog 3, pseudogene [Source:HGNC Symbol;Acc:HGNC:20020]	chr14	+
[55]	HIST1H2BJ	ENSG00000124635.8	-3.44185	2.00E-158	H2B clustered histone 11 [Source:HGNC Symbol;Acc:HGNC:4761]	chr6	-
[56]	HIST1H3I	ENSG00000275379.1	-3.42477	1.75E-59	H3 clustered histone 11 [Source:HGNC Symbol;Acc:HGNC:4771]	chr6	-
[57]	HIST1H4A	ENSG00000278637.1	-3.42257	7.68E-51	H4 clustered histone 1 [Source:HGNC Symbol;Acc:HGNC:4781]	chr6	+
[58]	ARHGAP11B	ENSG00000284906.1	-3.40476	1.08E-06	novel transcript	chr15	+
[59]	HIST1H1A	ENSG00000124610.4	-3.39934	4.10E-36	H1.1 linker histone, cluster member [Source:HGNC Symbol;Acc:HGNC:4715]	chr6	-
[60]	ASPM	ENSG00000066279.18	-3.37375	0	assembly factor for spindle microtubules [Source:HGNC Symbol;Acc:HGNC:19048]	chr1	-
[61]	HIST1H4L	ENSG00000275126.1	-3.36571	4.51E-62	H4 clustered histone 13 [Source:HGNC Symbol;Acc:HGNC:4791]	chr6	-
[62]	HIST2H3DP1	ENSG00000213244.3	-3.34614	2.34E-43	H3 histone pseudogene 4 [Source:HGNC Symbol;Acc:HGNC:43797]	chr1	-
[63]	MT-TN	ENSG00000210135.1	-3.34333	9.86E-57	mitochondrially encoded tRNA-Asn (AAU/C) [Source:HGNC Symbol;Acc:HGNC:7493]	chrM	-
[64]	AC008894.3	ENSG00000279198.1	-3.34103	2.53E-35	TEC	chr19	-

[65]	ST18	ENSG00000147488.11	-3.33726	8.41E-14	ST18 C2H2C-type zinc finger transcription factor [Source:HGNC Symbol;Acc:HGNC:18695]	chr8	-
[66]	C1QTNF9B	ENSG00000205863.10	-3.32498	9.81E-07	C1q and TNF related 9B [Source:HGNC Symbol;Acc:HGNC:34072]	chr13	-
[67]	AP005233.2	ENSG00000260877.2	-3.30548	1.22E-06	novel transcript	chr11	+
[68]	COL5A3	ENSG00000080573.7	-3.29474	1.24E-08	collagen type V alpha 3 chain [Source:HGNC Symbol;Acc:HGNC:14864]	chr19	-
[69]	GNB3	ENSG00000111664.10	-3.29408	6.73E-08	G protein subunit beta 3 [Source:HGNC Symbol;Acc:HGNC:4400]	chr12	+
[70]	HIST1H2AH	ENSG00000274997.1	-3.29092	6.16E-151	H2A clustered histone 12 [Source:HGNC Symbol;Acc:HGNC:13671]	chr6	+
[71]	AC008163.1	ENSG00000233491.8	-3.27994	1.53E-06	novel pseudogene containing cadherin repeats	chr7	-
[72]	DNLZ	ENSG00000213221.5	-3.27207	1.98E-10	DNL-type zinc finger [Source:HGNC Symbol;Acc:HGNC:33879]	chr9	-
[73]	SNORA7B	ENSG00000207088.1	-3.25591	8.09E-08	small nucleolar RNA, H/ACA box 7B [Source:HGNC Symbol;Acc:HGNC:32593]	chr3	-
[74]	PHLDA2	ENSG00000181649.8	-3.25494	2.01E-06	pleckstrin homoLogy like domain family A member 2 [Source:HGNC Symbol;Acc:HGNC:12385]	chr11	-
[75]	SNORA71D	ENSG00000200354.1	-3.25082	6.29E-08	small nucleolar RNA, H/ACA box 71D [Source:HGNC Symbol;Acc:HGNC:32657]	chr20	-
[76]	HIST2H3C	ENSG00000203811.1	-3.23029	0	H3 clustered histone 14 [Source:HGNC Symbol;Acc:HGNC:20503]	chr1	-
[77]	HIST2H3A	ENSG00000203852.3	-3.22989	0	H3 clustered histone 15 [Source:HGNC Symbol;Acc:HGNC:20505]	chr1	+
[78]	AC079313.1	ENSG00000258086.1	-3.21579	3.18E-06	novel transcript, antisense to ZNF385A, GPR84, ITGA5 and GTSF1	chr12	+
[79]	RNU2-2P	ENSG00000222328.1	-3.21414	3.32E-06	RNA, U2 small nuclear 2, pseudogene [Source:HGNC Symbol;Acc:HGNC:10152]	chr11	-
[80]	PRC1-AS1	ENSG00000258725.2	-3.19542	1.42E-10	PRC1 antisense RNA 1 [Source:HGNC Symbol;Acc:HGNC:48587]	chr15	+

[81]	AC004980.1	ENSG00000205485.13	-3.18982	5.12E-06	pseudogene similar to part of postmeiotic segregation increased 2-like 3 PMS2L3	chr7	+
[82]	PLK1	ENSG00000166851.15	-3.17067	5.20E-219	polo like kinase 1 [Source:HGNC Symbol;Acc:HGNC:9077]	chr16	+
[83]	KIFC1	ENSG00000237649.8	-3.16217	2.02E-21	kinesin family member C1 [Source:HGNC Symbol;Acc:HGNC:6389]	chr6	+
[84]	SNORA70	ENSG00000207165.1	-3.16061	2.27E-08	small nucleolar RNA, H/ACA box 70 [Source:HGNC Symbol;Acc:HGNC:10231]	chrX	+
[85]	HSPB6	ENSG00000004776.13	-3.15932	4.88E-06	heat shock protein family B (small) member 6 [Source:HGNC Symbol;Acc:HGNC:26511]	chr19	-
[86]	KIF18B	ENSG00000186185.14	-3.15653	8.14E-90	kinesin family member 18B [Source:HGNC Symbol;Acc:HGNC:27102]	chr17	-
[87]	HIST2H4A	ENSG00000270882.2	-3.15097	0	H4 clustered histone 14 [Source:HGNC Symbol;Acc:HGNC:4794]	chr1	+
[88]	HIST1H2BB	ENSG00000276410.3	-3.14803	9.26E-98	H2B clustered histone 3 [Source:HGNC Symbol;Acc:HGNC:4751]	chr6	-
[89]	AP001092.1	ENSG00000237410.1	-3.13451	5.97E-06	NRXN2 antisense RNA 1 [Source:HGNC Symbol;Acc:HGNC:40416]	chr11	+
[90]	HIST1H2BF	ENSG00000277224.2	-3.13145	2.14E-126	H2B clustered histone 7 [Source:HGNC Symbol;Acc:HGNC:4752]	chr6	+
[91]	MT-TA	ENSG00000210127.1	-3.12924	7.95E-19	mitochondrially encoded tRNA-Ala (GCN) [Source:HGNC Symbol;Acc:HGNC:7475]	chrM	-
[92]	HIST1H1D	ENSG00000124575.6	-3.11859	3.07E-267	H1.3 linker histone, cluster member [Source:HGNC Symbol;Acc:HGNC:4717]	chr6	-
[93]	CU633906.2	ENSG00000278903.3	-3.11689	6.76E-06	novel transcript	chr21	+
[94]	HIST2H4B	ENSG00000270276.2	-3.11243	0	H4 clustered histone 15 [Source:HGNC Symbol;Acc:HGNC:29607]	chr1	-
[95]	SNORA73A	ENSG00000274266.1	-3.08779	3.69E-227	small nucleolar RNA, H/ACA box 73A [Source:HGNC Symbol;Acc:HGNC:10115]	chr1	+
[96]	PRR11	ENSG00000068489.12	-3.08387	1.88E-121	proline rich 11 [Source:HGNC Symbol;Acc:HGNC:25619]	chr17	+
[97]	RN7SL342P	ENSG00000239398.3	-3.08041	2.62E-07	RNA, 7SL, cytoplasmic 342, pseudogene [Source:HGNC Symbol;Acc:HGNC:46358]	chr18	+

[98]	HJURP	ENSG00000123485.12	-3.0748	3.95E-200	Holliday junction recognition protein [Source:HGNC Symbol;Acc:HGNC:25444]	chr2	-
[99]	RNU5E-1	ENSG00000199347.1	-3.06963	4.06E-08	RNA, U5E small nuclear 1 [Source:HGNC Symbol;Acc:HGNC:10215]	chr1	+
[100]	HIST1H2AG	ENSG00000196787.3	-3.06878	1.30E-246	H2A clustered histone 11 [Source:HGNC Symbol;Acc:HGNC:4737]	chr6	+
[101]	TSSK5P	ENSG00000227473.1	-3.0571	1.21E-05	testis specific serine kinase 5, pseudogene [Source:HGNC Symbol;Acc:HGNC:31931]	chr8	-
[102]	SCARNA22	ENSG00000249784.1	-3.05367	2.80E-07	small Cajal body-specific RNA 22 [Source:HGNC Symbol;Acc:HGNC:32580]	chr4	+
[103]	PPFIA2	ENSG00000139220.16	-3.05074	3.58E-06	PTPRF interacting protein alpha 2 [Source:HGNC Symbol;Acc:HGNC:9246]	chr12	-
[104]	AC055736.1	ENSG00000257500.1	-3.04448	1.33E-05	novel transcript, antisense to KRT75 and KRT82	chr12	+
[105]	FP671120.2	ENSG00000277437.1	-3.03865	2.78E-09	nan	chr21	+
[106]	HIST1H2BO	ENSG00000274641.1	-3.03279	6.23E-141	H2B clustered histone 17 [Source:HGNC Symbol;Acc:HGNC:4758]	chr6	+
[107]	AC009119.1	ENSG00000260228.6	-3.02904	7.90E-06	novel transcript, antisense to CDH13	chr16	-
[108]	HIST1H3A	ENSG00000275714.1	-3.02712	3.32E-65	H3 clustered histone 1 [Source:HGNC Symbol;Acc:HGNC:4766]	chr6	+
[109]	MKI67	ENSG00000148773.14	-3.02164	0	marker of proliferation Ki-67 [Source:HGNC Symbol;Acc:HGNC:7107]	chr10	-
[110]	HIST1H2BL	ENSG00000185130.5	-3.01734	7.02E-58	H2B clustered histone 13 [Source:HGNC Symbol;Acc:HGNC:4748]	chr6	-
[111]	TERT	ENSG00000164362.20	-3.015	1.63E-05	telomerase reverse transcriptase [Source:HGNC Symbol;Acc:HGNC:11730]	chr5	-
[112]	CR392039.1	ENSG00000264063.1	-3.01096	4.13E-09	nan	chr21	+
[113]	SH3BP1	ENSG00000100092.23	-2.98745	1.93E-10	SH3 domain binding protein 1 [Source:HGNC Symbol;Acc:HGNC:10824]	chr22	+

[114]	KIF20A	ENSG00000112984.12	-2.98637	5.51E-175	kinesin family member 20A [Source:HGNC Symbol;Acc:HGNC:9787]	chr5	+
[115]	HIST1H4B	ENSG00000278705.1	-2.98636	4.95E-233	H4 clustered histone 2 [Source:HGNC Symbol;Acc:HGNC:4789]	chr6	-
[116]	UHRF1	ENSG00000276043.5	-2.9758	2.01E-137	ubiquitin like with PHD and ring finger domains 1 [Source:HGNC Symbol;Acc:HGNC:12556]	chr19	+
[117]	SPC25	ENSG00000152253.9	-2.93556	1.76E-17	SPC25 component of NDC80 kinetochore complex [Source:HGNC Symbol;Acc:HGNC:24031]	chr2	-
[118]	POLA2	ENSG00000014138.9	-2.92088	2.67E-30	DNA polymerase alpha 2, accessory subunit [Source:HGNC Symbol;Acc:HGNC:30073]	chr11	+
[119]	PLSCR3	ENSG00000187838.17	-2.91791	3.36E-16	phospholipid scramblase 3 [Source:HGNC Symbol;Acc:HGNC:16495]	chr17	-
[120]	ARL17B	ENSG00000228696.9	-2.91327	3.94E-05	ADP ribosylation factor like GTPase 17B [Source:HGNC Symbol;Acc:HGNC:32387]	chr17	-
[121]	AC051619.7	ENSG00000259932.1	-2.91071	4.68E-06	novel transcript	chr15	-
[122]	RNU6ATAC	ENSG00000221676.1	-2.90805	4.14E-06	RNA, U6atac small nuclear (U12-dependent splicing) [Source:HGNC Symbol;Acc:HGNC:34017]	chr9	-
[123]	ANLN	ENSG00000011426.11	-2.89896	0	anillin actin binding protein [Source:HGNC Symbol;Acc:HGNC:14082]	chr7	+
[124]	SNORA52	ENSG00000199785.1	-2.89759	3.67E-07	small nucleolar RNA, H/ACA box 52 [Source:HGNC Symbol;Acc:HGNC:32645]	chr11	+
[125]	AP001042.1	ENSG00000205622.11	-2.88655	4.55E-05	novel transcript	chr21	-
[126]	RN7SL823P	ENSG00000263595.2	-2.88177	6.69E-12	RNA, 7SL, cytoplasmic 823, pseudogene [Source:HGNC Symbol;Acc:HGNC:46839]	chr19	-
[127]	HIST1H3C	ENSG00000287080.1	-2.88176	4.66E-242	H3 clustered histone 3 [Source:HGNC Symbol;Acc:HGNC:4768]	chr6	+
[128]	HIST1H2AE	ENSG00000277075.2	-2.87113	9.78E-152	H2A clustered histone 8 [Source:HGNC Symbol;Acc:HGNC:4724]	chr6	+

[129]	HAS1	ENSG00000105509.10	-2.87033	2.45E-151	hyaluronan synthase 1 [Source:HGNC Symbol;Acc:HGNC:4818]	chr19	-
[130]	CNIH2	ENSG00000174871.11	-2.83157	9.73E-06	cornichon family AMPA receptor auxiliary protein 2 [Source:HGNC Symbol;Acc:HGNC:28744]	chr11	+
[131]	LMNB1	ENSG00000113368.12	-2.82902	1.54E-90	lamin B1 [Source:HGNC Symbol;Acc:HGNC:6637]	chr5	+
[132]	SHCBP1	ENSG00000171241.9	-2.82648	7.39E-171	SHC binding and spindle associated 1 [Source:HGNC Symbol;Acc:HGNC:29547]	chr16	-
[133]	RNY1	ENSG00000201098.1	-2.82472	9.16E-08	RNA, Ro60-associated Y1 [Source:HGNC Symbol;Acc:HGNC:10242]	chr7	-
[134]	WDR90	ENSG00000161996.19	-2.81571	8.71E-32	WD repeat domain 90 [Source:HGNC Symbol;Acc:HGNC:26960]	chr16	+
[135]	NFATC4	ENSG00000100968.14	-2.81357	2.13E-06	nuclear factor of activated T cells 4 [Source:HGNC Symbol;Acc:HGNC:7778]	chr14	+
[136]	KCNJ8	ENSG00000121361.5	-2.80854	8.80E-05	potassium inwardly rectifying channel subfamily J member 8 [Source:HGNC Symbol;Acc:HGNC:6269]	chr12	-
[137]	HIF1A-AS3	ENSG00000258667.2	-2.79822	9.23E-05	HIF1A antisense RNA 3 [Source:HGNC Symbol;Acc:HGNC:54284]	chr14	-
[138]	AC010422.3	ENSG00000269242.1	-2.78553	2.73E-11	novel transcript	chr19	-
[139]	TLCD3B	ENSG00000149926.13	-2.78339	0.000107	TLC domain containing 3B [Source:HGNC Symbol;Acc:HGNC:25295]	chr16	-
[140]	GREM2	ENSG00000180875.5	-2.78022	0.000104	gremlin 2, DAN family BMP antagonist [Source:HGNC Symbol;Acc:HGNC:17655]	chr1	-
[141]	RN7SL396P	ENSG00000244642.3	-2.77819	0	RNA, 7SL, cytoplasmic 396, pseudogene [Source:HGNC Symbol;Acc:HGNC:46412]	chr8	+
[142]	DLGAP5	ENSG00000126787.13	-2.77807	1.16E-205	DLG associated protein 5 [Source:HGNC Symbol;Acc:HGNC:16864]	chr14	-
[143]	AC211433.1	ENSG00000232729.8	-2.77702	0.00011	novel transcript, antisense to GTF2I	chr7	-

[144]	FOXC2-AS1	ENSG00000260944.1	-2.77653	0.000104	FOXC2 antisense RNA 1 [Source:HGNC Symbol;Acc:HGNC:50665]	chr16	-
[145]	SERTM2	ENSG00000260802.1	-2.77609	1.75E-05	serine rich and transmembrane domain containing 2 [Source:HGNC Symbol;Acc:HGNC:48576]	chrX	+
[146]	HIST1H2BH	ENSG00000275713.2	-2.76478	3.69E-206	H2B clustered histone 9 [Source:HGNC Symbol;Acc:HGNC:4755]	chr6	+
[147]	CDCA8	ENSG00000134690.11	-2.76171	2.14E-137	cell division cycle associated 8 [Source:HGNC Symbol;Acc:HGNC:14629]	chr1	+
[148]	INSYN2B	ENSG00000204767.4	-2.75948	1.13E-99	inhibitory synaptic factor family member 2B [Source:HGNC Symbol;Acc:HGNC:37271]	chr5	-
[149]	RN7SL217P	ENSG00000264706.2	-2.75907	9.00E-08	RNA, 7SL, cytoplasmic 217, pseudogene [Source:HGNC Symbol;Acc:HGNC:46233]	chr3	-
[150]	SLC14A1	ENSG00000141469.18	-2.75716	0.00012	solute carrier family 14 member 1 (Kidd blood group) [Source:HGNC Symbol;Acc:HGNC:10918]	chr18	+
[151]	C10orf55	ENSG00000222047.8	-2.75247	1.75E-15	chromosome 10 putative open reading frame 55 [Source:HGNC Symbol;Acc:HGNC:31008]	chr10	-
[152]	NCAPH	ENSG00000121152.10	-2.74983	2.23E-135	non-SMC condensin I complex subunit H [Source:HGNC Symbol;Acc:HGNC:1112]	chr2	+
[153]	TOP2A	ENSG00000131747.15	-2.74719	0	DNA topoisomerase II alpha [Source:HGNC Symbol;Acc:HGNC:11989]	chr17	-
[154]	RN7SL151P	ENSG00000244230.3	-2.73451	6.70E-12	RNA, 7SL, cytoplasmic 151, pseudogene [Source:HGNC Symbol;Acc:HGNC:46167]	chr9	+
[155]	EPSTI1	ENSG00000133106.14	-2.73306	0.000143	epithelial stromal interaction 1 [Source:HGNC Symbol;Acc:HGNC:16465]	chr13	-
[156]	CDC20	ENSG00000117399.14	-2.72969	7.87E-194	cell division cycle 20 [Source:HGNC Symbol;Acc:HGNC:1723]	chr1	+
[157]	Z94721.3	ENSG00000285730.1	-2.72171	0.000153	novel transcript	chr6	-
[158]	WNT5A-AS1	ENSG00000244586.1	-2.71633	1.57E-06	WNT5A antisense RNA 1 [Source:HGNC Symbol;Acc:HGNC:40616]	chr3	+

[159]	NUF2	ENSG00000143228.13	-2.71306	6.69E-70	NUF2 component of NDC80 kinetochore complex [Source:HGNC Symbol;Acc:HGNC:14621]	chr1	+
[160]	HGF	ENSG00000019991.18	-2.70999	2.98E-93	hepatocyte growth factor [Source:HGNC Symbol;Acc:HGNC:4893]	chr7	-
[161]	GDF5	ENSG00000125965.9	-2.70432	1.13E-29	growth differentiation factor 5 [Source:HGNC Symbol;Acc:HGNC:4220]	chr20	-
[162]	MIR3651	ENSG00000281156.1	-2.70147	8.79E-06	microRNA 3651 [Source:HGNC Symbol;Acc:HGNC:38897]	chr9	-
[163]	RN7SL366P	ENSG00000266128.2	-2.69153	5.59E-20	RNA, 7SL, cytoplasmic 366, pseudogene [Source:HGNC Symbol;Acc:HGNC:46382]	chr6	+
[164]	HIST1H4D	ENSG00000277157.1	-2.69039	1.87E-119	H4 clustered histone 4 [Source:HGNC Symbol;Acc:HGNC:4782]	chr6	-
[165]	KIF23	ENSG00000137807.15	-2.66794	5.41E-281	kinesin family member 23 [Source:HGNC Symbol;Acc:HGNC:6392]	chr15	+
[166]	MT-ND3	ENSG00000198840.2	-2.66624	1.64E-109	mitochondrially encoded NADH:ubiquinone oxidoreductase core subunit 3 [Source:HGNC Symbol;Acc:HGNC:7458]	chrM	+
[167]	CDCA7	ENSG00000144354.14	-2.66078	1.03E-42	cell division cycle associated 7 [Source:HGNC Symbol;Acc:HGNC:14628]	chr2	+
[168]	ADGRF2	ENSG00000164393.8	-2.65953	1.17E-05	adhesion G protein-coupled receptor F2 [Source:HGNC Symbol;Acc:HGNC:18991]	chr6	+
[169]	FAM110A	ENSG00000125898.13	-2.65745	5.20E-07	family with sequence similarity 110 member A [Source:HGNC Symbol;Acc:HGNC:16188]	chr20	+
[170]	CALML4	ENSG00000129007.14	-2.65275	6.30E-05	calmodulin like 4 [Source:HGNC Symbol;Acc:HGNC:18445]	chr15	-
[171]	SNORA79B	ENSG00000222489.1	-2.65037	2.71E-05	small nucleolar RNA, H/ACA box 79B [Source:HGNC Symbol;Acc:HGNC:52222]	chr14	-
[172]	HIST1H4C	ENSG00000197061.4	-2.64899	9.40E-192	H4 clustered histone 3 [Source:HGNC Symbol;Acc:HGNC:4787]	chr6	+
[173]	U2	ENSG00000274452.1	-2.64552	0.00026	U2 spliceosomal RNA [Source:RFAM;Acc:RF00004]	chr17	-

[174]	CDC20P1	ENSG00000231007.5	-2.64409	1.46E-20	cell division cycle 20 pseudogene 1 [Source:HGNC Symbol;Acc:HGNC:29487]	chr9	+
[175]	GTSE1	ENSG00000075218.19	-2.64116	1.18E-88	G2 and S-phase expressed 1 [Source:HGNC Symbol;Acc:HGNC:13698]	chr22	+
[176]	RN7SL734P	ENSG00000243420.3	-2.63932	0.000131	RNA, 7SL, cytoplasmic 734, pseudogene [Source:HGNC Symbol;Acc:HGNC:46750]	chr12	-
[177]	AC112777.1	ENSG00000256663.1	-2.63569	3.35E-41	ubiquitin-like with PHD and ring finger domains 1 (UHRF1) pseudogene	chr12	+
[178]	CCNA2	ENSG00000145386.10	-2.63378	2.13E-220	cyclin A2 [Source:HGNC Symbol;Acc:HGNC:1578]	chr4	-
[179]	NRM	ENSG00000137404.14	-2.63145	3.16E-05	nurim [Source:HGNC Symbol;Acc:HGNC:8003]	chr6	-
[180]	PALMD	ENSG00000099260.11	-2.62634	1.26E-135	palmdelphin [Source:HGNC Symbol;Acc:HGNC:15846]	chr1	+
[181]	TPM1-AS	ENSG00000259498.1	-2.6257	9.90E-11	TPM1 antisense RNA [Source:HGNC Symbol;Acc:HGNC:53635]	chr15	-
[182]	APLN	ENSG00000171388.12	-2.62259	2.71E-22	apelin [Source:HGNC Symbol;Acc:HGNC:16665]	chrX	-
[183]	PKMYT1	ENSG00000127564.17	-2.62252	2.28E-37	protein kinase, membrane associated tyrosine/threonine 1 [Source:HGNC Symbol;Acc:HGNC:29650]	chr16	-
[184]	RN7SL3	ENSG00000278771.1	-2.60583	0	RNA component of signal recognition particle 7SL3 [Source:HGNC Symbol;Acc:HGNC:23135]	chr14	-
[185]	BUB1	ENSG00000169679.15	-2.60346	2.69E-225	BUB1 mitotic checkpoint serine/threonine kinase [Source:HGNC Symbol;Acc:HGNC:1148]	chr2	-
[186]	CENPE	ENSG00000138778.12	-2.60046	0	centromere protein E [Source:HGNC Symbol;Acc:HGNC:1856]	chr4	-
[187]	UBE2C	ENSG00000175063.17	-2.59985	1.66E-224	ubiquitin conjugating enzyme E2 C [Source:HGNC Symbol;Acc:HGNC:15937]	chr20	+
[188]	ASTN2-AS1	ENSG00000229105.1	-2.59882	0.00035	ASTN2 antisense RNA 1 [Source:HGNC Symbol;Acc:HGNC:51175]	chr9	+

[189]	AC005586.1	ENSG00000240449.1	-2.59351	0.000369	REPIN1 antisense RNA 1 [Source:HGNC Symbol;Acc:HGNC:41201]	chr7	-
[190]	RN7SL77P	ENSG00000240837.3	-2.58789	1.52E-07	RNA, 7SL, cytoplasmic 77, pseudogene [Source:HGNC Symbol;Acc:HGNC:46093]	chr14	+
[191]	SNORA71A	ENSG00000225091.3	-2.58624	2.07E-06	small nucleolar RNA, H/ACA box 71A [Source:HGNC Symbol;Acc:HGNC:10232]	chr20	-
[192]	AC099850.3	ENSG00000265415.1	-2.5781	1.27E-14	novel transcript, antisense to PRR11	chr17	-
[193]	HMMR	ENSG00000072571.20	-2.57793	3.15E-151	hyaluronan mediated motility receptor [Source:HGNC Symbol;Acc:HGNC:5012]	chr5	+
[194]	HIST1H2AM	ENSG00000278677.1	-2.57548	1.20E-143	H2A clustered histone 17 [Source:HGNC Symbol;Acc:HGNC:4735]	chr6	-
[195]	RN7SL5P	ENSG00000265735.2	-2.56252	0	RNA, 7SL, cytoplasmic 5, pseudogene [Source:HGNC Symbol;Acc:HGNC:10040]	chr9	+
[196]	KIF2C	ENSG00000142945.13	-2.56156	2.83E-173	kinesin family member 2C [Source:HGNC Symbol;Acc:HGNC:6393]	chr1	+
[197]	NEURL1B	ENSG00000214357.9	-2.55977	8.84E-56	neuralized E3 ubiquitin protein ligase 1B [Source:HGNC Symbol;Acc:HGNC:35422]	chr5	+
[198]	HIST1H2BE	ENSG00000274290.2	-2.55352	4.36E-72	H2B clustered histone 6 [Source:HGNC Symbol;Acc:HGNC:4753]	chr6	+
[199]	RN7SL4P	ENSG00000263740.2	-2.54139	0	RNA, 7SL, cytoplasmic 4, pseudogene [Source:HGNC Symbol;Acc:HGNC:10039]	chr3	+
[200]	E2F8	ENSG00000129173.13	-2.53701	2.05E-55	E2F transcription factor 8 [Source:HGNC Symbol;Acc:HGNC:24727]	chr11	-
[201]	CCDC107	ENSG00000159884.12	-2.52495	8.11E-05	coiled-coil domain containing 107 [Source:HGNC Symbol;Acc:HGNC:28465]	chr9	+
[202]	LINC01085	ENSG00000248698.6	-2.52461	3.32E-15	long intergenic non-protein coding RNA 1085 [Source:HGNC Symbol;Acc:HGNC:27198]	chr4	+
[203]	RN7SKP175	ENSG00000275558.1	-2.52197	4.39E-19	RN7SK pseudogene 175 [Source:HGNC Symbol;Acc:HGNC:45899]	chr8	+

[204]	FAM83D	ENSG00000101447.15	-2.5116	1.75E-127	family with sequence similarity 83 member D [Source:HGNC Symbol;Acc:HGNC:16122]	chr20	+
[205]	NDC80	ENSG00000080986.13	-2.50818	3.61E-121	NDC80 kinetochore complex component [Source:HGNC Symbol;Acc:HGNC:16909]	chr18	+
[206]	RN7SL472P	ENSG00000240457.3	-2.50607	1.73E-13	RNA, 7SL, cytoplasmic 472, pseudogene [Source:HGNC Symbol;Acc:HGNC:46488]	chr14	-
[207]	KIF14	ENSG00000118193.12	-2.4993	4.13E-152	kinesin family member 14 [Source:HGNC Symbol;Acc:HGNC:19181]	chr1	-
[208]	AURKB	ENSG00000178999.13	-2.49812	1.11E-71	aurora kinase B [Source:HGNC Symbol;Acc:HGNC:11390]	chr17	-
[209]	RN7SL665P	ENSG00000264169.2	-2.4957	7.28E-09	RNA, 7SL, cytoplasmic 665, pseudogene [Source:HGNC Symbol;Acc:HGNC:46681]	chr9	+
[210]	HIST1H2BN	ENSG00000233822.4	-2.4919	3.75E-154	H2B clustered histone 15 [Source:HGNC Symbol;Acc:HGNC:4749]	chr6	+
[211]	AHRR	ENSG00000063438.17	-2.49069	7.68E-08	aryl-hydrocarbon receptor repressor [Source:HGNC Symbol;Acc:HGNC:346]	chr5	+
[212]	DMBX1	ENSG00000197587.10	-2.48493	0.000222	diencephalon/mesencephalon homeobox 1 [Source:HGNC Symbol;Acc:HGNC:19026]	chr1	+
[213]	RN7SL735P	ENSG00000244044.3	-2.47976	2.85E-09	RNA, 7SL, cytoplasmic 735, pseudogene [Source:HGNC Symbol;Acc:HGNC:46751]	chr17	+
[214]	7SK	ENSG00000271394.1	-2.47772	0	7SK RNA [Source:RFAM;Acc:RF00100]	chr17	+
[215]	RPSAP52	ENSG00000241749.4	-2.47653	8.31E-39	ribosomal protein SA pseudogene 52 [Source:HGNC Symbol;Acc:HGNC:35752]	chr12	-
[216]	NCAPG	ENSG00000109805.10	-2.47341	2.14E-300	non-SMC condensin I complex subunit G [Source:HGNC Symbol;Acc:HGNC:24304]	chr4	+
[217]	CENPF	ENSG00000117724.13	-2.47284	0	centromere protein F [Source:HGNC Symbol;Acc:HGNC:1857]	chr1	+
[218]	HSP90AA5P	ENSG00000205955.4	-2.47193	0.000786	heat shock protein 90 alpha family class A member 5, pseudogene [Source:HGNC Symbol;Acc:HGNC:32535]	chr3	+

[219]	BUB1B	ENSG00000156970.12	-2.47177	1.40E-182	BUB1 mitotic checkpoint serine/threonine kinase B [Source:HGNC Symbol;Acc:HGNC:1149]	chr15	+
[220]	TTK	ENSG00000112742.10	-2.46797	2.87E-75	TTK protein kinase [Source:HGNC Symbol;Acc:HGNC:12401]	chr6	+
[221]	TROAP	ENSG00000135451.13	-2.46695	2.33E-62	trophinin associated protein [Source:HGNC Symbol;Acc:HGNC:12327]	chr12	+
[222]	KIF15	ENSG00000163808.17	-2.45934	2.47E-50	kinesin family member 15 [Source:HGNC Symbol;Acc:HGNC:17273]	chr3	+
[223]	NDP	ENSG00000124479.11	-2.45659	1.00E-41	norrin cystine knot growth factor NDP [Source:HGNC Symbol;Acc:HGNC:7678]	chrX	-
[224]	HIST1H4H	ENSG00000158406.4	-2.45528	2.73E-149	H4 clustered histone 8 [Source:HGNC Symbol;Acc:HGNC:4788]	chr6	-
[225]	NEXN	ENSG00000162614.18	-2.45088	5.67E-08	nexilin F-actin binding protein [Source:HGNC Symbol;Acc:HGNC:29557]	chr1	+
[226]	GINS1	ENSG00000101003.10	-2.44746	5.20E-35	GINS complex subunit 1 [Source:HGNC Symbol;Acc:HGNC:28980]	chr20	+
[227]	RN7SL660P	ENSG00000278746.1	-2.44632	1.13E-07	RNA, 7SL, cytoplasmic 660, pseudogene [Source:HGNC Symbol;Acc:HGNC:46676]	chr14	+
[228]	H2AFZP1	ENSG00000213440.2	-2.44574	0.001055	H2A.Z histone pseudogene 1 [Source:HGNC Symbol;Acc:HGNC:4742]	chr21	-
[229]	TMEM160	ENSG00000130748.7	-2.44556	1.73E-09	transmembrane protein 160 [Source:HGNC Symbol;Acc:HGNC:26042]	chr19	-
[230]	RN7SL1	ENSG00000276168.1	-2.43939	0	RNA component of signal recognition particle 7SL1 [Source:HGNC Symbol;Acc:HGNC:10038]	chr14	+
[231]	CCNB1	ENSG00000134057.15	-2.4293	0	cyclin B1 [Source:HGNC Symbol;Acc:HGNC:1579]	chr5	+
[232]	MIR3648-2	ENSG00000264462.1	-2.42527	5.28E-08	microRNA 3648-2 [Source:HGNC Symbol;Acc:HGNC:50843]	chr21	+
[233]	MIR3648-1	ENSG00000275708.1	-2.42527	5.28E-08	microRNA 3648-1 [Source:HGNC Symbol;Acc:HGNC:38941]	chr21	+

[234]	SNORD94	ENSG00000208772.1	-2.42239	0.000619	small nucleolar RNA, C/D box 94 [Source:HGNC Symbol;Acc:HGNC:32756]	chr2	+
[235]	NES	ENSG00000132688.11	-2.42141	3.73E-27	nestin [Source:HGNC Symbol;Acc:HGNC:7756]	chr1	-
[236]	PRSS12	ENSG00000164099.3	-2.42098	3.44E-05	serine protease 12 [Source:HGNC Symbol;Acc:HGNC:9477]	chr4	-
[237]	ACSBG2	ENSG00000130377.13	-2.42041	0.001085	acyl-CoA synthetase bubblegum family member 2 [Source:HGNC Symbol;Acc:HGNC:24174]	chr19	+
[238]	PGGHG	ENSG00000142102.16	-2.41405	0.000357	protein-glucosylgalactosylhydroxylysine glucosidase [Source:HGNC Symbol;Acc:HGNC:26210]	chr11	+
[239]	BMS1P1	ENSG00000204177.10	-2.41194	0.001261	BMS1 pseudogene 1 [Source:HGNC Symbol;Acc:HGNC:23649]	chr10	+
[240]	TPX2	ENSG00000088325.16	-2.40914	0	TPX2 microtubule nucleation factor [Source:HGNC Symbol;Acc:HGNC:1249]	chr20	+
[241]	SNORA73B	ENSG00000200087.1	-2.40883	6.82E-239	small nucleolar RNA, H/ACA box 73B [Source:HGNC Symbol;Acc:HGNC:10116]	chr1	+
[242]	INCENP	ENSG00000149503.13	-2.40868	1.40E-123	inner centromere protein [Source:HGNC Symbol;Acc:HGNC:6058]	chr11	+
[243]	CFAP299	ENSG00000197826.11	-2.40827	0.001167	cilia and flagella associated protein 299 [Source:HGNC Symbol;Acc:HGNC:28554]	chr4	+
[244]	CA9	ENSG00000107159.13	-2.40714	1.55E-28	carbonic anhydrase 9 [Source:HGNC Symbol;Acc:HGNC:1383]	chr9	+
[245]	RN7SL518P	ENSG00000265486.2	-2.40604	1.38E-05	RNA, 7SL, cytoplasmic 518, pseudogene [Source:HGNC Symbol;Acc:HGNC:46534]	chr10	-
[246]	PLK4	ENSG00000142731.11	-2.4056	2.81E-78	polo like kinase 4 [Source:HGNC Symbol;Acc:HGNC:11397]	chr4	+
[247]	CENPI	ENSG00000102384.13	-2.40484	2.09E-70	centromere protein I [Source:HGNC Symbol;Acc:HGNC:3968]	chrX	+
[248]	KIF11	ENSG00000138160.6	-2.40215	7.17E-262	kinesin family member 11 [Source:HGNC Symbol;Acc:HGNC:6388]	chr10	+

[249]	AC091057.1	ENSG00000187951.11	-2.40097	1.54E-16	novel transcript	chr15	+
[250]	DEPDC1	ENSG00000024526.17	-2.3987	9.58E-181	DEP domain containing 1 [Source:HGNC Symbol;Acc:HGNC:22949]	chr1	-
[251]	CKAP2L	ENSG00000169607.13	-2.39839	2.11E-171	cytoskeleton associated protein 2 like [Source:HGNC Symbol;Acc:HGNC:26877]	chr2	-
[252]	CLSPN	ENSG00000092853.14	-2.39503	5.34E-141	claspin [Source:HGNC Symbol;Acc:HGNC:19715]	chr1	-
[253]	MCM10	ENSG00000065328.16	-2.39022	1.87E-74	minichromosome maintenance 10 replication initiation factor [Source:HGNC Symbol;Acc:HGNC:18043]	chr10	+
[254]	RN7SL546P	ENSG00000239224.3	-2.38345	2.21E-05	RNA, 7SL, cytoplasmic 546, pseudogene [Source:HGNC Symbol;Acc:HGNC:46562]	chr14	-
[255]	HIST2H2BE	ENSG00000184678.10	-2.38248	2.08E-291	H2B clustered histone 21 [Source:HGNC Symbol;Acc:HGNC:4760]	chr1	-
[256]	ELN-AS1	ENSG00000232415.1	-2.37981	0.001393	ELN antisense RNA 1 [Source:HGNC Symbol;Acc:HGNC:40212]	chr7	-
[257]	LCTL	ENSG00000188501.11	-2.37969	0.000323	lactase like [Source:HGNC Symbol;Acc:HGNC:15583]	chr15	-
[258]	IQGAP3	ENSG00000183856.11	-2.37542	3.29E-94	IQ motif containing GTPase activating protein 3 [Source:HGNC Symbol;Acc:HGNC:20669]	chr1	-
[259]	RN7SL645P	ENSG00000240863.3	-2.37086	0.000135	RNA, 7SL, cytoplasmic 645, pseudogene [Source:HGNC Symbol;Acc:HGNC:46661]	chr16	-
[260]	RN7SKP203	ENSG00000200488.1	-2.36877	0	RN7SK pseudogene 203 [Source:HGNC Symbol;Acc:HGNC:45927]	chr2	-
[261]	HAS2	ENSG00000170961.7	-2.36433	6.04E-10	hyaluronan synthase 2 [Source:HGNC Symbol;Acc:HGNC:4819]	chr8	-
[262]	CENPA	ENSG00000115163.15	-2.36139	8.02E-39	centromere protein A [Source:HGNC Symbol;Acc:HGNC:1851]	chr2	+
[263]	PSRC1	ENSG00000134222.16	-2.36072	3.30E-61	proline and serine rich coiled-coil 1 [Source:HGNC Symbol;Acc:HGNC:24472]	chr1	-

[264]	RN7SL2	ENSG00000274012.1	-2.35983	0	RNA component of signal recognition particle 7SL2 [Source:HGNC Symbol;Acc:HGNC:23134]	chr14	-
[265]	U1	ENSG00000274210.1	-2.35952	1.10E-06	RNA, variant U1 small nuclear 27 [Source:HGNC Symbol;Acc:HGNC:54431]	chr1	+
[266]	AL591684.2	ENSG00000254929.6	-2.35794	0.001671	novel transcript	chr10	-
[267]	RN7SL113P	ENSG00000278590.1	-2.35567	0.001592	RNA, 7SL, cytoplasmic 113, pseudogene [Source:HGNC Symbol;Acc:HGNC:46129]	chr2	+
[268]	CDCA3	ENSG00000111665.11	-2.35516	2.30E-49	cell division cycle associated 3 [Source:HGNC Symbol;Acc:HGNC:14624]	chr12	-
[269]	RN7SKP255	ENSG00000200312.1	-2.35403	0	RN7SK pseudogene 255 [Source:HGNC Symbol;Acc:HGNC:45979]	chr14	+
[270]	RRM2	ENSG00000171848.15	-2.35169	0	ribonucleotide reductase regulatory subunit M2 [Source:HGNC Symbol;Acc:HGNC:10452]	chr2	+
[271]	MRM2	ENSG00000122687.18	-2.35116	0.001714	mitochondrial rRNA methyltransferase 2 [Source:HGNC Symbol;Acc:HGNC:16352]	chr7	-
[272]	ATG10	ENSG00000152348.16	-2.34652	5.90E-14	autophagy related 10 [Source:HGNC Symbol;Acc:HGNC:20315]	chr5	+
[273]	AC026951.1	ENSG00000259358.1	-2.33885	7.92E-11	DEP domain containing 1 pseudogene 1 [Source:HGNC Symbol;Acc:HGNC:31125]	chr15	-
[274]	ENO3	ENSG00000108515.17	-2.33736	9.54E-05	enolase 3 [Source:HGNC Symbol;Acc:HGNC:3354]	chr17	+
[275]	KCTD4	ENSG00000180332.6	-2.33525	5.22E-41	potassium channel tetramerization domain containing 4 [Source:HGNC Symbol;Acc:HGNC:23227]	chr13	-
[276]	EIF4A1P9	ENSG00000262953.1	-2.33316	0.001903	eukaryotic translation initiation factor 4A1 pseudogene 9 [Source:HGNC Symbol;Acc:HGNC:37928]	chr17	-
[277]	AC090498.1	ENSG00000279483.2	-2.33112	2.94E-18	ribosomal protein L41 (RPL41) pseudogene	chr7	+

[278]	TEDC1	ENSG00000185347.17	-2.3299	3.25E-28	tubulin epsilon and delta complex 1 [Source:HGNC Symbol;Acc:HGNC:20127]	chr14	+
[279]	AL589182.3	ENSG00000286614.1	-2.32921	0.001939	novel pseudogene	chr14	+
[280]	HIST2H2AB	ENSG00000184270.5	-2.32749	6.61E-56	H2A clustered histone 21 [Source:HGNC Symbol;Acc:HGNC:20508]	chr1	-
[281]	LINC00705	ENSG00000225269.3	-2.31909	0.001919	long intergenic non-protein coding RNA 705 [Source:HGNC Symbol;Acc:HGNC:27874]	chr10	+
[282]	SGO1	ENSG00000129810.15	-2.31656	3.42E-51	shugoshin 1 [Source:HGNC Symbol;Acc:HGNC:25088]	chr3	-
[283]	RPS10-NUDT3	ENSG00000270800.3	-2.31566	0.001445	RPS10-NUDT3 readthrough [Source:HGNC Symbol;Acc:HGNC:49181]	chr6	-
[284]	ARHGAP11A	ENSG00000198826.11	-2.31476	9.35E-116	Rho GTPase activating protein 11A [Source:HGNC Symbol;Acc:HGNC:15783]	chr15	+
[285]	AC005480.1	ENSG00000258891.1	-2.31199	4.89E-05	novel transcript, antisense to ZNF410	chr14	-
[286]	RN7SL752P	ENSG00000239437.3	-2.30511	1.56E-74	RNA, 7SL, cytoplasmic 752, pseudogene [Source:HGNC Symbol;Acc:HGNC:46768]	chr3	+
[287]	AC007750.1	ENSG00000236841.8	-2.30394	0.000543	novel transcript	chr2	+
[288]	OPN1SW	ENSG00000128617.2	-2.30333	0.002099	opsin 1, short wave sensitive [Source:HGNC Symbol;Acc:HGNC:1012]	chr7	-
[289]	FANCA	ENSG00000187741.15	-2.30214	1.15E-78	FA complementation group A [Source:HGNC Symbol;Acc:HGNC:3582]	chr16	-
[290]	AC231533.1	ENSG00000232828.1	-2.29955	0.002169	novel transcript	chrX	-
[291]	CDK1	ENSG00000170312.16	-2.29938	1.11E-208	cyclin dependent kinase 1 [Source:HGNC Symbol;Acc:HGNC:1722]	chr10	+
[292]	FBXO43	ENSG00000156509.14	-2.29815	1.78E-10	F-box protein 43 [Source:HGNC Symbol;Acc:HGNC:28521]	chr8	-
[293]	AC068831.7	ENSG00000284946.1	-2.2939	2.83E-05	novel protein	chr15	-

[294]	ORC1	ENSG00000085840.13	-2.2909	1.07E-63	origin recognition complex subunit 1 [Source:HGNC Symbol;Acc:HGNC:8487]	chr1	-
[295]	CHTF18	ENSG00000127586.17	-2.28825	3.15E-22	chromosome transmission fidelity factor 18 [Source:HGNC Symbol;Acc:HGNC:18435]	chr16	+
[296]	RAD54L	ENSG00000085999.13	-2.28468	1.02E-24	RAD54 like [Source:HGNC Symbol;Acc:HGNC:9826]	chr1	+
[297]	AC104852.1	ENSG00000237810.3	-2.28369	2.56E-06	polo-like kinase 1 (Drosophila) (PLK1) pseudogene	chr8	-
[298]	HIST1H2BG	ENSG00000273802.2	-2.28245	7.66E-157	H2B clustered histone 8 [Source:HGNC Symbol;Acc:HGNC:4746]	chr6	-
[299]	LINC02641	ENSG00000230131.6	-2.28171	0.002351	long intergenic non-protein coding RNA 2641 [Source:HGNC Symbol;Acc:HGNC:54125]	chr10	+
[300]	SPC24	ENSG00000161888.11	-2.28116	4.60E-82	SPC24 component of NDC80 kinetochore complex [Source:HGNC Symbol;Acc:HGNC:26913]	chr19	-
[301]	RN7SL706P	ENSG00000244677.3	-2.27971	1.41E-07	RNA, 7SL, cytoplasmic 706, pseudogene [Source:HGNC Symbol;Acc:HGNC:46722]	chr14	-
[302]	HIST2H2AA4	ENSG00000272196.2	-2.27402	0	H2A clustered histone 19 [Source:HGNC Symbol;Acc:HGNC:29668]	chr1	+
[303]	HIST2H2AA3	ENSG00000203812.2	-2.27375	0	H2A clustered histone 18 [Source:HGNC Symbol;Acc:HGNC:4736]	chr1	-
[304]	RNU5B-1	ENSG00000200156.1	-2.27068	1.40E-05	RNA, U5B small nuclear 1 [Source:HGNC Symbol;Acc:HGNC:10212]	chr15	+
[305]	SNORA61	ENSG00000278274.1	-2.26912	0.000773	small nucleolar RNA, H/ACA box 61 [Source:HGNC Symbol;Acc:HGNC:32655]	chr1	-
[306]	LINC01109	ENSG00000271167.1	-2.26677	5.47E-05	long intergenic non-protein coding RNA 1109 [Source:HGNC Symbol;Acc:HGNC:49235]	chr8	+
[307]	AC013643.2	ENSG00000253875.2	-2.26429	0.002628	novel transcript	chr8	+
[308]	PMF1-BGLAP	ENSG00000260238.6	-2.2616	0.002606	PMF1-BGLAP readthrough [Source:HGNC Symbol;Acc:HGNC:42953]	chr1	+

[309]	CARD16	ENSG00000204397.8	-2.25673	0.002707	caspase recruitment domain family member 16 [Source:HGNC Symbol;Acc:HGNC:33701]	chr11	-
[310]	SNORA49	ENSG00000208892.1	-2.25222	2.01E-06	small nucleolar RNA, H/ACA box 49 [Source:HGNC Symbol;Acc:HGNC:32642]	chr12	+
[311]	RNU5A-1	ENSG00000199568.1	-2.2494	9.38E-06	RNA, U5A small nuclear 1 [Source:HGNC Symbol;Acc:HGNC:10211]	chr15	+
[312]	PBK	ENSG00000168078.10	-2.24862	8.46E-150	PDZ binding kinase [Source:HGNC Symbol;Acc:HGNC:18282]	chr8	-
[313]	CIP2A	ENSG00000163507.14	-2.24753	9.26E-136	cellular inhibitor of PP2A [Source:HGNC Symbol;Acc:HGNC:29302]	chr3	-
[314]	KNL1	ENSG00000137812.19	-2.24644	2.18E-253	kinetochore scaffold 1 [Source:HGNC Symbol;Acc:HGNC:24054]	chr15	+
[315]	RN7SL346P	ENSG00000240470.3	-2.23981	0.002931	RNA, 7SL, cytoplasmic 346, pseudogene [Source:HGNC Symbol;Acc:HGNC:46362]	chr15	+
[316]	RPL41P1	ENSG00000227063.5	-2.23907	2.01E-19	ribosomal protein L41 pseudogene 1 [Source:HGNC Symbol;Acc:HGNC:10356]	chr20	+
[317]	POC1B-GALNT4	ENSG00000259075.6	-2.23418	0.003107	POC1B-GALNT4 readthrough [Source:HGNC Symbol;Acc:HGNC:42957]	chr12	-
[318]	MT-ATP8	ENSG00000228253.1	-2.23228	4.21E-83	mitochondrially encoded ATP synthase membrane subunit 8 [Source:HGNC Symbol;Acc:HGNC:7415]	chrM	+
[319]	COL1A2	ENSG00000164692.17	-2.23158	0	collagen type I alpha 2 chain [Source:HGNC Symbol;Acc:HGNC:2198]	chr7	+
[320]	LINC01723	ENSG00000234265.2	-2.22827	0.000807	long intergenic non-protein coding RNA 1723 [Source:HGNC Symbol;Acc:HGNC:52511]	chr20	+
[321]	RN7SKP9	ENSG00000201793.1	-2.22767	3.36E-246	RN7SK pseudogene 9 [Source:HGNC Symbol;Acc:HGNC:42627]	chr13	+
[322]	AC021127.1	ENSG00000248719.2	-2.22666	0.001369	putative novel transript	chr4	-
[323]	RN7SL507P	ENSG00000239702.3	-2.22569	2.05E-07	RNA, 7SL, cytoplasmic 507, pseudogene [Source:HGNC Symbol;Acc:HGNC:46523]	chr17	-

[324]	C3orf49	ENSG00000163632.14	-2.21635	0.003315	chromosome 3 open reading frame 49 [Source:HGNC Symbol;Acc:HGNC:25190]	chr3	+
[325]	AURKA	ENSG00000087586.17	-2.20796	3.87E-223	aurora kinase A [Source:HGNC Symbol;Acc:HGNC:11393]	chr20	-
[326]	KIF4A	ENSG00000090889.12	-2.20414	3.29E-117	kinesin family member 4A [Source:HGNC Symbol;Acc:HGNC:13339]	chrX	+
[327]	Metazoa_SRP	ENSG00000278701.1	-2.2031	0.002656	Metazoan signal recognition particle RNA [Source:RFAM;Acc:RF00017]	chr17	+
[328]	UBL7-AS1	ENSG00000247240.8	-2.19903	0.003674	UBL7 antisense RNA 1 (head to head) [Source:HGNC Symbol;Acc:HGNC:48614]	chr15	+
[329]	IFI44L	ENSG00000137959.16	-2.198	2.17E-26	interferon induced protein 44 like [Source:HGNC Symbol;Acc:HGNC:17817]	chr1	+
[330]	RNA5-8SN3	ENSG00000275215.1	-2.19616	0.000213	RNA, 5.8S ribosomal N3 [Source:HGNC Symbol;Acc:HGNC:53525]	chr21	+
[331]	5_8S_rRNA	ENSG00000277739.1	-2.19616	0.000213	5.8S ribosomal RNA [Source:RFAM;Acc:RF00002]	chr21	+
[332]	RNA5-8SN1	ENSG00000278189.1	-2.19616	0.000213	RNA, 5.8S ribosomal N1 [Source:HGNC Symbol;Acc:HGNC:53517]	chr21	+
[333]	RNA5-8SN2	ENSG00000278233.1	-2.19616	0.000213	RNA, 5.8S ribosomal N2 [Source:HGNC Symbol;Acc:HGNC:53521]	chr21	+
[334]	RN7SL97P	ENSG00000242216.3	-2.19614	0.000188	RNA, 7SL, cytoplasmic 97, pseudogene [Source:HGNC Symbol;Acc:HGNC:46113]	chr18	-
[335]	RN7SL795P	ENSG00000239748.3	-2.19208	0.002618	RNA, 7SL, cytoplasmic 795, pseudogene [Source:HGNC Symbol;Acc:HGNC:46811]	chr18	+
[336]	RN7SL415P	ENSG00000240961.3	-2.18991	6.28E-05	RNA, 7SL, cytoplasmic 415, pseudogene [Source:HGNC Symbol;Acc:HGNC:46431]	chr6	+
[337]	SNORA70	ENSG00000200237.1	-2.18554	3.31E-05	Small nucleolar RNA SNORA70 [Source:RFAM;Acc:RF00156]	chr19	+
[338]	CCNF	ENSG00000162063.13	-2.18272	3.79E-51	cyclin F [Source:HGNC Symbol;Acc:HGNC:1591]	chr16	+
[339]	FN1	ENSG00000115414.19	-2.18107	0	fibronectin 1 [Source:HGNC Symbol;Acc:HGNC:3778]	chr2	-

[340]	HIST1H4J	ENSG00000197238.4	-2.17991	7.86E-102	H4 clustered histone 11 [Source:HGNC Symbol;Acc:HGNC:4785]	chr6	+
[341]	RN7SL607P	ENSG00000278525.1	-2.17912	9.38E-06	RNA, 7SL, cytoplasmic 607, pseudogene [Source:HGNC Symbol;Acc:HGNC:46623]	chr20	-
[342]	HIST1H4K	ENSG00000273542.1	-2.17749	5.13E-101	H4 clustered histone 12 [Source:HGNC Symbol;Acc:HGNC:4784]	chr6	-
[343]	NUSAP1	ENSG00000137804.14	-2.17618	1.52E-08	nucleolar and spindle associated protein 1 [Source:HGNC Symbol;Acc:HGNC:18538]	chr15	+
[344]	ARAP1-AS2	ENSG00000245148.2	-2.17564	0.004067	ARAP1 antisense RNA 2 [Source:HGNC Symbol;Acc:HGNC:39994]	chr11	+
[345]	AC100871.1	ENSG00000250727.1	-2.17497	0.004081	neuropilin (NRP) and tolloid (TLL)-like 2 (NETO2) pseudogene	chr8	+
[346]	ZBTB9	ENSG00000213588.6	-2.17351	0.00376	zinc finger and BTB domain containing 9 [Source:HGNC Symbol;Acc:HGNC:28323]	chr6	+
[347]	AL360181.3	ENSG00000254536.2	-2.17259	0.004113	novel protein	chr10	+
[348]	SNORA74D	ENSG00000252213.1	-2.17254	0.004133	small nucleolar RNA, H/ACA box 74D [Source:HGNC Symbol;Acc:HGNC:52219]	chr5	+
[349]	VTRNA1-3	ENSG00000202515.1	-2.17174	0.003016	vault RNA 1-3 [Source:HGNC Symbol;Acc:HGNC:12656]	chr5	+
[350]	RRM2P3	ENSG00000214018.3	-2.17109	3.40E-18	ribonucleotide reductase M2 polypeptide pseudogene 3 [Source:HGNC Symbol;Acc:HGNC:10455]	chrX	-
[351]	KIF20B	ENSG00000138182.14	-2.17065	0	kinesin family member 20B [Source:HGNC Symbol;Acc:HGNC:7212]	chr10	+
[352]	TACC3	ENSG00000013810.19	-2.17034	2.21E-102	transforming acidic coiled-coil containing protein 3 [Source:HGNC Symbol;Acc:HGNC:11524]	chr4	+
[353]	CLCN3P1	ENSG00000232000.3	-2.16808	0.003387	chloride voltage-gated channel 3 pseudogene 1 [Source:HGNC Symbol;Acc:HGNC:49775]	chr9	-
[354]	RN7SL836P	ENSG00000241226.3	-2.16274	1.58E-05	RNA, 7SL, cytoplasmic 836, pseudogene [Source:HGNC Symbol;Acc:HGNC:46852]	chr19	+

[355]	CKS1BP6	ENSG00000178556.8	-2.16246	9.52E-05	CDC28 protein kinase regulatory subunit 1B pseudogene 6 [Source:HGNC Symbol;Acc:HGNC:24608]	chrX	+
[356]	HIST2H2AC	ENSG00000184260.5	-2.16241	0	H2A clustered histone 20 [Source:HGNC Symbol;Acc:HGNC:4738]	chr1	+
[357]	DIAPH3	ENSG00000139734.19	-2.16228	4.75E-53	diaphanous related formin 3 [Source:HGNC Symbol;Acc:HGNC:15480]	chr13	-
[358]	CCNB2	ENSG00000157456.8	-2.16101	1.32E-92	cyclin B2 [Source:HGNC Symbol;Acc:HGNC:1580]	chr15	+
[359]	AC079949.3	ENSG00000286016.1	-2.16005	0.004383	novel transcript	chr12	+
[360]	RN7SL678P	ENSG00000263969.2	-2.15974	2.03E-09	RNA, 7SL, cytoplasmic 678, pseudogene [Source:HGNC Symbol;Acc:HGNC:46694]	chr21	+
[361]	RN7SL608P	ENSG00000239884.3	-2.15829	2.86E-09	RNA, 7SL, cytoplasmic 608, pseudogene [Source:HGNC Symbol;Acc:HGNC:46624]	chr17	+
[362]	MSH5-SAPCD1	ENSG00000255152.8	-2.15798	0.002583	MSH5-SAPCD1 readthrough (NMD candidate) [Source:HGNC Symbol;Acc:HGNC:41994]	chr6	+
[363]	FJX1	ENSG00000179431.7	-2.15583	1.56E-58	four-jointed box kinase 1 [Source:HGNC Symbol;Acc:HGNC:17166]	chr11	+
[364]	H1FX-AS1	ENSG00000206417.8	-2.15457	0.002172	H1-10 antisense RNA 1 [Source:HGNC Symbol;Acc:HGNC:27953]	chr3	+
[365]	AC015813.5	ENSG00000279069.1	-2.1538	5.24E-09	novel transcript	chr17	+
[366]	SLC19A1	ENSG00000173638.19	-2.15257	4.11E-27	solute carrier family 19 member 1 [Source:HGNC Symbol;Acc:HGNC:10937]	chr21	-
[367]	KIAA1549L	ENSG00000110427.16	-2.14803	6.85E-102	KIAA1549 like [Source:HGNC Symbol;Acc:HGNC:24836]	chr11	+
[368]	MIR1291	ENSG00000281842.2	-2.14481	0.004012	microRNA 1291 [Source:HGNC Symbol;Acc:HGNC:35284]	chr12	-
[369]	CEP55	ENSG00000138180.16	-2.1446	2.28E-212	centrosomal protein 55 [Source:HGNC Symbol;Acc:HGNC:1161]	chr10	+

[370]	HASPIN	ENSG00000177602.5	-2.14229	5.81E-31	histone H3 associated protein kinase [Source:HGNC Symbol;Acc:HGNC:19682]	chr17	+
[371]	GINS4	ENSG00000147536.12	-2.14011	3.01E-27	GINS complex subunit 4 [Source:HGNC Symbol;Acc:HGNC:28226]	chr8	+
[372]	ZP1	ENSG00000149506.11	-2.13631	0.001225	zona pellucida glycoprotein 1 [Source:HGNC Symbol;Acc:HGNC:13187]	chr11	+
[373]	ACPP	ENSG00000014257.16	-2.13611	3.78E-06	acid phosphatase 3 [Source:HGNC Symbol;Acc:HGNC:125]	chr3	+
[374]	APOBEC3B	ENSG00000179750.16	-2.13042	8.56E-28	apolipoprotein B mRNA editing enzyme catalytic subunit 3B [Source:HGNC Symbol;Acc:HGNC:17352]	chr22	+
[375]	TONSL	ENSG00000160949.17	-2.1261	3.11E-56	tonsoku like, DNA repair protein [Source:HGNC Symbol;Acc:HGNC:7801]	chr8	-
[376]	HIST1H2BK	ENSG00000197903.7	-2.12532	5.90E-123	H2B clustered histone 12 [Source:HGNC Symbol;Acc:HGNC:13954]	chr6	-
[377]	ERG	ENSG00000157554.19	-2.12255	0.001912	ETS transcription factor ERG [Source:HGNC Symbol;Acc:HGNC:3446]	chr21	-
[378]	RN7SKP90	ENSG00000199883.1	-2.11999	2.63E-07	RN7SK pseudogene 90 [Source:HGNC Symbol;Acc:HGNC:45814]	chr11	+
[379]	CIT	ENSG00000122966.16	-2.1176	1.10E-221	citron rho-interacting serine/threonine kinase [Source:HGNC Symbol;Acc:HGNC:1985]	chr12	-
[380]	RN7SL277P	ENSG00000240490.3	-2.11672	0.000413	RNA, 7SL, cytoplasmic 277, pseudogene [Source:HGNC Symbol;Acc:HGNC:46293]	chr1	+
[381]	TICRR	ENSG00000140534.14	-2.11388	1.07E-46	TOPBP1 interacting checkpoint and replication regulator [Source:HGNC Symbol;Acc:HGNC:28704]	chr15	+
[382]	EMP2	ENSG00000213853.10	-2.11262	2.04E-77	epithelial membrane protein 2 [Source:HGNC Symbol;Acc:HGNC:3334]	chr16	-
[383]	SKA1	ENSG00000154839.10	-2.11206	3.36E-27	spindle and kinetochore associated complex subunit 1 [Source:HGNC Symbol;Acc:HGNC:28109]	chr18	+

[384]	RN7SL646P	ENSG00000242341.3	-2.11204	4.74E-07	RNA, 7SL, cytoplasmic 646, pseudogene [Source:HGNC Symbol;Acc:HGNC:46662]	chr5	+
[385]	CCDC178	ENSG00000166960.16	-2.11017	0.00559	coiled-coil domain containing 178 [Source:HGNC Symbol;Acc:HGNC:29588]	chr18	-
[386]	CLDN2	ENSG00000165376.10	-2.10877	0.004234	claudin 2 [Source:HGNC Symbol;Acc:HGNC:2041]	chrX	+
[387]	RN7SL322P	ENSG00000276588.1	-2.10846	0.005638	RNA, 7SL, cytoplasmic 322, pseudogene [Source:HGNC Symbol;Acc:HGNC:46338]	chr19	+
[388]	LRRC45	ENSG00000169683.8	-2.10385	2.83E-33	leucine rich repeat containing 45 [Source:HGNC Symbol;Acc:HGNC:28302]	chr17	+
[389]	AC006128.1	ENSG00000279716.1	-2.10381	0.000188	TEC	chr19	+
[390]	PHF5CP	ENSG00000267178.3	-2.10015	0.001538	PHD finger protein 5C pseudogene [Source:HGNC Symbol;Acc:HGNC:32385]	chr19	-
[391]	SLC31A2	ENSG00000136867.11	-2.09959	0.002054	solute carrier family 31 member 2 [Source:HGNC Symbol;Acc:HGNC:11017]	chr9	+
[392]	RN7SL166P	ENSG00000275014.1	-2.09748	1.75E-14	RNA, 7SL, cytoplasmic 166, pseudogene [Source:HGNC Symbol;Acc:HGNC:46182]	chr13	-
[393]	SPAG5	ENSG00000076382.17	-2.09611	2.58E-225	sperm associated antigen 5 [Source:HGNC Symbol;Acc:HGNC:13452]	chr17	-
[394]	RN7SL862P	ENSG00000242651.3	-2.0944	0.000133	RNA, 7SL, cytoplasmic 862, pseudogene [Source:HGNC Symbol;Acc:HGNC:46878]	chr18	-
[395]	RN7SKP185	ENSG00000199683.1	-2.09437	1.14E-05	RN7SK pseudogene 185 [Source:HGNC Symbol;Acc:HGNC:45909]	chr20	-
[396]	AC055840.1	ENSG00000286890.1	-2.09058	0.006152	novel transcript, antisense to FHOD3	chr18	-
[397]	KIF18A	ENSG00000121621.7	-2.08847	1.94E-118	kinesin family member 18A [Source:HGNC Symbol;Acc:HGNC:29441]	chr11	-
[398]	CDC45	ENSG00000093009.10	-2.0873	1.24E-53	cell division cycle 45 [Source:HGNC Symbol;Acc:HGNC:1739]	chr22	+
[399]	HIST2H3PS2	ENSG00000273213.3	-2.08157	0.000612	H3.2 histone (putative) [Source:HGNC Symbol;Acc:HGNC:32060]	chr1	-

[400]	KDR	ENSG00000128052.10	-2.08051	3.00E-23	kinase insert domain receptor [Source:HGNC Symbol;Acc:HGNC:6307]	chr4	-
[401]	OLFML3	ENSG00000116774.12	-2.07997	1.78E-15	olfactomedin like 3 [Source:HGNC Symbol;Acc:HGNC:24956]	chr1	+
[402]	PRR7	ENSG00000131188.12	-2.07992	8.18E-05	proline rich 7, synaptic [Source:HGNC Symbol;Acc:HGNC:28130]	chr5	+
[403]	RN7SL521P	ENSG00000240877.3	-2.07862	1.24E-06	RNA, 7SL, cytoplasmic 521, pseudogene [Source:HGNC Symbol;Acc:HGNC:46537]	chr7	-
[404]	TBX2	ENSG00000121068.14	-2.07671	7.41E-58	T-box transcription factor 2 [Source:HGNC Symbol;Acc:HGNC:11597]	chr17	+
[405]	RNU4-82P	ENSG00000199313.1	-2.0705	0.005228	RNA, U4 small nuclear 82, pseudogene [Source:HGNC Symbol;Acc:HGNC:47018]	chr9	-
[406]	RPS29P5	ENSG00000230777.1	-2.07006	0.000264	ribosomal protein S29 pseudogene 5 [Source:HGNC Symbol;Acc:HGNC:35584]	chr1	+
[407]	BIRC5	ENSG00000089685.15	-2.06784	1.71E-221	baculoviral IAP repeat containing 5 [Source:HGNC Symbol;Acc:HGNC:593]	chr17	+
[408]	LRRTM4	ENSG00000176204.13	-2.0637	0.000362	leucine rich repeat transmembrane neuronal 4 [Source:HGNC Symbol;Acc:HGNC:19411]	chr2	-
[409]	KRT75	ENSG00000170454.6	-2.06189	4.35E-24	keratin 75 [Source:HGNC Symbol;Acc:HGNC:24431]	chr12	-
[410]	AL138789.1	ENSG00000233589.1	-2.06016	0.003795	novel transcript	chr1	+
[411]	AC016596.3	ENSG00000264281.3	-2.05939	1.29E-13	ribosomal protein L41 (RPL41) pseudogene	chr5	+
[412]	EME1	ENSG00000154920.14	-2.05894	1.12E-15	essential meiotic structure-specific endonuclease 1 [Source:HGNC Symbol;Acc:HGNC:24965]	chr17	+
[413]	RN7SL230P	ENSG00000264916.2	-2.05857	2.17E-07	RNA, 7SL, cytoplasmic 230, pseudogene [Source:HGNC Symbol;Acc:HGNC:46246]	chr1	+
[414]	KCNA7	ENSG00000104848.1	-2.05629	2.69E-07	potassium voltage-gated channel subfamily A member 7 [Source:HGNC Symbol;Acc:HGNC:6226]	chr19	-

[415]	RN7SL714P	ENSG00000241757.3	-2.05343	5.26E-07	RNA, 7SL, cytoplasmic 714, pseudogene [Source:HGNC Symbol;Acc:HGNC:46730]	chr14	-
[416]	AUNIP	ENSG00000127423.10	-2.05253	6.69E-15	aurora kinase A and ninein interacting protein [Source:HGNC Symbol;Acc:HGNC:28363]	chr1	-
[417]	ESPL1	ENSG00000135476.11	-2.05048	1.26E-84	extra spindle pole bodies like 1, separase [Source:HGNC Symbol;Acc:HGNC:16856]	chr12	+
[418]	LYPLAL1-AS1	ENSG00000228536.3	-2.04829	7.27E-09	LYPLAL1 antisense RNA 1 [Source:HGNC Symbol;Acc:HGNC:54054]	chr1	-
[419]	RPL41P5	ENSG00000256393.1	-2.04614	2.03E-13	ribosomal protein L41 pseudogene 5 [Source:HGNC Symbol;Acc:HGNC:10355]	chr12	-
[420]	LINC02813	ENSG00000225334.3	-2.04547	0.00757	long intergenic non-protein coding RNA 2813 [Source:HGNC Symbol;Acc:HGNC:54345]	chr1	+
[421]	RN7SL705P	ENSG00000243549.3	-2.04521	0.001584	RNA, 7SL, cytoplasmic 705, pseudogene [Source:HGNC Symbol;Acc:HGNC:46721]	chr18	-
[422]	AC005884.2	ENSG00000267449.1	-2.04216	2.70E-07	novel transcript, antisense to BCAS3	chr17	-
[423]	ERCC6L	ENSG00000186871.7	-2.04194	7.96E-48	ERCC excision repair 6 like, spindle assembly checkpoint helicase [Source:HGNC Symbol;Acc:HGNC:20794]	chrX	-
[424]	AC125807.2	ENSG00000250899.3	-2.04123	1.95E-08	novel transcript	chr12	+
[425]	ZWINT	ENSG00000122952.17	-2.03962	6.68E-225	ZW10 interacting kinetochore protein [Source:HGNC Symbol;Acc:HGNC:13195]	chr10	-
[426]	MROH6	ENSG00000204839.9	-2.03791	0.002577	maestro heat like repeat family member 6 [Source:HGNC Symbol;Acc:HGNC:27814]	chr8	-
[427]	RN7SKP91	ENSG00000222784.1	-2.03784	0.006164	RN7SK pseudogene 91 [Source:HGNC Symbol;Acc:HGNC:45815]	chr1	+
[428]	Metazoa_SRP	ENSG00000276213.1	-2.03641	4.24E-07	Metazoan signal recognition particle RNA [Source:RFAM;Acc:RF00017]	chr7	-
[429]	RN7SL803P	ENSG00000272197.2	-2.03634	3.34E-07	RNA, 7SL, cytoplasmic 803, pseudogene [Source:HGNC Symbol;Acc:HGNC:46819]	chr5	-

[430]	HIST1H3E	ENSG00000274750.2	-2.03361	5.61E-25	H3 clustered histone 6 [Source:HGNC Symbol;Acc:HGNC:4769]	chr6	+
[431]	RN7SL480P	ENSG00000266187.2	-2.03099	5.81E-12	RNA, 7SL, cytoplasmic 480, pseudogene [Source:HGNC Symbol;Acc:HGNC:46496]	chr1	+
[432]	HAUS7	ENSG00000213397.10	-2.02974	0.000716	HAUS augmin like complex subunit 7 [Source:HGNC Symbol;Acc:HGNC:32979]	chrX	-
[433]	RN7SL804P	ENSG00000266185.2	-2.02719	3.56E-05	RNA, 7SL, cytoplasmic 804, pseudogene [Source:HGNC Symbol;Acc:HGNC:46820]	chr12	+
[434]	SNORD3A	ENSG00000263934.4	-2.02629	0	small nucleolar RNA, C/D box 3A [Source:HGNC Symbol;Acc:HGNC:33189]	chr17	+
[435]	RN7SL30P	ENSG00000243845.3	-2.02603	0.000345	RNA, 7SL, cytoplasmic 30, pseudogene [Source:HGNC Symbol;Acc:HGNC:46046]	chr9	-
[436]	AC018512.1	ENSG00000262560.1	-2.02552	0.008053	novel protein	chr15	-
[437]	RN7SL664P	ENSG00000265078.2	-2.02513	8.28E-08	RNA, 7SL, cytoplasmic 664, pseudogene [Source:HGNC Symbol;Acc:HGNC:46680]	chr3	-
[438]	ASF1B	ENSG00000105011.9	-2.02117	2.72E-14	anti-silencing function 1B histone chaperone [Source:HGNC Symbol;Acc:HGNC:20996]	chr19	-
[439]	RN7SL296P	ENSG00000275090.1	-2.01597	0.002958	RNA, 7SL, cytoplasmic 296, pseudogene [Source:HGNC Symbol;Acc:HGNC:46312]	chr3	+
[440]	RN7SL320P	ENSG00000243900.3	-2.01525	0.000697	RNA, 7SL, cytoplasmic 320, pseudogene [Source:HGNC Symbol;Acc:HGNC:46336]	chr13	+
[441]	SAPCD2	ENSG00000186193.9	-2.01492	3.83E-75	suppressor APC domain containing 2 [Source:HGNC Symbol;Acc:HGNC:28055]	chr9	-
[442]	AC135178.4	ENSG00000265749.6	-2.01452	0.007066	novel transcript, antisense to KRBA2 and RPL26	chr17	+
[443]	RN7SL192P	ENSG00000276757.1	-2.01244	0.00458	RNA, 7SL, cytoplasmic 192, pseudogene [Source:HGNC Symbol;Acc:HGNC:46208]	chr19	+
[444]	CSPG4P13	ENSG00000260139.6	-2.01039	6.11E-07	chondroitin sulfate proteoglycan 4 pseudogene 13 [Source:HGNC Symbol;Acc:HGNC:49195]	chr15	+

[445]	BORA	ENSG00000136122.17	-2.00864	1.35E-36	BORA aurora kinase A activator [Source:HGNC Symbol;Acc:HGNC:24724]	chr13	+
[446]	MAD1L1	ENSG00000002822.15	-2.00541	0.002955	mitotic arrest deficient 1 like 1 [Source:HGNC Symbol;Acc:HGNC:6762]	chr7	-
[447]	RN7SL523P	ENSG00000243976.2	-2.00294	0.00334	RNA, 7SL, cytoplasmic 523, pseudogene [Source:HGNC Symbol;Acc:HGNC:46539]	chr14	-
[448]	NRGN	ENSG00000154146.13	-2.00071	8.21E-05	neurogranin [Source:HGNC Symbol;Acc:HGNC:8000]	chr11	+
